# Interpretable AI-assisted diagnosis of papillary thyroid cancer cytopathology using graph neural networks and knowledge graphs

**DOI:** 10.1038/s41598-025-18235-z

**Published:** 2025-09-01

**Authors:** Li-xue Wu, Yong Jiang, Tian-you Luo, Jia-xin Hou, Yang Deng, Lu-xin Han, Ting-feng Jiang, Ji Bao

**Affiliations:** 1 Department of Pathology, West China Hospital, Sichuan University, Chengdu, 610041 Sichuan China; 2 Department of Pathology, West China School of Public Health and West China Fourth Hospital, Sichuan University, Chengdu, 610041 Sichuan China; 3 West China Clinical Medical College of Sichuan University, Chengdu, 610041 Sichuan China; 4 Department of Pathology, Key Laboratory of Transplant Engineering and Immunology, West China Hospital, Institute of Clinical Pathology, Sichuan University, Chengdu, 610041 Sichuan China; 5 Sichuan KgCure Co., Ltd., Sichuan University, Chengdu, 610041 Sichuan China

**Keywords:** Papillary thyroid cancer, Fine needle aspiration, Cytology, Artificial intelligence, Graph neural networks, Knowledge graph, Interpretable artificial intelligence, Thyroid diseases, Psychology

## Abstract

This study presents an interpretable AI-assisted diagnostic approach for papillary thyroid carcinoma (PTC) cytopathology by combining graph neural networks (GNNs) with knowledge graphs (KGs). Routine cytology smears from 281 PTC cases were scanned, labeled, and processed using the Cascade RCNN model to detect pathological cell features, including 45,680 ground-glass nuclei, 712 nuclear grooves, and 116 intranuclear inclusions. By integrating GNNs, the model achieved a mean intersection over union (mIoU) of 56.14% and a mean average precision (mAP) of 0.87. The GINet model further improved classification accuracy to 88.84%. Our approach also incorporates a clinical decision support system (CDSS) for querying KGs, providing explainable diagnostic outputs. This method offers an interpretable and reliable AI tool for PTC diagnosis, enhancing the transparency of AI-assisted pathology systems.

**Supplementary Information:**

The online version contains supplementary material available at 10.1038/s41598-025-18235-z.

## Introduction

The global incidence of malignant tumors, including thyroid cancer, has been rising steadily^1^. Papillary thyroid carcinoma (PTC) is one of the most common types, and ultrasound-guided fine-needle aspiration cytology (FNAC) is widely recognized as the specific and accurate method for diagnosing thyroid nodules^2^. Wu et al.^3^ revealed that the detection rate of malignant tumors in postoperative specimens significantly increased following the implementation of fine-needle aspiration cytology (FNAC), with papillary thyroid carcinoma (PTC) showing the most pronounced growth. These findings underscore the critical role of FNAC in the diagnosis of thyroid neoplasms.

In recent years, diagnostic pathology has increasingly integrated artificial intelligence (AI) technologies, particularly convolutional neural networks (CNNs), for image analysis^4^. Sanyal et al.^5^. Using CNN for FNAC smear of the thyroid, a conclusion was drawn with a sensitivity of 90.48% and a specificity of 83.33%. Range et al.^6^ applied CNN to predict the nature of thyroid follicular epithelial cells in their study, achieving sensitivity of 92.0% and specificity of 90.5%.Although machines can achieve high rates in analyzing thyroid cytology smears through machine learning., CNN-based methods often suffer from the “black box” effect, where the lack of transparency in the decision-making process limits trust from pathologists and patients^7^. To address this, we explored graph neural networks (GNNs), which model data as graph structures, allowing the spatial relationships between features to be more clearly interpreted. GNNs, when combined with knowledge graphs (KGs), provide a promising approach to enhance the interpretability of AI-based diagnoses.

Our study aims to leverage GNNs to assist in the diagnosis of PTC, improving the transparency of AI-assisted pathology by incorporating semantic reasoning through KGs. This method provides not only accurate diagnostic results but also importantly interpretable outputs, addressing one of the critical challenges in AI-driven medical diagnostics more credible.

## Materials and methods

### Specimen source

This study reviewed thyroid fine-needle aspiration cytology (FNAC) cases from the Department of Pathology, West China Hospital, Sichuan University, between April and October 2021. Pap-stained cytology smears with confirmed diagnoses of papillary thyroid carcinoma (PTC) were included, and the diagnoses were re-evaluated by thirteen clinical medicine undergraduate volunteers, along with two experienced pathologists following the Bethesda System for Reporting Thyroid Cytopathology (TBSRTC)^8^. Smears were scanned by Hamamatsu Nano Zoomer Digital Pathology(20 × 0.46 microns per pixel, 40 × 0.23 microns per pixe), and the resulting digital images (NDPI format) were divided into a training set, validation set, and test set.

### Nucleus labeling

**Fig. 1:**
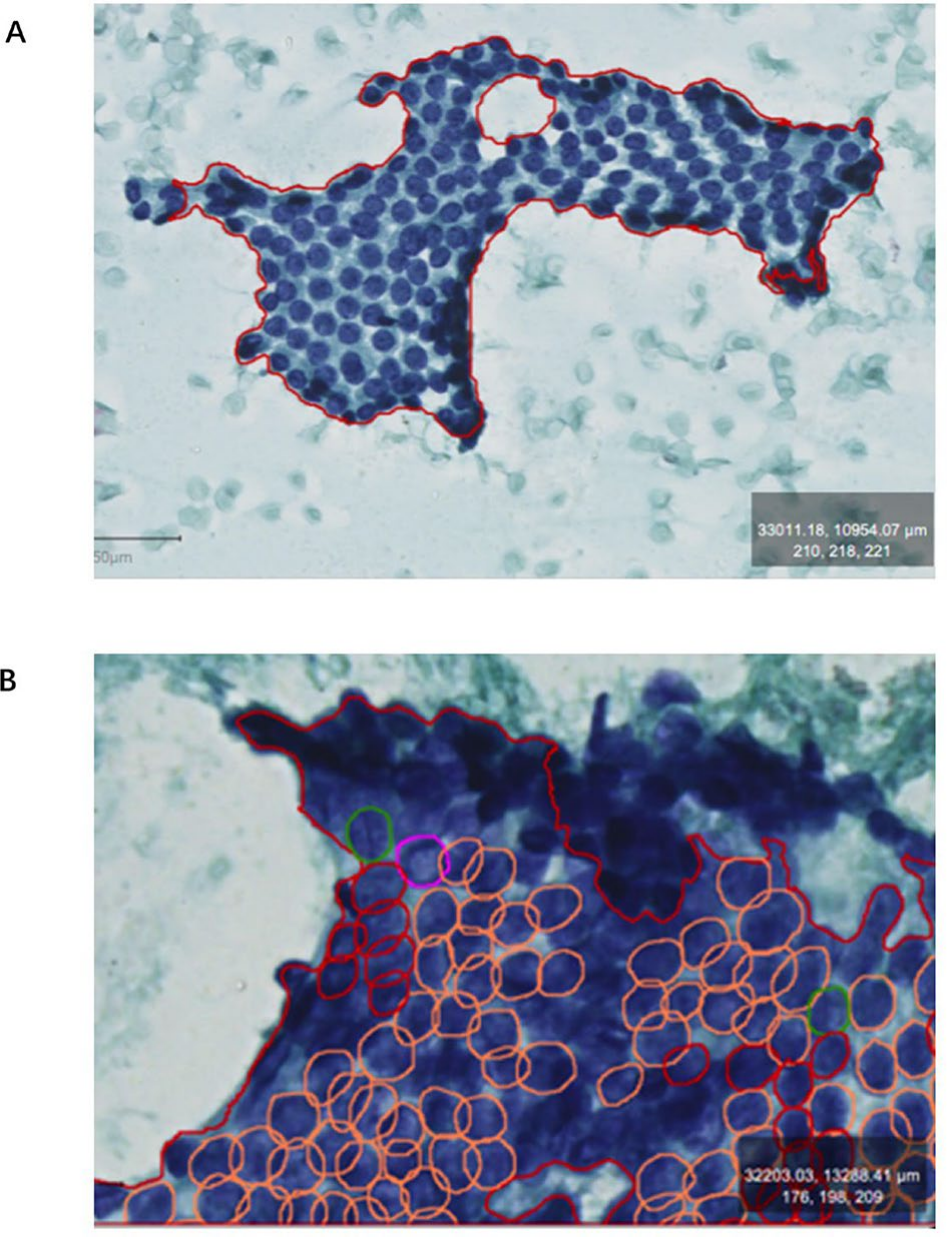
Artificial labeling of nuclei in thyroid FNAC smears.

For annotation, the cytological nucleus features of PTC, including ground-glass nuclei, nuclear grooves, intranuclear inclusions, and multinucleated macrophages, were manually labeled using QuPath software (version 0.3.0). We define the cell clusters that we wish the machine to recognize as Regions of interest (ROIs), and in which tumor cells were labeled accordingly. Two pathologists reviewed all annotations to ensure accuracy. Using manual labeling of target regions and cells, we delineated the region of interest (ROI) (Fig. 1A) of conventional smears that met the inclusion criteria, set “tumor” as a label, manually labeled the tumor along the boundary of tumor cell clusters, and labeled the nuclei of cells within the region according to the corresponding labels one by one, include nuclear grooves (green), intranuclear pseudoinclusions (pink), ground-glass nuclei (orange), multinucleated giant cells (yellow border)(Fig. 1B).

### Literature labeling

Relevant literature was sourced using the keywords “thyroid,” “papillary carcinoma,” “cytology,” and “pathology”. Fourteen studies (Table 1) were selected based on their relevance to PTC cytopathological features, such as nuclear crowding and intranuclear pseudoinclusions. The semantic types from the literature were mapped to the cytological features in the digital images using a knowledge graph (KG) approach.

**Table 1:** Annotated list of literature.

		Author	Publication time
1	Clear-cell variant of papillary thyroid carcinoma: a clinicopathological study	Jiawei et al.	2013
2	The Diagnostic Value of Tissue Blocks for Papillary Thyroid Carcinoma	Xiangrong Hu et al.	2015
3	The diagnostic value of fine-needle aspiration cytology for papillary thyroid carcinoma	Youyou Zhu et al.	2015
4	Application of Bethesda system for reporting cytology in thyroid fine needle aspiration	Yun Cai et al.	2015
5	Analysis of 15 cases of missed and misdiagnosed thyroid fine needle aspiration cytology	Fang Liu et al.	2017
6	Application of the Bethesda system for reporting thyroid cytopathology in the diagnosis of thyroid nodule	Xiaoqiu Zhu et al.	2017
7	Analysis of Pathological Features of Papillary Thyroid Carcinoma Diagnosed by Needle Aspiration Cytology	Fei Wang et al.	2018
8	Fine needle aspiration cytological analysis of follicular papillary thyroid carcinoma	Li Qiu et al.	2018
9	Columnar cell variant of papillary thyroid carcinoma: a clinicopathologic analysis of 4 cases	Xing Zhao et al.	2019
10	Application and diagnostic value of cell masses and cytology in papillary thyroid carcinoma	Yanrui Ma	2019
11	Retrospective study on Bethesda Ⅴ thyroid nodule in diagnosis of papillary thyroid carcinoma: retrospective study	Shouyi Yan et al.	2021
12	Analysis of characteristics of ultrasound and fine needle aspiration cytology in patients with diffuse sclerosing variant of papillary thyroid carcinoma	Tiantian Xu et al.	2022
13	Cytological characteristics of columnar cell variant of papillary thyroid carcinoma with fine-needle aspiration	Futing Tang et al.	2022
14	Fine needle aspiration cytology of thyroid follicular tumor: a clinicopathological analysis of 36 cases	Jue Wang et al.	2022

**Table 2:** Cascade-R-CNN-based model performance.

IoU	Accuracy	Recall rate	Precision	F1 Index	(Level of) sensitivity	Specificity
0.5	0.7139	0.7502	0.6922	0.7200	0.7502	0.4096
0.75	0.6819	0.6696	0.6319	0.6502	0.6696	0.4401

*Note that specificity is the probability of correctly determining nondetects in a sample; normal target detection is not used for nondetects, so the specificity data for target detection are for reference only.

**Table 3:** Validation results for each classification.

	The background components included multinucleated macrophages.	Ground-glass nuclei	Nuclear grooves	Intranuclear pseudoinclusions
Precision	0.9770	0.6359	0.9139	overfitting
Recall	0.9064	0.7062	0.5343	overfitting
IoU	0.8875	0.5029	0.5087	overfitting
Specificity	0.9791	0.7121	0.9521	0.9457
F1	0.9404	0.6692	0.6743	overfitting

*We classified multinucleated macrophages by combining them with the background due to their small composition and scattered distribution.

### Cellular target detection via cascade CNN

The Cascade CNN model was employed for cell detection.

**Fig. 2:**
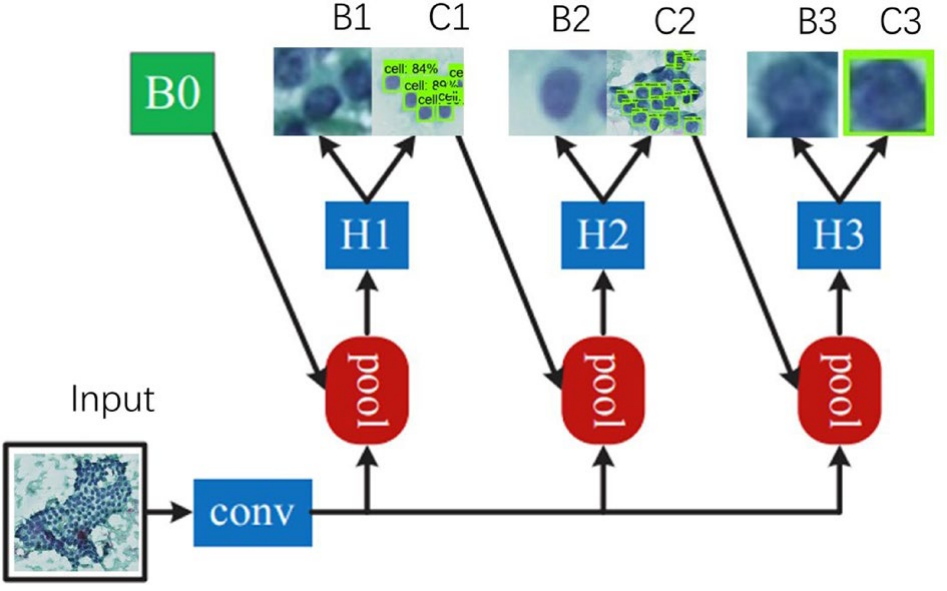
Architecture of Cascade-R-CNN. The term “conv” denotes backbone convolution, “pool” denotes region feature extraction, “H” denotes the network head, “B” denotes the bounding box, and “C” denotes the classification. “B0” denotes the feature in all architectures.

**Fig. 3:**
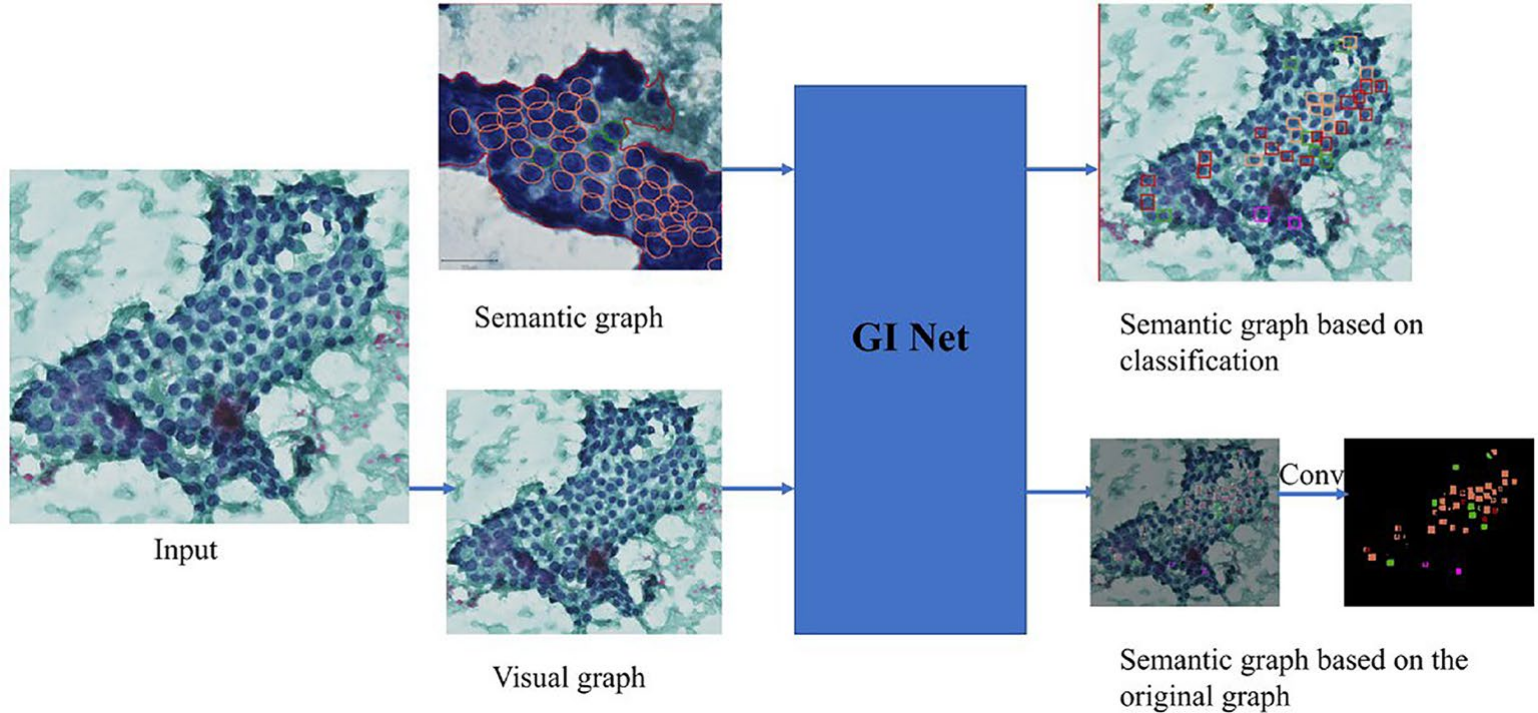
Schematic diagram of GINet, a semantic feature-based graph inference approach.

1. The annotated image is processed through the backbone network to obtain a feature map.
2. The obtained feature map is fed into two branches, one of which is input to the region generation network༈RPN༉, After the prediction by the RPN network, B0 is obtained in the figure, which is the coordinates of certain features on the ROI feature map. Another one is input into the pool network shown in the figure.
3. The obtained B0 is extracted from the corresponding position features in the feature map, and then fed into the pool for ROI pooling, resulting in a local feature map with a fixed width and height, the feature map is a local part of the feature map output from the backbone instead of channel.
4. Input the local feature map into H1 for classification and regression to get C1 and B1.
5. Subsequently, utilizing the coordinates of B1, local features are extracted from the original feature map org f. The extracted local features are then fed into H2. Notably, the Intersection over Union (IoU) in H2 is higher compared to that in H1. Consequently, through the processing by H2, C2 and B2 are derived.
6. Based on the coordinates of B2, local features are extracted from the original feature map org f and subsequently input into H3. Notably, the IoU in H3 is the highest among all stages, leading to the derivation of C3 and B3 through the processing by H3.
7. C3 and B3 are designated as the final results.(Fig. 2).

### Graph neural network (GINet) for feature-based inference

GINet was used to classify cytological features based on semantic graphs:

1. By utilizing a pre-trained Residual Network (ResNet) as the backbone network, visual features (visual representations) are extracted from 2D input images. Additionally, semantic knowledge derived from the dataset is captured in the form of classification instances (classes) and subsequently transformed into word embeddings to achieve robust semantic representations.
2. The visual features and semantic representations are projected by the proposed GI unit to construct two distinct graphs: a visual graph and a semantic graph. The visual graph encodes dependencies between visual regions, with nodes representing individual visual regions and edges capturing the similarity or relationships between these regions. The semantic graph, constructed based on categories derived from the dataset, encodes both semantic relevance and label correlations.
3. Within the GI unit, graph interaction operations are conducted. Specifically, semantic graphs are employed to enhance contextual reasoning in visual graphs and guide the generation of semantic graphs derived from the original visual features. Subsequently, the GI unit generates evolved visual graphs, and through graph reprojection operations, the discriminative power of each local visual representation is significantly improved. Meanwhile, during the training phase, the semantic graphs are updated and constrained by the semantic context loss.
4. We employ 1 × 1 convolutional layers followed by bilinear up sampling to obtain the final parsing results.

For algorithm implementation, we utilize PaddleSeg, an end-to-end image segmentation toolkit developed based on the Paddle Paddle framework. (Fig. 3).

### Knowledge graph construction

**Fig. 4:**
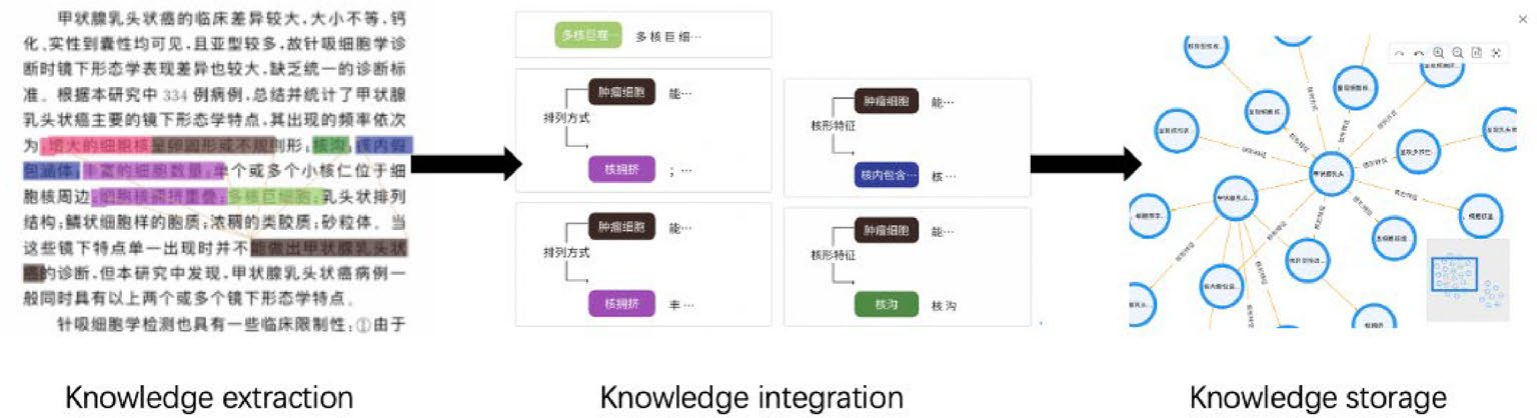
The construction process of knowledge graphs.

**Fig. 5:**
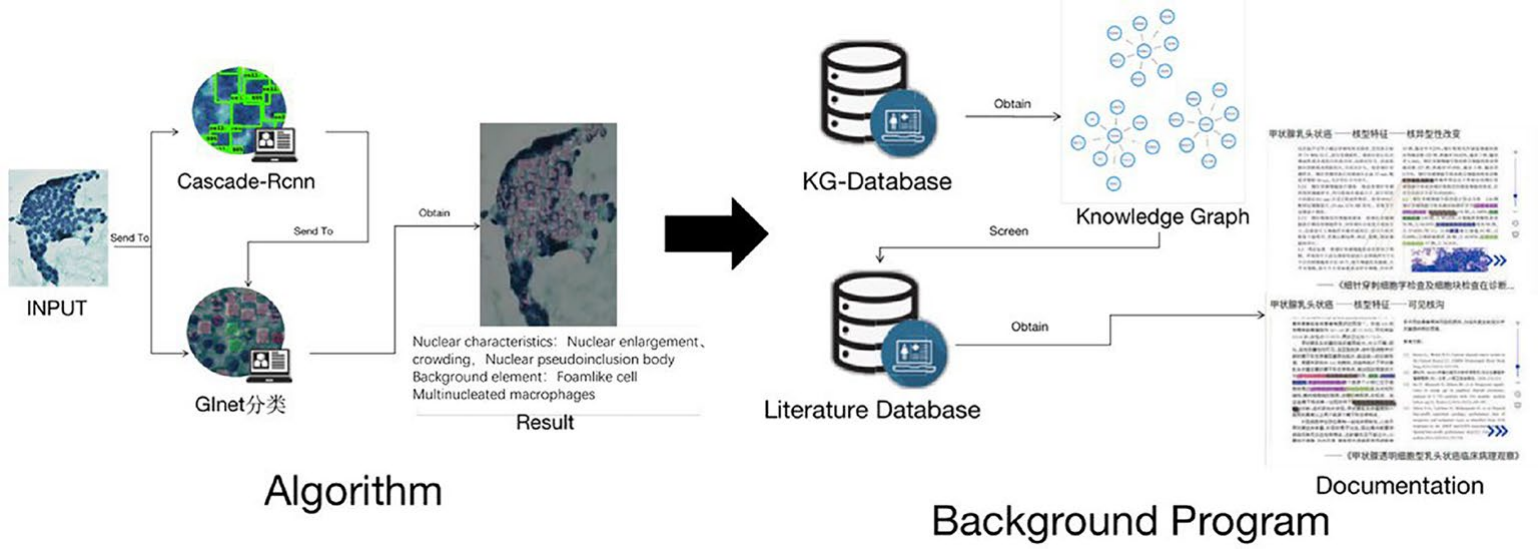
Full process model diagram of machine interpretation.

The knowledge graph data were primarily sourced from relevant literature. We utilized the spaCy library in PyTorch for entity and relationship extraction (Fig. 4). Knowledge fusion primarily addresses the challenges of entity disambiguation and coreference resolution for equivalent instances, classes/subclasses, and properties/sub properties. Given that knowledge graphs differ significantly from conventional data structures, the Neo4j graph database is utilized for efficient storage and management. Test set ROI images were processed through the Cascade CNN algorithm, and semantic features were extracted using the GINet classification model. These features were then transmitted to a background service, where the knowledge graph was queried and the literature database was screened to retrieve relevant references. The final results, including links to related literature, were generated and displayed at the end of the process (Fig. 5).

### Statistical analysis

**Fig. 6:**
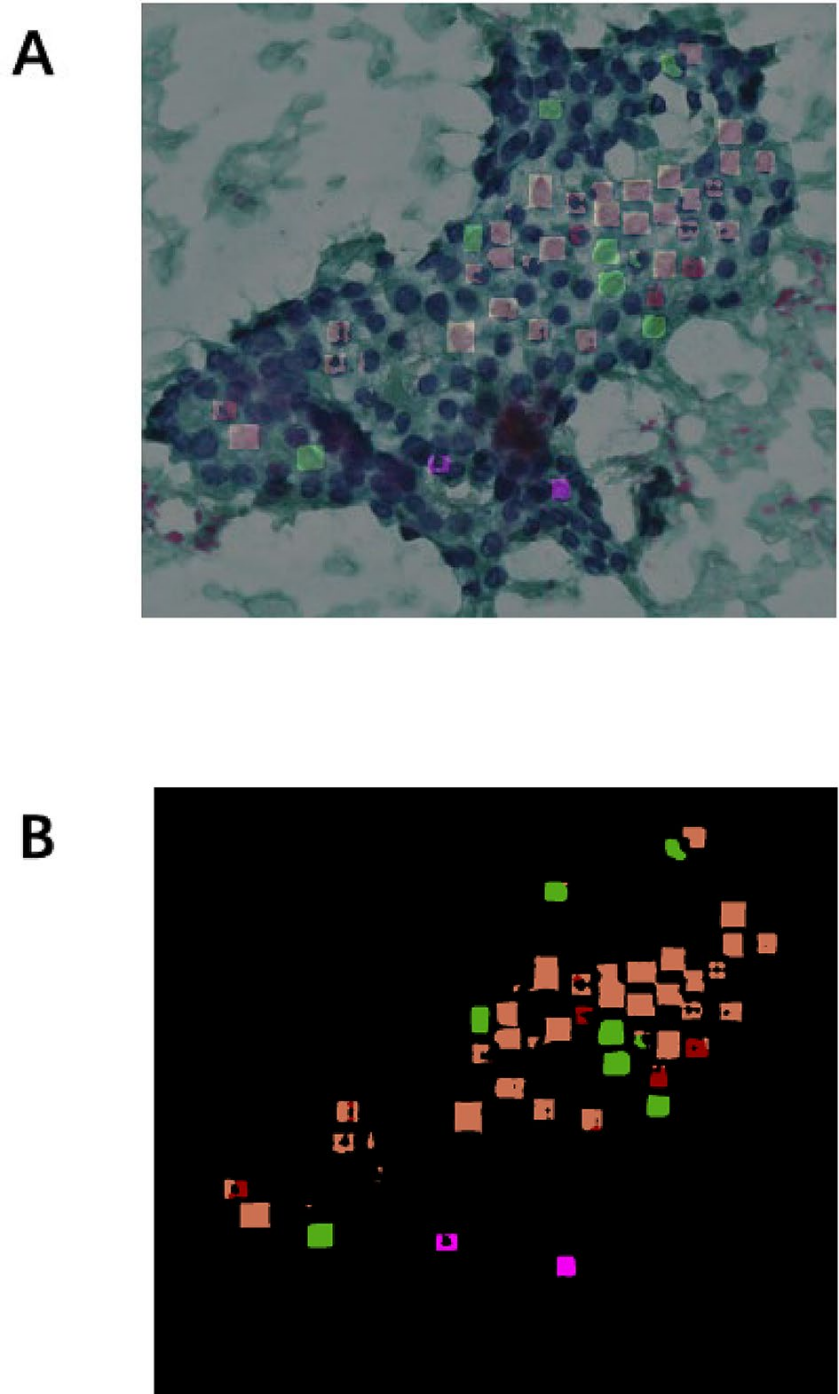
Screenshot of classification results. The original image for the C test and the corresponding mask image for the D test is provided. In the mask image, the following color codes are used: orange represents ground-glass nuclei, green represents nuclear grooves, purplish red represents intranuclear pseudoinclusions, and red represents tumor regions.

We employed intersection over union (IoU) as the primary metric for evaluating model performance. Additional metrics included mean IoU (mIoU), mean average precision (mAP), the Dice similarity coefficient (DSC), as well as accuracy, recall, precision, positive predictive value (PPV), F1-score, sensitivity, true positive rate (TPR), and specificity. Each metric produces values between 0 and 1, where values closer to 1 indicate better model performance. Currently, the “gold standard” in pathology remains based on the diagnoses provided by pathologists. Therefore, to evaluate the interpretability of the final algorithm, the original images from the test set are input into the algorithm, and its output(Figure 6A) is manually compared against the manually annotated classification results. The agreement rate between these two sets of results serves as the final matching rate. Through the corresponding mask image of classification results (Fig. 6B), we can identify the specific reasons for incorrect interpretations or recognition failures by the machine. The initial assessment was conducted by thirteen clinical medicine undergraduate volunteers, followed by a review by two pathologists.

## Results

### Nucleus labeling results

A total of 281 cytology slides were scanned, resulting in the identification of 7,246 regions of interest (ROIs), which included tumor cell clusters labeled as “Tumor”. These ROIs were split into training (5,000), validation (1,000), and test (1,246) sets. A total of 46,564 cells were manually labeled, including 45,680 ground-glass nuclei, 712 nuclear grooves, 116 intranuclear pseudoinclusions, and 56 multinucleated macrophages. This detailed annotation provided the foundation for training the detection and classification models.

### Cascade CNN model training results

**Fig. 7:**
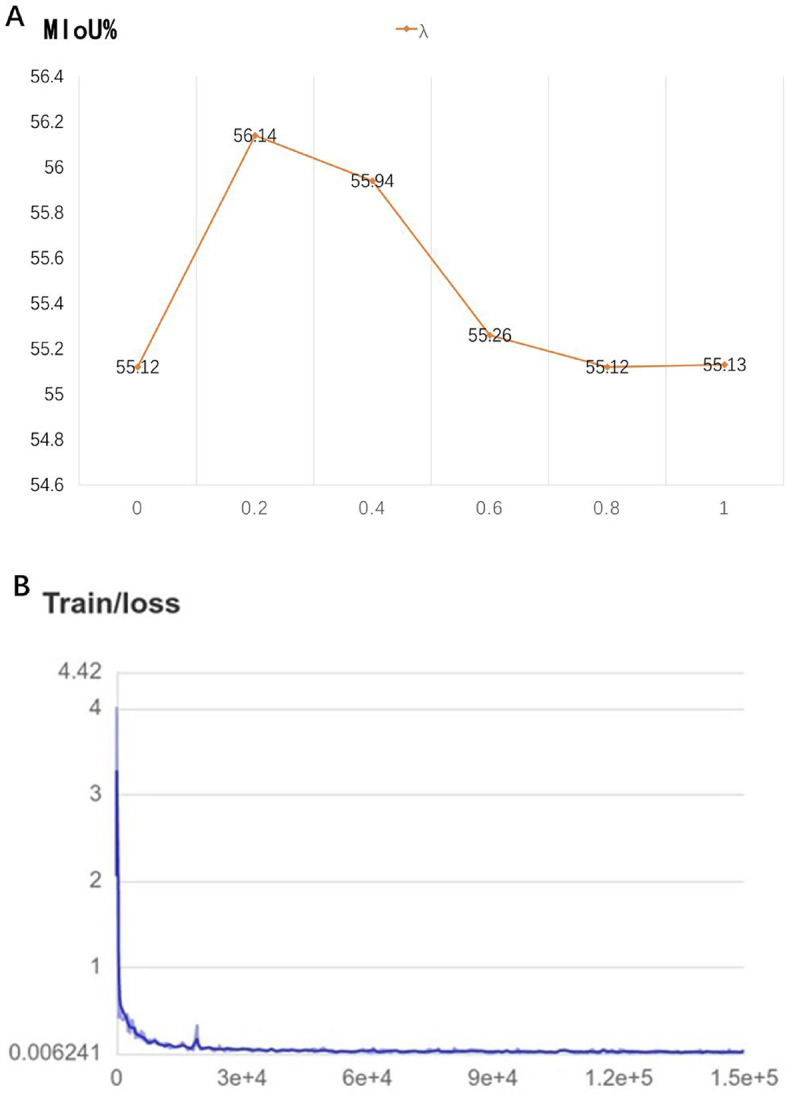
Cascade-R-CNN model IoU vs. AP relationship and model loss function curve of 1.

The Cascade CNN model was trained on the labeled dataset with intersection over union (IoU) thresholds set at 0.5 and 0.75. For the training set, the model achieved a mean average precision (mAP) of 0.89 at an IoU of 0.5, and 0.73 at an IoU of 0.75. In the validation set, mAP values were 0.87 and 0.69, respectively, indicating robust performance across both IoU thresholds. Loss function analysis showed a decreasing trend with increasing iterations, suggesting that the model was well trained (Fig. 7A and B).

### GINet classification results

GINet was employed to classify cytological features based on semantic graphs. The model achieved a mean IoU (mIoU) of 56.14% and a Dice similarity coefficient of 64.70%, with an overall classification accuracy of 88.84%. When compared to other embedding methods (FastText, GoogleNews, GloVe), FastText performed slightly better with an mIoU of 56.14%, as opposed to 56.13% and 56.09% for Google News and GloVe, respectively.

### Model validation

The trained models were validated using the test set, where the Cascade CNN achieved an accuracy of 71.39%, recall of 75.02%, precision of 69.22%, and an F1-score of 72.00% at an IoU threshold of 0.5. At an IoU of 0.75, the performance metrics showed slight reductions, with an accuracy of 68.19%, recall of 66.96%, precision of 63.19%, and F1-score of 65.02% (Table 2).

### GINet overfitting

Although GINet achieved high accuracy in classifying most cytological features, some signs of overfitting were observed, particularly in the classification of intranuclear pseudoinclusions. Overfitting affected the model’s ability to generalize for rarer features, though the overall accuracy remained high.

### Knowledge graph query results

**Fig. 8:**
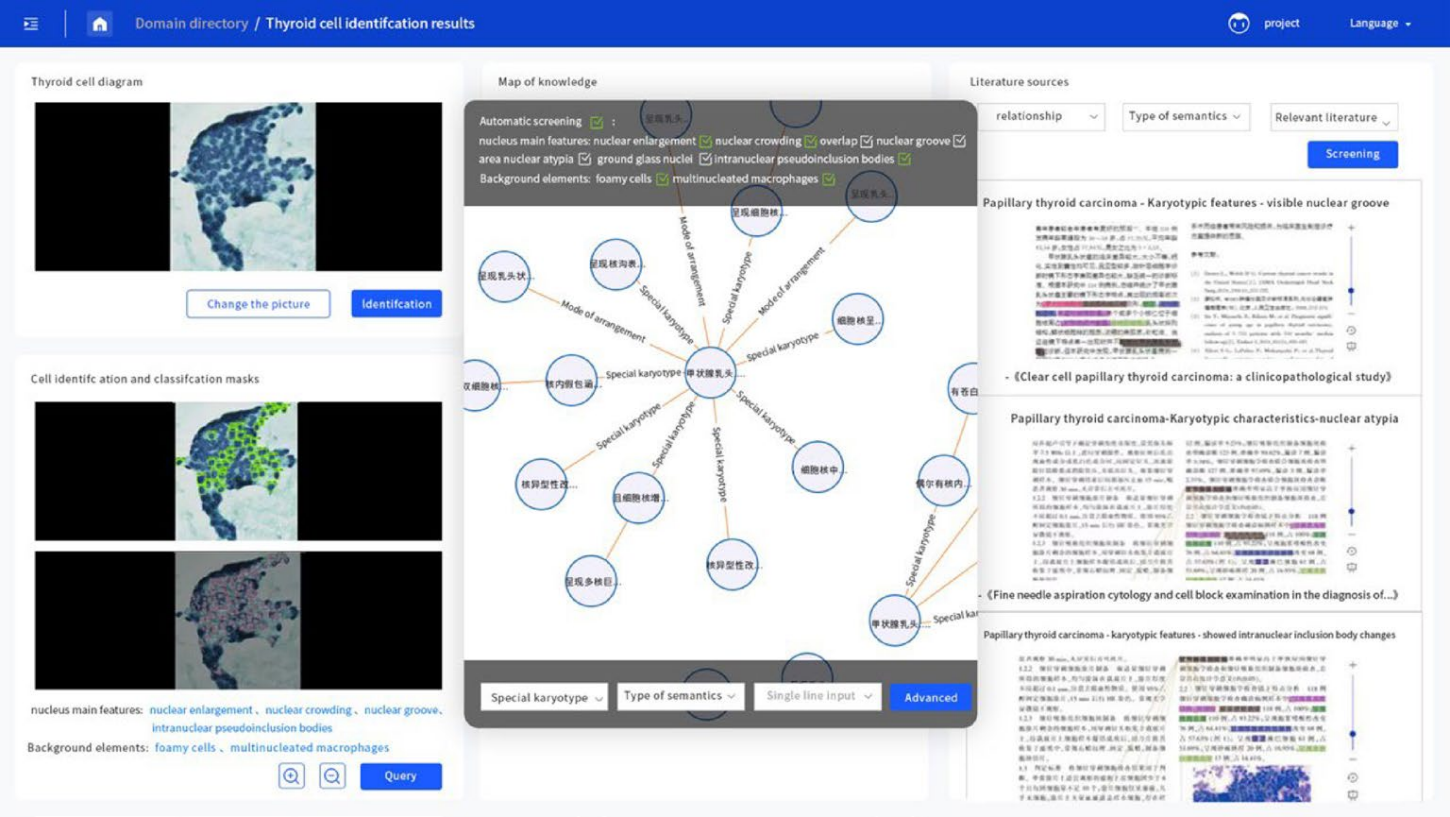
Screenshot of the interface of the CDSS software interpretation of the FNAC results. The original map identification is added to the upper left. After automatic identification, the lower left target classification map is constructed. The middle figure shows the tumor cell-related knowledge map that is constructed after the semantic type of the classification information for the knowledge map query is identified; CDSS automatically checks the classification of the semantic type at the top (the labels can also be omitted manually) and on the right side of the descriptive information of the literature.

The knowledge graph queries, generated from the results of the GINet and CascadeRCNN models, yielded an 88.21% compliance rate with the relevant literature and cytological findings. These results demonstrate the effectiveness of the knowledge graph system in providing interpretability and linking diagnostic features to literature references (Fig. 8).

### GINet classification results

GINet was used to classify cytological features by constructing semantic graphs. The model achieved a mean intersection over union (mIoU) of 56.14% and a Dice similarity coefficient of 64.70%, with an overall classification accuracy of 88.84%. Among the embedding methods tested, FastText slightly outperformed Google News and GloVe, with mIoU scores of 56.14%, 56.13%, and 56.09%, respectively.

We applied the GINet model, integrated with Fast Text embeddings, to the validation set and achieved a classification accuracy of 88.84%. Table 3 summarizes the precision, recall, IoU, specificity, and F1 values for each classification category. While most features were accurately classified, the model showed signs of overfitting in the classification of intranuclear pseudoinclusions, affecting the performance on this specific feature.

The graph neural network, combined with knowledge graphs, demonstrated a strong performance in classifying papillary thyroid carcinoma (PTC) in the test set. Specifically, 76.83% of the regions of interest (ROIs) achieved a tumor cell nucleus morphology classification accuracy of 88.84% or higher. However, 11.38% of the ROIs exhibited a lower classification accuracy, falling below 88.84%. Additionally, 11.79% of the ROIs could not be classified by the model. We have discovered the cause of this result likely due to the presence of pseudo-inclusion bodies and other complex cytological features.

## Discussion

The global rise in the detection of thyroid nodules has made fine-needle aspiration cytology (FNAC) the gold standard for preoperative diagnosis due to its safety and accuracy^1^. However, obtaining high-quality, labeled datasets for deep learning in pathology remains a significant challenge^9^. We have conducted a comprehensive review of the research on the pathological diagnosis of thyroid tumors over the past decade and found that the vast majority of studies have utilized CNN model algorithms. In Lakshmi Nagendra et al.’s^10^ review, it was reported that the accuracy of AI-assisted thyroid fine needle aspiration cytology in determining tumor characteristics ranged from 85.06–90.6%.(引用11)While AI has shown great potential in aiding pathological diagnosis, the challenge of interpretability persists^11^. Without transparency, clinicians and patients may be reluctant to trust AI-assisted diagnoses, particularly in critical health-related fields. Efforts to improve AI interpretability must continue to address this “black box” issue, as emphasized by various researchers^12^. This issue is also prevalent across all studies utilizing CNN models.

CNNs excel in processing grid-like data. In our study, we initially input labeled images into the CNN model to perform feature extraction’s et al.^13^ compared several mainstream target detection algorithms, including YOLO^14^, SSD, and CascadeRCNN^15^. YOLO showed limitations in object position accuracy, while SSD improved on this but still struggled with smaller objects. Cascade CNN outperformed both in detecting small targets, achieving a mean average precision (mAP) of 56. Zheng et al.^16^ in their study, utilized this model to identify thyroid nodules. Building upon prior research, we applied the model to our investigation and achieved promising results. Moreover, our Cascade CNN model, when applied to our dataset, reached a higher mAP of 0.87 with an IoU threshold of 50%, likely due to the precision of our manual annotations.

The organizational structure in Whole Slide Imaging (WSI) frequently exhibits irregularity and is constrained to a limited local receptive field when capturing spatial relationships. Consequently, CNNs struggle to effectively process long-range dependencies or global structures within images. Ye et al.^17^ demonstrated in their research that GNNs incorporate an attention mechanism into the aggregation process, thereby assigning varying levels of importance to different nodes and more flexibly capturing the relationships between neighboring nodes. Several studies^18–20^ have corroborated that GNN models exhibit superior performance in handling WSIs. To better capture the spatial relationships between features, image features extracted from CNN models are subsequently processed using GNN models. The GINet Proposed by Wu et al.^21^, model designed for classifying tumor cell nucleus morphology using text embeddings, enhanced the model’s ability to capture long-term dependencies between features. They are conducting research on compared to ACNet, DANet, and EMANet, GINet achieved the best classification results with an mIoU of 54.9%. This model has mainly been used for the recognition of daily influences in previous studies^22^, we might be the first team to apply this to medical diagnosis. However, our results were slightly lower, likely due to the rarity of some markers, such as intranuclear pseudoinclusions, which led to overfitting. We selected Fast Text as the word embedding method for its adaptability and better performance in capturing semantic information^23^. Despite limited prior use in medical pathology, GINet demonstrated strong classification performance in this study, achieving a validation accuracy of 88.84%.

Finally, and most critically, to address the interpretability issue of the interpretation results, we utilized the Digital Healing CDSS system to construct a knowledge graph of PTC morphology. This system, by linking cytological features with relevant literature, allowed for keyword-driven queries and enhanced interpretability. As suggested by Li et al.^24^, knowledge graphs in medical research have developed rapidly and have the potential to reduce manual workloads, improving efficiency in clinical diagnostics.

Recent developments in artificial intelligence have emphasized the need for systems that are both interpretable and robust^25^. The focus of our study is on the interpretability of AI results, rather than explaining the inner workings of algorithms. Our post hoc interpretation approach allows pathologists to understand how AI systems can assist in diagnosing PTC. Knowledge graphs (KGs) and graph neural networks (GNNs) provide a promising solution to the “black box” problem, making AI decisions more transparent^26^.

Another challenge in AI-assisted pathology is the availability of sufficient training data. Many AI studies rely on single-center datasets, which limits the generalizability of the models^27^. Additionally, manual annotation by experts is often required to ensure accuracy, further complicating the scalability of AI systems. Although unsupervised learning offers potential solutions, large and diverse datasets are still lacking^28^. As AI technology continues to evolve, interdisciplinary collaboration between AI researchers and medical professionals will be crucial for improving diagnostic models across different fields^29^. In this study, we manually labeled cytological smears, and the accuracy of these manually labeled data far exceeded that of automatic methods. This was particularly true in cases where cell nuclei were overlapped, stretched, or distorted, making them difficult for machine learning models to classify accurately. Although manual labeling is labor-intensive, it provides a valuable reference for future AI-based cytopathology studies. In this study, we manually labeled cytological smears, and the accuracy of these manually labeled data far exceeded that of automatic methods. This was particularly true in cases where cell nuclei were overlapped, stretched, or distorted, making them difficult for machine learning models to classify accurately. Although manual labeling is labor-intensive, it provides a valuable reference for future AI-based cytopathology studies.

### Limitations of this study

Despite the promising results, this study has several limitations. First, the dataset used was relatively small and derived from a single center, which may limit the generalizability of the findings to broader clinical settings. Larger, multi-center datasets are required to fully evaluate the model’s performance across diverse populations. Second, while the combination of GNNs and knowledge graphs improved interpretability, the overfitting observed in rare cytological features, such as intranuclear pseudoinclusions, suggests that further refinement of the model is needed to handle such cases more effectively. Lastly, the reliance on manual labeling, although more accurate, is time-consuming and may not be scalable for large-scale applications. Future studies should explore automated labeling techniques or hybrid approaches that combine manual oversight with AI-based annotation to improve efficiency while maintaining accuracy.

## Conclusion

Our study demonstrates that the integration of graph neural networks (GNNs) and knowledge graphs (KGs) into AI-assisted pathology can significantly enhance the accuracy and interpretability of thyroid cytopathology diagnoses. The Cascade CNN model, supported by GINet’s karyotypic classification, proved more effective than traditional AI models in identifying tumor cells. The use of knowledge graphs further allowed for textual explanations of classification results, improving the credibility of AI-assisted diagnoses. This approach not only aids in diagnosing papillary thyroid carcinoma (PTC) but also provides valuable insights into the underlying diagnostic reasoning. These findings have the potential to inform the development of AI-assisted diagnostic tools for the full spectrum of thyroid cytopathology, advancing precision diagnostics in pathology.

## Supplementary Information

Below is the link to the electronic supplementary material.

Supplementary Material 1

## Data Availability

The datasets used and analyzed during the current study are available from the corresponding author, BJ, upon reasonable request.

## Abbreviations

AI: Artificial intelligence
CDSS: Clinical decision support system
CNN: Convolutional neural network
DSC: Dice similarity coefficient
FNAC: Fine needle aspiration cytology
GINet: Graph inference network
GNN: Graph neural network
IoU: Intersection over union
KG: Knowledge graph
mAP: Mean average precision
mIoU: Mean intersection over union
PTC: Papillary thyroid carcinoma
ROI: Region of interest
SSD: Single-shot MultiBox detector
TBSRTC: Bethesda system for reporting thyroid cytopathology
TPR: True positive rate
XAI: Explainable artificial itelligence
YOLO: You only look once
