# Supplementary material for: Interpretable AI-assisted diagnosis of papillary thyroid cancer cytopathology using graph neural networks and knowledge graphs
Source: Sci Rep. 2025 Sep 1;15:32165. doi: 10.1038/s41598-025-18235-z (PMC12402155; doi:10.1038/s41598-025-18235-z)
Supplement: Supplementary file 1 — Supplementary Material 1 [file 41598_2025_18235_MOESM1_ESM.docx]

Dear Editor and Reviewers,

As our research data involves Chinese, we have added English annotations (in parentheses) after the Chinese codes. If there are any inappropriate parts, please kindly excuse us. We are looking forward to your feedback. Thank you very much!

import Vue from 'vue'

import axios from 'axios'

import App from './App'

import G6 from '@antv/g6'

import router from './router'

import ElementUI from 'element-ui';

import 'element-ui/lib/theme-chalk/index.css';

import './css/common.css';

Vue.prototype.$axios = axios

Vue.use(ElementUI);

Vue.config.productionTip = false

/* eslint-disable no-new */

new Vue({

el: '#app',

axios,

router,

G6,

components: { App },

template: '<App/>'

})

<template>

<div class="hello">

<div class="head">

<div class="menu">

<i class="iconfont icon-gfcaidan" style="font-size:20px"></i>

</div>

<div class="home">

<i class="iconfont icon-shouye"></i>

</div>

<div class="title">

<span>域目录/</span>

<span style="color: #0b41cc;">甲状腺细胞识别结果（Thyroid cell recognition results）</span>

</div>

</div>

<div class="body">

<div class="body_left">

<div class="bl_uppon">

<div class="small_head" style="border-bottom:1px #ccc solid">

<span>甲状腺细胞图（Thyroid cell diagram）</span>

</div>

<div class="upload" v-if="false">

<span>上传图片（Upload pictures）</span>

</div>

<div class="picturecontent">

<img :src="origeimgUrl" alt="">

</div>

<div class="buttoncontent">

<el-upload

:auto-upload="false"

ref="buttonupload"

action=" http://192.168.100.32:8001/arithmetic/upload/file/"

:data = bolbhead

:headers = bolbhead

:show-file-list="false"

:on-success = "identifyResponse"

:on-change="imgBroadcastChange">

<div class="button buttonwhite">

<span>更换图片（Change the picture）</span>

</div>

</el-upload>

<div class="button buttonblue" style="margin-left:1vw" @click="identify">

<span>识别（Identification）</span>

</div>

</div>

</div>

<div class="bl_bottom">

<div class="small_head" style="border-bottom:1px solid #ccc">

<span>细胞分类蒙版图（Cell classification mask map）</span>

</div>

<div class="bbsh_picturecontent">

<img :src="responseimg" alt="">

</div>

<div class="bbsh_text">

<span>包含细胞核特征（Containing nuclear characteristics）：</span>

<span v-for="(item,index) in responselist" :key="index" style="color:#0b41cd">{{item}}、</span>

</div>

</div>

</div>

<div class="body_medium">

<div class="small_head">

<span>知识图谱（Knowledge graph）</span>

</div>

<div class="g6antcontent" id="mountNode"></div>

<div id="miniwindow" class="miniwindow"></div>

<div class="bm_uppunselect">

<div class="bmup_title">

<span>自动识别：</span>

<span>(绿色为自动识别内容，可以通过手动辅助新增/删除筛选项) (Green is the automatically recognized content, and manual assistance can be used to add/delete screening items)</span>

</div>

<div class="tezhengselect">

<span class="tzs_title">细胞核主要特征（The main characteristics of the cell nucleus）：</span>

<span v-for="(item,i) in bodymediumupponSelect" :key="i">

<span class="tzs_text">{{item.name}}</span>

<i v-if="item.select" class="iconfont icon-59xuanze-3" style="font-size:12px;color:#91e808;margin-right:3px"></i>

<i v-if="!item.select" class="iconfont icon-59xuanze-3" style="font-size:12px;color:white;margin-right:3px"></i>

</span>

</div>

<div class="beijingselect">

<span class="bjs_title">背景成分（Background components）：</span>

<span>

<span class="bjs_text">泡沫样细胞（Foam cells）</span>

<span class="i"></span>

</span>

</div>

<div class="bmuo_button">

<div class="button buttonblue" style="width:6vw">

<span>手动辅助筛选（Manual-assisted screening）</span>

</div>

</div>

</div>

<div class="bm_bottomselect">

<div class="bmb_select">

<el-select multiple v-model="KgReletionvalues" placeholder="关系（relationship）" size="small">

<el-option

v-for="item in KgReletionoptions"

:key="item.value"

:value="item.value">

</el-option>

</el-select>

<el-select multiple v-model="KgSematicvalues" placeholder="语义类型（Semantic type）" size="small">

<el-option

v-for="item in KgSematicoptions"

:key="item.value"

:value="item.value">

</el-option>

</el-select>

</div>

<div class="bmbo_buttoncontent">

<div class="button buttonblue" @click="findKG">

<span>高级搜索（Advanced Search）</span>

</div>

</div>

</div>

</div>

<div class="body_right">

<div class="small_head">

<span>文献来源（Source of the literature）</span>

</div>

<div class="br_selectcontent">

<div class="br_select">

<el-select multiple v-model="filerelationvalue" placeholder="关系（relationship）">

<el-option

v-for="item in filerelation"

:key="item.value"

:value="item.value">

</el-option>

</el-select>

<el-select multiple v-model="filesematicvalue" placeholder="语义类型（Semantic type）">

<el-option

v-for="item in filesematic"

:key="item.value"

:value="item.value">

</el-option>

</el-select>

<el-select multiple v-model="filenamevalue" placeholder="搜索（search）">

<el-option

v-for="item in filename"

:key="item.value"

:value="item.value">

</el-option>

</el-select>

</div>

<div class="brs_buttoncontent">

<div class="button buttonblue" @click="fileselect">

<span>筛选（Screening）</span>

</div>

</div>

</div>

<div class="filelist">

<div class="file" v-for="(item,i) in fileedgelist" :key="i">

<div class="textoppun">

<span>{{item.sourcelabel}}-{{item.label}}-{{item.targetlabel}}</span>

</div>

<div class="imgcontent">

<img src="../../static/img/2.jpg" alt="">

<div class="iconright">

<div class="i"></div>

</div>

</div>

<div class="diletitle">

<span>--《{{item.filename}}》</span>

</div>

</div>

</div>

</div>

</div>

<div class="uploadfile" v-if="false">

<el-upload

class="upload"

:auto-upload="false"

action=""

:show-file-list="false"

:on-change="imgBroadcastChange"

>

<span>上传图片（Upload pictures）</span>

</el-upload>

</div>

</div>

</template>

<script>

import G6 from "@antv/g6";

import {selectDocumentByName,getToken,identify,find} from '../utils/request'

export default {

name: 'HelloWorld',

data () {

return {

bolbhead:{responseType : "arraybuffer"},

origeimgUrl:'',

origeFile:'',

origeimgkind:[],

canvasData:{},

canvasHeight:0,

canvasWidth:0,

responseimg:'',

responselist:[],

KgReletionvalues:[],

KgReletionoptions:[{

value:"排列方式"（Arrangement method）,

label:"排列方式"（Arrangement method）

},{

value:"核型特征"（Karyotype characteristics）,

label:"核型特征"（Karyotype characteristics）

},{

value:"属于"（belong to）,

label:"属于"（belong to）

}],

KgSematicvalues:[],

KgSematicoptions:[{

value:"肿瘤细胞"（Tumor cells）

},{

value:"核沟"（Nuclear groove）

},{

value:"核内包含体"（Nuclear inclusion body）

},{

value:"核多边形"（Nuclear multilateralism）

},{

value:"核拥挤"（Nuclear congestion）

},{

value:"核增大"（Enlarged nucleus）

},{

value:"磨玻璃核"（Ground glass nucleus）

},{

value:"多核巨噬细胞"（Multinucleated macrophages）

},{

value:"核重叠"（Nuclear overlap）

},{

value:"背景细胞"（Background cells）

},{

value:"核深染"（Deep nuclear staining）

},{

value:"良性"（benign）

},],

KgChildinput:"",

value:'',

bodymediumupponSelect:[

{

name:"Tumor肿瘤细胞(核增大",（Tumor cells）

select:false,

value:'核增大'（Enlarged nucleus）

},

{

name:"核拥挤",（Nuclear congestion）

select:false,

value:'核拥挤'（Nuclear congestion）

},

{

name:"重叠",（Nuclear overlap）

select:false,

value:'核重叠'（Nuclear overlap）

},

{

name:"核多边形",（Nuclear multilateralism）

select:false,

value:'核多边形'（Nuclear multilateralism）

},

{

name:"核深染",（Deep nuclear staining）

select:false,

value:'核深染'（Deep nuclear staining）

},

{

name:"核多边形",（Nuclear multilateralism）

select:false,

value:'核多边形'（Nuclear multilateralism）

},

{

name:"）",

select:false,

value:'肿瘤细胞'（Tumor cells）

},

{

name:"磨玻璃核",（Ground glass nucleus）

select:false,

value:'磨玻璃核'（Ground glass nucleus）

},

{

name:"核沟",（Nuclear groove）

select:false,

value:'核沟'（Nuclear groove）

},

{

name:"核内包含体",（Nuclear inclusion body）

select:false,

value:'核内包含体'（Nuclear inclusion body）

}

],

edgelist:'',

fileedgelist:[],

filerelation:[],

filerelationvalue:[],

filesematic:[],

filesematicvalue:[],

filename:[],

filenamevalue:[]

}

},

mounted:function(){

this.settoken();

},

methods:{

async imgBroadcastChange (file, fileList) {

var reader = new FileReader(); //新建FileReader对象（Create a new FileReader object.）

reader.onload = (event) => {

this.origeimgUrl = event.target.result

}

reader.readAsDataURL(file.raw);

},

async settoken(){

// let data1 = { client_id:'test_client_0304',client_secret:'abcabc',grant_type:'password',scope:'read',username:'pm1',password:'Shuyu0524',roleCode:'PROJECT_MANAGER'}

// const [err1, res1] = await getToken(data1)

// localStorage.setItem('token',res1.payload.access_token)

localStorage.setItem('token','eyJhbGciOiJIUzI1NiIsInR5cCI6IkpXVCJ9.eyJhdWQiOlsidGVzdF9jbGllbnRfMDMwNCIsImt2LWF1dGgiXSwiZ3JhbnRfdHlwZSI6InBhc3N3b3JkIiwidXNlcl9uYW1lIjoicG0xIiwic2NvcGUiOlsicmVhZCJdLCJleHAiOjE2NzIxNjcwMTAsImF1dGhvcml0aWVzIjpbIlJPTEVf6aG555uu566h55CG5ZGYIl0sImp0aSI6IjA5NzZkYmI3LWVlM2YtNGRkMC1hOGQzLTg5MjBiMWRjOTVjOSIsImNsaWVudF9pZCI6InRlc3RfY2xpZW50XzAzMDQifQ.O8jNMffJ2EF6XaoQ6TZAaRztvxC7ocAbLz1cE-vRxs4')

},

async identify(){

this.$refs.buttonupload.submit();

},

fileselect(){

this.fileedgelist = this.edgelist

var dropindex = []

this.edgelist.forEach((item, index)=>{

if(this.filesematicvalue.length != 0){

var key = 0

this.filesematicvalue.forEach((semitem,semindex)=>{

if(semitem == item.sourcelabel || semitem == item.targetlabel){

key = key+1

}

})

if (key == 0){

dropindex.push(index)

}

}

if(this.filerelationvalue.length != 0){

var key = 0

this.filerelationvalue.forEach((relitem,relindex)=>{

if(relitem == item.label){

key = key+1

}

})

if (key == 0){

dropindex.push(index)

}

}

if(this.filenamevalue.length != 0){

var key = 0

this.filenamevalue.forEach((fileitem,fileindex)=>{

if(fileitem == item.filename){

key = key+1

}

})

if (key == 0){

dropindex.push(index)

}

}

})

let set = new Set(dropindex)

dropindex = [...set]

if (dropindex.length != 0){

this.fileedgelist = []

this.edgelist.forEach((item, index)=>{

var drop = 0

dropindex.forEach((dropindexitem)=>{

if(index == dropindexitem){

drop = drop + 1

}

})

if (drop == 0){

this.fileedgelist.push(item)

}

})

}else{

this.fileedgelist = this.edgelist

}

},

async identifyResponse(response,file,filelist){

this.responseimg = response.img

this.responselist = response.list

this.responselist.forEach((item,index)=>{

if(item == "膜玻璃核"){

this.bodymediumupponSelect[5].select = true

}

if(item == "肿瘤细胞（Tumor cells）"){

this.bodymediumupponSelect.forEach((item,index)=>{

if (index<5){

item.select = true

}

})

}

if(item == "核沟（Nuclear groove）"){

this.bodymediumupponSelect[6].select = true

}

})

let data = { name: response.list, programId: 329 }

const [err, res] = await selectDocumentByName(data)

var KgSelected = res

var searchDto = { projectId :329, lookAll:true}

const [err1, res1] = await find(searchDto)

var KgAllData = res1

this.Kgcanvasinit(KgSelected, KgAllData)

},

async findKG(){

var searchDto = { projectId :329, relation: this.KgReletionvalues, semantic:this.KgSematicvalues}

const [err, res] = await find(searchDto)

var KgSelected = res

var searchDto = { projectId :329, lookAll:true}

const [err1, res1] = await find(searchDto)

var KgAllData = res1

this.Kgcanvasinit(KgSelected, KgAllData)

},

removeDuplicateObj(arr){

let obj = {};

arr = arr.reduce((newArr, next) => {

obj[next.value] ? '' : (obj[next.value] = true && newArr.push(next));

return newArr;

}, []);

return arr;

},

Kgcanvasinit(KgSelected, KgAllData){

document.getElementById("miniwindow").innerHTML=''

document.getElementById("mountNode").innerHTML=''

this.edgelist = ""

this.fileedgelist = ""

this.canvasHeight = document.getElementById("mountNode").offsetHeight-4

this.canvasWidth = document.getElementById("mountNode").offsetWidth-4

var Data = KgSelected;

var node = [] ; var edge = [] ;var knownodeID = [];

Data.payload.forEach(item => {

var nodeitem = {}

nodeitem.id = item.id

nodeitem.label = item.text

nodeitem.name = item.name

knownodeID.push(item.id)

node.push(nodeitem)

if(item.from){

item.from.forEach(fromitem=>{

var edgesitem = {}

edgesitem.source = item.id

edgesitem.target = fromitem.fromId

edgesitem.sourcelabel = item.name

edgesitem.label = fromitem.relation

edgesitem.filename = item.documentName.slice(0, -4)

edge.push(edgesitem)

})

}

});

var AllData = KgAllData;

edge.forEach(item=>{

if (knownodeID.findIndex(v=>(v==item.id))==-1){

AllData.payload.forEach(allitem=>{

if(allitem.id = item.target){

var nodeitem = {}

item.targetlabel = allitem.name

nodeitem.id = allitem.id

nodeitem.label = allitem.text

nodeitem.name = allitem.name

knownodeID.push(allitem.id)

node.push(nodeitem)

}

})

}

})

node.forEach(item=>{

item.fulllabel = item.label

item.label = item.fulllabel.slice(0, 3)+"……"

})

this.edgelist = edge

this.fileedgelist = edge

edge.forEach((item,index)=>{

var sourcelabel = {value : item.sourcelabel}

var targetlabel = {value : item.targetlabel}

var filename = {value : item.filename}

var label = {value : item.label}

this.filesematic.push(sourcelabel)

this.filesematic.push(targetlabel)

this.filename.push(filename)

this.filerelation.push(label)

})

this.filesematic = this.removeDuplicateObj(this.filesematic)

this.filename = this.removeDuplicateObj(this.filename)

this.filerelation = this.removeDuplicateObj(this.filerelation)

const data ={nodes: node,edges: edge}

// 创建 G6 图实例（Create a G6 graph instance）

const minimap = new G6.Minimap({

container:"miniwindow",

size: [150, 100],

});

const tooltip = new G6.Tooltip({

offsetX: 70,

offsetY: 20,

getContent (e) {

const outDiv = document.createElement('div')

outDiv.style.width = '180px'

outDiv.innerHTML = `

<ul>

<li>语义类型（Semantic type）: ${e.item.getModel().name}</li>

<li>内容（Content）: ${e.item.getModel().fulllabel}</li>

</ul>`

return outDiv

},

itemTypes: ['node']

})

const edgetip = new G6.Tooltip({

offsetX: 70,

offsetY: 20,

getContent (e) {

const outDiv = document.createElement('div')

outDiv.style.width = '180px'

outDiv.innerHTML = `

<ul>

<li>关系（relationship）: ${e.item.getModel().label}</li>

<li>From: ${e.item.getModel().sourcelabel}</li>

<li>to: ${e.item.getModel().targetlabel}</li>

</ul>`

return outDiv

},

itemTypes: ['edge']

})

const graph = new G6.Graph({

container: 'mountNode', // 指定图画布的容器 id，与第 9 行的容器对应

// 画布宽高（Specify the container ID of the canvas, corresponding to the container in line 9.

// Canvas width and height）

width: this.canvasWidth,

height: this.canvasHeight,

layout: {

type: 'force',

linkDistance: 150 // 指定边距离为150（The specified edge distance is 150.）

},

defaultNode: {

size: 40,

},

plugins: [minimap,tooltip,edgetip]

});

graph.data({

nodes: data.nodes,

edges: data.edges.map(function (edge, i) {

edge.id = 'edge' + i;

return Object.assign({}, edge);

}),

});

graph.render();

graph.on('node:dragstart', function (e) {

graph.layout();

refreshDragedNodePosition(e);

});

graph.on('node:drag', function (e) {

const forceLayout = graph.get('layoutController').layoutMethods[0];

forceLayout.execute();

refreshDragedNodePosition(e);

});

graph.on('node:dragend', function (e) {

e.item.get('model').fx = null;

e.item.get('model').fy = null;

});

function refreshDragedNodePosition(e) {

const model = e.item.get('model');

model.fx = e.x;

model.fy = e.y;

}

}

}

}

</script>

<!-- Add "scoped" attribute to limit CSS to this component only -->

<style scoped>

div{

margin: 0;

padding: 0;

box-sizing: border-box;

}

.hello{

width: 100vw;

height: 100vh;

}

.head{

width: 100vw;

height: 6vh;

display: flex;

padding: 0 1vw;

align-items: center;

border-bottom: 1px #ccc solid;

}

.home{

width: 3vh;

height: 3vh;

text-align: center;

line-height: 3vh;

background-color: #e4eaf8;

border-radius: 5px;

margin: 0px 1vw;

}

.home>i{

color: #0b41cc;

}

.head>.title{

font-size: 15px;

color: #333;

}

.body{

width: 100vw;

height: 94vh;

display: flex

}

.body_left{

width: 30vw;

}

.bl_uppon{

height: 40vh;

border-bottom: 1px solid #ccc;

padding: 1vw;

}

.small_head{

height: 4vh;

font-size: 14px;

color: #aaa;

border-bottom: 1px solid #ccc;

line-height: 3vh;

}

.picturecontent{

display: flex;

align-items: center;

justify-content: center;

width: 28vw;

height: 24vh;

background-color: black;

margin: 2vh 0;

}

.picturecontent>img{

max-width: 100%;

max-height: 100%;

}

.buttoncontent{

display: flex;

/* align-items: flex-end; */

justify-content: flex-end;

}

.button{

font-size: 13px;

width: 5vw;

height: 4vh;

text-align: center;

line-height: 4vh;

border-radius: 5px;

}

.buttonblue{

color: white;

background-color: #0b41cd;

}

.buttonwhite{

background-color: white;

color: #333;

border: 1px #333 solid;

}

.bl_bottom{

padding: 1vw;

}

.bbsh_picturecontent{

width: 28vw;

background-color: black;

height: 37vh;

margin: 2vh 0;

display: flex;

align-items: center;

justify-content: center

}

.bbsh_picturecontent>img{

max-width: 100%;

max-height: 100%;

}

.bbsh_text{

font-size: 14px;

color: #333;

}

.body_medium{

padding: 1vw 0;

position: relative;

}

.small_head{

border: none;

padding: 0 1vw;

}

.g6antcontent{

width: 40vw;

height: 83vh;

border: 3px solid #0b41cd;

border-radius: 25px

}

.bm_uppunselect{

position: absolute;

background-color: rgba(0,0,0,0.7);

top: 6vh;

border-radius: 25px 25px 0px 0px;

width: 40vw;

padding: 15px 15px;

}

.bm_bottomselect{

width: 40vw;

position: absolute;

bottom: 5vh;

background-color: rgba(0,0,0,0.7);

border-radius: 0px 0px 25px 25px;

}

.bm_uppunselect>div{

font-size: 13px;

color: white;

margin-bottom:1vh ;

}

.bmuo_button{

margin: 0;

display: flex;

justify-content: flex-end;

}

.bmb_select{

display: flex;

}

.bmb_select>>>input{

width: 120px;

height: 5vh;

}

.bmb_select>.el-input{

margin-left: 20px;

}

.bm_bottomselect{

display: flex;

justify-content: space-between;

padding: 1vw;

}

.bmbo_buttoncontent>div{

height: 5vh;

line-height: 5vh;

width: 6vw;

}

.body_right{

padding: 1vw;

width: 30vw;

flex-direction: column;

display: flex;

}

.br_select{

display: flex;

padding: 1vw 0;

justify-content: space-around;

}

.br_selectcontent>>>input{

width: 120px;

height: 5vh;

}

.br_selectcontent{

border-bottom: 1px #ccc solid;

}

.brs_buttoncontent{

padding: 1vh 1vw;

display: flex;

justify-content: flex-end;

}

.brs_buttoncontent>div{

width: 6vw;

height: 5vh;

line-height: 5vh;

}

.filelist{

width: 28vw;

flex: 1;

overflow-x: hidden;

overflow-y: auto;

}

.file{

padding: 1vh 0;

border-bottom: 2px #0b41cd dotted;

}

.file .textoppun{

font-size: 14px;

font-weight: 600;

padding: 7px 0;

}

.file img{

width: 28vw;

}

.file .diletitle{

padding: 7px 0;

display: flex;

justify-content: flex-end

}

.uploadfile{

width: 100vw;

height: 94vh;

display: flex;

align-items: center;

justify-content: center;

}

.upload{

width: 80vw;

height: 80vh;

font-size: 68px;

line-height: 80vh;

text-align: center;

border: 5px #ccc dotted;

color: #777;

}

.miniwindow{

width: 150px;

height: 100px;

/* overflow: hidden; */

position: absolute;

bottom: 16vh;

right: 1vw;

background-color: rgba(0,0,0,0.7);

}

</style>

import numpy as np

import paddle

import paddle.nn.functional as F

import sklearn.metrics as skmetrics

def calculate_area(pred, label, num_classes, ignore_index=255):

"""

Calculate intersect, prediction and label area

Args:

pred (Tensor): The prediction by model.

label (Tensor): The ground truth of image.

num_classes (int): The unique number of target classes.

ignore_index (int): Specifies a target value that is ignored. Default: 255.

Returns:

Tensor: The intersection area of prediction and the ground on all class.

Tensor: The prediction area on all class.

Tensor: The ground truth area on all class

"""

if len(pred.shape) == 4:

pred = paddle.squeeze(pred, axis=1)

if len(label.shape) == 4:

label = paddle.squeeze(label, axis=1)

if not pred.shape == label.shape:

raise ValueError('Shape of `pred` and `label should be equal, '

'but there are {} and {}.'.format(pred.shape,

label.shape))

pred_area = []

label_area = []

intersect_area = []

mask = label != ignore_index

for i in range(num_classes):

pred_i = paddle.logical_and(pred == i, mask)

label_i = label == i

intersect_i = paddle.logical_and(pred_i, label_i)

pred_area.append(paddle.sum(paddle.cast(pred_i, "int32")))

label_area.append(paddle.sum(paddle.cast(label_i, "int32")))

intersect_area.append(paddle.sum(paddle.cast(intersect_i, "int32")))

pred_area = paddle.concat(pred_area)

label_area = paddle.concat(label_area)

intersect_area = paddle.concat(intersect_area)

return intersect_area, pred_area, label_area

def auc_roc(logits, label, num_classes, ignore_index=None):

"""

Calculate area under the roc curve

Args:

logits (Tensor): The prediction by model on testset, of shape (N,C,H,W) .

label (Tensor): The ground truth of image. (N,1,H,W)

num_classes (int): The unique number of target classes.

ignore_index (int): Specifies a target value that is ignored. Default: 255.

Returns:

auc_roc(float): The area under roc curve

"""

if ignore_index or len(np.unique(label)) > num_classes:

raise RuntimeError('labels with ignore_index is not supported yet.')

if len(label.shape) != 4:

raise ValueError(

'The shape of label is not 4 dimension as (N, C, H, W), it is {}'.

format(label.shape))

if len(logits.shape) != 4:

raise ValueError(

'The shape of logits is not 4 dimension as (N, C, H, W), it is {}'.

format(logits.shape))

N, C, H, W = logits.shape

logits = np.transpose(logits, (1, 0, 2, 3))

logits = logits.reshape([C, N * H * W]).transpose([1, 0])

label = np.transpose(label, (1, 0, 2, 3))

label = label.reshape([1, N * H * W]).squeeze()

if not logits.shape[0] == label.shape[0]:

raise ValueError('length of `logit` and `label` should be equal, '

'but they are {} and {}.'.format(logits.shape[0],

label.shape[0]))

if num_classes == 2:

auc = skmetrics.roc_auc_score(label, logits[:, 1])

else:

auc = skmetrics.roc_auc_score(label, logits, multi_class='ovr')

return auc

def mean_iou(intersect_area, pred_area, label_area):

"""

Calculate iou.

Args:

intersect_area (Tensor): The intersection area of prediction and ground truth on all classes.

pred_area (Tensor): The prediction area on all classes.

label_area (Tensor): The ground truth area on all classes.

Returns:

np.ndarray: iou on all classes.

float: mean iou of all classes.

"""

intersect_area = intersect_area.numpy()

pred_area = pred_area.numpy()

label_area = label_area.numpy()

union = pred_area + label_area - intersect_area

class_iou = []

for i in range(len(intersect_area)):

if union[i] == 0:

iou = 0

else:

iou = intersect_area[i] / union[i]

class_iou.append(iou)

miou = np.mean(class_iou)

return np.array(class_iou), miou

def dice(intersect_area, pred_area, label_area):

"""

Calculate DICE.

Args:

intersect_area (Tensor): The intersection area of prediction and ground truth on all classes.

pred_area (Tensor): The prediction area on all classes.

label_area (Tensor): The ground truth area on all classes.

Returns:

np.ndarray: DICE on all classes.

float: mean DICE of all classes.

"""

intersect_area = intersect_area.numpy()

pred_area = pred_area.numpy()

label_area = label_area.numpy()

union = pred_area + label_area

class_dice = []

for i in range(len(intersect_area)):

if union[i] == 0:

dice = 0

else:

dice = (2 * intersect_area[i]) / union[i]

class_dice.append(dice)

mdice = np.mean(class_dice)

return np.array(class_dice), mdice

\# This is a deprecated function, please use class_measurement function.

def accuracy(intersect_area, pred_area):

"""

Calculate accuracy

Args:

intersect_area (Tensor): The intersection area of prediction and ground truth on all classes..

pred_area (Tensor): The prediction area on all classes.

Returns:

np.ndarray: accuracy on all classes.

float: mean accuracy.

"""

intersect_area = intersect_area.numpy()

pred_area = pred_area.numpy()

class_acc = []

for i in range(len(intersect_area)):

if pred_area[i] == 0:

acc = 0

else:

acc = intersect_area[i] / pred_area[i]

class_acc.append(acc)

macc = np.sum(intersect_area) / np.sum(pred_area)

return np.array(class_acc), macc

def class_measurement(intersect_area, pred_area, label_area):

"""

Calculate accuracy, calss precision and class recall.

Args:

intersect_area (Tensor): The intersection area of prediction and ground truth on all classes.

pred_area (Tensor): The prediction area on all classes.

label_area (Tensor): The ground truth area on all classes.

Returns:

float: The mean accuracy.

np.ndarray: The precision of all classes.

np.ndarray: The recall of all classes.

"""

intersect_area = intersect_area.numpy()

pred_area = pred_area.numpy()

label_area = label_area.numpy()

mean_acc = np.sum(intersect_area) / np.sum(pred_area)

class_precision = []

class_recall = []

for i in range(len(intersect_area)):

precision = 0 if pred_area[i] == 0 \

else intersect_area[i] / pred_area[i]

recall = 0 if label_area[i] == 0 \

else intersect_area[i] / label_area[i]

class_precision.append(precision)

class_recall.append(recall)

return mean_acc, np.array(class_precision), np.array(class_recall)

def kappa(intersect_area, pred_area, label_area):

"""

Calculate kappa coefficient

Args:

intersect_area (Tensor): The intersection area of prediction and ground truth on all classes..

pred_area (Tensor): The prediction area on all classes.

label_area (Tensor): The ground truth area on all classes.

Returns:

float: kappa coefficient.

"""

intersect_area = intersect_area.numpy().astype(np.float64)

pred_area = pred_area.numpy().astype(np.float64)

label_area = label_area.numpy().astype(np.float64)

total_area = np.sum(label_area)

po = np.sum(intersect_area) / total_area

pe = np.sum(pred_area * label_area) / (total_area * total_area)

kappa = (po - pe) / (1 - pe)

return kappa

import contextlib

import filelock

import os

import tempfile

import numpy as np

import random

from urllib.parse import urlparse, unquote

import paddle

from paddleseg.utils import logger, seg_env

from paddleseg.utils.download import download_file_and_uncompress

@contextlib.contextmanager

def generate_tempdir(directory: str=None, **kwargs):

'''Generate a temporary directory'''

directory = seg_env.TMP_HOME if not directory else directory

with tempfile.TemporaryDirectory(dir=directory, **kwargs) as _dir:

yield _dir

def load_entire_model(model, pretrained):

if pretrained is not None:

load_pretrained_model(model, pretrained)

else:

logger.warning('Not all pretrained params of {} are loaded, ' \

'training from scratch or a pretrained backbone.'.format(model.__class__.__name__))

def download_pretrained_model(pretrained_model):

"""

Download pretrained model from url.

Args:

pretrained_model (str): the url of pretrained weight

Returns:

str: the path of pretrained weight

"""

assert urlparse(pretrained_model).netloc, "The url is not valid."

pretrained_model = unquote(pretrained_model)

savename = pretrained_model.split('/')[-1]

if not savename.endswith(('tgz', 'tar.gz', 'tar', 'zip')):

savename = pretrained_model.split('/')[-2]

else:

savename = savename.split('.')[0]

with generate_tempdir() as _dir:

with filelock.FileLock(os.path.join(seg_env.TMP_HOME, savename)):

pretrained_model = download_file_and_uncompress(

pretrained_model,

savepath=_dir,

extrapath=seg_env.PRETRAINED_MODEL_HOME,

extraname=savename)

pretrained_model = os.path.join(pretrained_model, 'model.pdparams')

return pretrained_model

def load_pretrained_model(model, pretrained_model):

if pretrained_model is not None:

logger.info('Loading pretrained model from {}'.format(pretrained_model))

if urlparse(pretrained_model).netloc:

pretrained_model = download_pretrained_model(pretrained_model)

if os.path.exists(pretrained_model):

para_state_dict = paddle.load(pretrained_model)

model_state_dict = model.state_dict()

keys = model_state_dict.keys()

num_params_loaded = 0

for k in keys:

if k not in para_state_dict:

logger.warning("{} is not in pretrained model".format(k))

elif list(para_state_dict[k].shape) != list(model_state_dict[k]

.shape):

logger.warning(

"[SKIP] Shape of pretrained params {} doesn't match.(Pretrained: {}, Actual: {})"

.format(k, para_state_dict[k].shape, model_state_dict[k]

.shape))

else:

model_state_dict[k] = para_state_dict[k]

num_params_loaded += 1

model.set_dict(model_state_dict)

logger.info("There are {}/{} variables loaded into {}.".format(

num_params_loaded,

len(model_state_dict), model.__class__.__name__))

else:

raise ValueError('The pretrained model directory is not Found: {}'.

format(pretrained_model))

else:

logger.info(

'No pretrained model to load, {} will be trained from scratch.'.

format(model.__class__.__name__))

def resume(model, optimizer, resume_model):

if resume_model is not None:

logger.info('Resume model from {}'.format(resume_model))

if os.path.exists(resume_model):

resume_model = os.path.normpath(resume_model)

ckpt_path = os.path.join(resume_model, 'model.pdparams')

para_state_dict = paddle.load(ckpt_path)

ckpt_path = os.path.join(resume_model, 'model.pdopt')

opti_state_dict = paddle.load(ckpt_path)

model.set_state_dict(para_state_dict)

optimizer.set_state_dict(opti_state_dict)

iter = resume_model.split('_')[-1]

iter = int(iter)

return iter

else:

raise ValueError(

'Directory of the model needed to resume is not Found: {}'.

format(resume_model))

else:

logger.info('No model needed to resume.')

def worker_init_fn(worker_id):

np.random.seed(random.randint(0, 100000))

def get_image_list(image_path):

"""Get image list"""

valid_suffix = [

'.JPEG', '.jpeg', '.JPG', '.jpg', '.BMP', '.bmp', '.PNG', '.png'

]

image_list = []

image_dir = None

if os.path.isfile(image_path):

if os.path.splitext(image_path)[-1] in valid_suffix:

image_list.append(image_path)

else:

image_dir = os.path.dirname(image_path)

with open(image_path, 'r') as f:

for line in f:

line = line.strip()

if len(line.split()) > 1:

line = line.split()[0]

image_list.append(os.path.join(image_dir, line))

elif os.path.isdir(image_path):

image_dir = image_path

for root, dirs, files in os.walk(image_path):

for f in files:

if '.ipynb_checkpoints' in root:

continue

if f.startswith('.'):

continue

if os.path.splitext(f)[-1] in valid_suffix:

image_list.append(os.path.join(root, f))

else:

raise FileNotFoundError(

'`--image_path` is not found. it should be a path of image, or a file list containing image paths, or a directory including images.'

)

if len(image_list) == 0:

raise RuntimeError(

'There are not image file in `--image_path`={}'.format(image_path))

return image_list, image_dir

import os

import cv2

import numpy as np

from PIL import Image as PILImage

def visualize(image, result, color_map, save_dir=None, weight=0.6):

"""

Convert predict result to color image, and save added image.

Args:

image (str): The path of origin image.

result (np.ndarray): The predict result of image.

color_map (list): The color used to save the prediction results.

save_dir (str): The directory for saving visual image. Default: None.

weight (float): The image weight of visual image, and the result weight is (1 - weight). Default: 0.6

Returns:

vis_result (np.ndarray): If `save_dir` is None, return the visualized result.

"""

color_map = [color_map[i:i + 3] for i in range(0, len(color_map), 3)]

color_map = np.array(color_map).astype("uint8")

\# Use OpenCV LUT for color mapping

c1 = cv2.LUT(result, color_map[:, 0])

c2 = cv2.LUT(result, color_map[:, 1])

c3 = cv2.LUT(result, color_map[:, 2])

pseudo_img = np.dstack((c3, c2, c1))

im = cv2.imread(image)

vis_result = cv2.addWeighted(im, weight, pseudo_img, 1 - weight, 0)

if save_dir is not None:

if not os.path.exists(save_dir):

os.makedirs(save_dir)

image_name = os.path.split(image)[-1]

out_path = os.path.join(save_dir, image_name)

cv2.imwrite(out_path, vis_result)

else:

return vis_result

def get_pseudo_color_map(pred, color_map=None):

"""

Get the pseudo color image.

Args:

pred (numpy.ndarray): the origin predicted image.

color_map (list, optional): the palette color map. Default: None,

use paddleseg's default color map.

Returns:

(numpy.ndarray): the pseduo image.

"""

pred_mask = PILImage.fromarray(pred.astype(np.uint8), mode='P')

if color_map is None:

color_map = get_color_map_list(256)

pred_mask.putpalette(color_map)

return pred_mask

def get_color_map_list(num_classes, custom_color=None):

"""

Returns the color map for visualizing the segmentation mask,

which can support arbitrary number of classes.

Args:

num_classes (int): Number of classes.

custom_color (list, optional): Save images with a custom color map. Default: None, use paddleseg's default color map.

Returns:

(list). The color map.

"""

num_classes += 1

color_map = num_classes * [0, 0, 0]

for i in range(0, num_classes):

j = 0

lab = i

while lab:

color_map[i * 3] |= (((lab >> 0) & 1) << (7 - j))

color_map[i * 3 + 1] |= (((lab >> 1) & 1) << (7 - j))

color_map[i * 3 + 2] |= (((lab >> 2) & 1) << (7 - j))

j += 1

lab >>= 3

color_map = color_map[3:]

if custom_color:

color_map[:len(custom_color)] = custom_color

return color_map

def paste_images(image_list):

"""

Paste all image to a image.

Args:

image_list (List or Tuple): The images to be pasted and their size are the same.

Returns:

result_img (PIL.Image): The pasted image.

"""

assert isinstance(image_list,

(list, tuple)), "image_list should be a list or tuple"

assert len(

image_list) > 1, "The length of image_list should be greater than 1"

pil_img_list = []

for img in image_list:

if isinstance(img, str):

assert os.path.exists(img), "The image is not existed: {}".format(

img)

img = PILImage.open(img)

img = np.array(img)

elif isinstance(img, np.ndarray):

img = PILImage.fromarray(img)

pil_img_list.append(img)

sample_img = pil_img_list[0]

size = sample_img.size

for img in pil_img_list:

assert size == img.size, "The image size in image_list should be the same"

width, height = sample_img.size

result_img = PILImage.new(sample_img.mode,

(width * len(pil_img_list), height))

for i, img in enumerate(pil_img_list):

result_img.paste(img, box=(width * i, 0))

return result_img

import random

import math

import cv2

import numpy as np

from PIL import Image

from paddleseg.cvlibs import manager

from paddleseg.transforms import functional

@manager.TRANSFORMS.add_component

class Compose:

"""

Do transformation on input data with corresponding pre-processing and augmentation operations.

The shape of input data to all operations is [height, width, channels].

Args:

transforms (list): A list contains data pre-processing or augmentation. Empty list means only reading images, no transformation.

to_rgb (bool, optional): If converting image to RGB color space. Default: True.

Raises:

TypeError: When 'transforms' is not a list.

ValueError: when the length of 'transforms' is less than 1.

"""

def __init__(self, transforms, to_rgb=True):

if not isinstance(transforms, list):

raise TypeError('The transforms must be a list!')

self.transforms = transforms

self.to_rgb = to_rgb

def __call__(self, data):

"""

Args:

data: A dict to deal with. It may include keys: 'img', 'label', 'trans_info' and 'gt_fields'.

'trans_info' reserve the image shape informating. And the 'gt_fields' save the key need to transforms

together with 'img'

Returns: A dict after process。

"""

if 'img' not in data.keys():

raise ValueError("`data` must include `img` key.")

if isinstance(data['img'], str):

data['img'] = cv2.imread(data['img']).astype('float32')

if data['img'] is None:

raise ValueError('Can\'t read The image file {}!'.format(data[

'img']))

if not isinstance(data['img'], np.ndarray):

raise TypeError("Image type is not numpy.")

if len(data['img'].shape) != 3:

raise ValueError('Image is not 3-dimensional.')

if 'label' in data.keys() and isinstance(data['label'], str):

data['label'] = np.asarray(Image.open(data['label']))

if self.to_rgb:

data['img'] = cv2.cvtColor(data['img'], cv2.COLOR_BGR2RGB)

\# the `trans_info` will save the process of image shape, and will be used in evaluation and prediction.

if 'trans_info' not in data.keys():

data['trans_info'] = []

for op in self.transforms:

data = op(data)

data['img'] = np.transpose(data['img'], (2, 0, 1))

return data

@manager.TRANSFORMS.add_component

class RandomHorizontalFlip:

"""

Flip an image horizontally with a certain probability.

Args:

prob (float, optional): A probability of horizontally flipping. Default: 0.5.

"""

def __init__(self, prob=0.5):

self.prob = prob

def __call__(self, data):

if random.random() < self.prob:

data['img'] = functional.horizontal_flip(data['img'])

for key in data.get('gt_fields', []):

data[key] = functional.horizontal_flip(data[key])

return data

@manager.TRANSFORMS.add_component

class RandomVerticalFlip:

"""

Flip an image vertically with a certain probability.

Args:

prob (float, optional): A probability of vertical flipping. Default: 0.1.

"""

def __init__(self, prob=0.1):

self.prob = prob

def __call__(self, data):

if random.random() < self.prob:

data['img'] = functional.vertical_flip(data['img'])

for key in data.get('gt_fields', []):

data[key] = functional.vertical_flip(data[key])

return data

@manager.TRANSFORMS.add_component

class Resize:

"""

Resize an image.

Args:

target_size (list|tuple, optional): The target size of image. Default: (512, 512).

interp (str, optional): The interpolation mode of resize is consistent with opencv.

['NEAREST', 'LINEAR', 'CUBIC', 'AREA', 'LANCZOS4', 'RANDOM']. Note that when it is

'RANDOM', a random interpolation mode would be specified. Default: "LINEAR".

Raises:

TypeError: When 'target_size' type is neither list nor tuple.

ValueError: When "interp" is out of pre-defined methods ('NEAREST', 'LINEAR', 'CUBIC',

'AREA', 'LANCZOS4', 'RANDOM').

"""

\# The interpolation mode

interp_dict = {

'NEAREST': cv2.INTER_NEAREST,

'LINEAR': cv2.INTER_LINEAR,

'CUBIC': cv2.INTER_CUBIC,

'AREA': cv2.INTER_AREA,

'LANCZOS4': cv2.INTER_LANCZOS4

}

def __init__(self, target_size=(512, 512), interp='LINEAR'):

self.interp = interp

if not (interp == "RANDOM" or interp in self.interp_dict):

raise ValueError("`interp` should be one of {}".format(

self.interp_dict.keys()))

if isinstance(target_size, list) or isinstance(target_size, tuple):

if len(target_size) != 2:

raise ValueError(

'`target_size` should include 2 elements, but it is {}'.

format(target_size))

else:

raise TypeError(

"Type of `target_size` is invalid. It should be list or tuple, but it is {}"

.format(type(target_size)))

self.target_size = target_size

def __call__(self, data):

data['trans_info'].append(('resize', data['img'].shape[0:2]))

if self.interp == "RANDOM":

interp = random.choice(list(self.interp_dict.keys()))

else:

interp = self.interp

data['img'] = functional.resize(data['img'], self.target_size,

self.interp_dict[interp])

for key in data.get('gt_fields', []):

data[key] = functional.resize(data[key], self.target_size,

cv2.INTER_NEAREST)

return data

@manager.TRANSFORMS.add_component

class ResizeByLong:

"""

Resize the long side of an image to given size, and then scale the other side proportionally.

Args:

long_size (int): The target size of long side.

"""

def __init__(self, long_size):

self.long_size = long_size

def __call__(self, data):

data['trans_info'].append(('resize', data['img'].shape[0:2]))

data['img'] = functional.resize_long(data['img'], self.long_size)

for key in data.get('gt_fields', []):

data[key] = functional.resize_long(data[key], self.long_size,

cv2.INTER_NEAREST)

return data

@manager.TRANSFORMS.add_component

class ResizeByShort:

"""

Resize the short side of an image to given size, and then scale the other side proportionally.

Args:

short_size (int): The target size of short side.

"""

def __init__(self, short_size):

self.short_size = short_size

def __call__(self, data):

data['trans_info'].append(('resize', data['img'].shape[0:2]))

data['img'] = functional.resize_short(data['img'], self.short_size)

for key in data.get('gt_fields', []):

data[key] = functional.resize_short(data[key], self.short_size,

cv2.INTER_NEAREST)

return data

@manager.TRANSFORMS.add_component

class LimitLong:

"""

Limit the long edge of image.

If the long edge is larger than max_long, resize the long edge

to max_long, while scale the short edge proportionally.

If the long edge is smaller than min_long, resize the long edge

to min_long, while scale the short edge proportionally.

Args:

max_long (int, optional): If the long edge of image is larger than max_long,

it will be resize to max_long. Default: None.

min_long (int, optional): If the long edge of image is smaller than min_long,

it will be resize to min_long. Default: None.

"""

def __init__(self, max_long=None, min_long=None):

if max_long is not None:

if not isinstance(max_long, int):

raise TypeError(

"Type of `max_long` is invalid. It should be int, but it is {}"

.format(type(max_long)))

if min_long is not None:

if not isinstance(min_long, int):

raise TypeError(

"Type of `min_long` is invalid. It should be int, but it is {}"

.format(type(min_long)))

if (max_long is not None) and (min_long is not None):

if min_long > max_long:

raise ValueError(

'`max_long should not smaller than min_long, but they are {} and {}'

.format(max_long, min_long))

self.max_long = max_long

self.min_long = min_long

def __call__(self, data):

data['trans_info'].append(('resize', data['img'].shape[0:2]))

h, w = data['img'].shape[0], data['img'].shape[1]

long_edge = max(h, w)

target = long_edge

if (self.max_long is not None) and (long_edge > self.max_long):

target = self.max_long

elif (self.min_long is not None) and (long_edge < self.min_long):

target = self.min_long

if target != long_edge:

data['img'] = functional.resize_long(data['img'], target)

for key in data.get('gt_fields', []):

data[key] = functional.resize_long(data[key], target,

cv2.INTER_NEAREST)

return data

@manager.TRANSFORMS.add_component

class ResizeRangeScaling:

"""

Resize the long side of an image into a range, and then scale the other side proportionally.

Args:

min_value (int, optional): The minimum value of long side after resize. Default: 400.

max_value (int, optional): The maximum value of long side after resize. Default: 600.

"""

def __init__(self, min_value=400, max_value=600):

if min_value > max_value:

raise ValueError('min_value must be less than max_value, '

'but they are {} and {}.'.format(min_value,

max_value))

self.min_value = min_value

self.max_value = max_value

def __call__(self, data):

if self.min_value == self.max_value:

random_size = self.max_value

else:

random_size = int(

np.random.uniform(self.min_value, self.max_value) + 0.5)

data['img'] = functional.resize_long(data['img'], random_size,

cv2.INTER_LINEAR)

for key in data.get('gt_fields', []):

data[key] = functional.resize_long(data[key], random_size,

cv2.INTER_NEAREST)

return data

@manager.TRANSFORMS.add_component

class ResizeStepScaling:

"""

Scale an image proportionally within a range.

Args:

min_scale_factor (float, optional): The minimum scale. Default: 0.75.

max_scale_factor (float, optional): The maximum scale. Default: 1.25.

scale_step_size (float, optional): The scale interval. Default: 0.25.

Raises:

ValueError: When min_scale_factor is smaller than max_scale_factor.

"""

def __init__(self,

min_scale_factor=0.75,

max_scale_factor=1.25,

scale_step_size=0.25):

if min_scale_factor > max_scale_factor:

raise ValueError(

'min_scale_factor must be less than max_scale_factor, '

'but they are {} and {}.'.format(min_scale_factor,

max_scale_factor))

self.min_scale_factor = min_scale_factor

self.max_scale_factor = max_scale_factor

self.scale_step_size = scale_step_size

def __call__(self, data):

if self.min_scale_factor == self.max_scale_factor:

scale_factor = self.min_scale_factor

elif self.scale_step_size == 0:

scale_factor = np.random.uniform(self.min_scale_factor,

self.max_scale_factor)

else:

num_steps = int((self.max_scale_factor - self.min_scale_factor) /

self.scale_step_size + 1)

scale_factors = np.linspace(self.min_scale_factor,

self.max_scale_factor,

num_steps).tolist()

np.random.shuffle(scale_factors)

scale_factor = scale_factors[0]

w = int(round(scale_factor * data['img'].shape[1]))

h = int(round(scale_factor * data['img'].shape[0]))

data['img'] = functional.resize(data['img'], (w, h), cv2.INTER_LINEAR)

for key in data.get('gt_fields', []):

data[key] = functional.resize(data[key], (w, h), cv2.INTER_NEAREST)

return data

@manager.TRANSFORMS.add_component

class Normalize:

"""

Normalize an image.

Args:

mean (list, optional): The mean value of a data set. Default: [0.5, 0.5, 0.5].

std (list, optional): The standard deviation of a data set. Default: [0.5, 0.5, 0.5].

Raises:

ValueError: When mean/std is not list or any value in std is 0.

"""

def __init__(self, mean=(0.5, 0.5, 0.5), std=(0.5, 0.5, 0.5)):

self.mean = mean

self.std = std

if not (isinstance(self.mean,

(list, tuple)) and isinstance(self.std,

(list, tuple))):

raise ValueError(

"{}: input type is invalid. It should be list or tuple".format(

self))

from functools import reduce

if reduce(lambda x, y: x * y, self.std) == 0:

raise ValueError('{}: std is invalid!'.format(self))

def __call__(self, data):

mean = np.array(self.mean)[np.newaxis, np.newaxis, :]

std = np.array(self.std)[np.newaxis, np.newaxis, :]

data['img'] = functional.normalize(data['img'], mean, std)

return data

@manager.TRANSFORMS.add_component

class Padding:

"""

Add bottom-right padding to a raw image or annotation image.

Args:

target_size (list|tuple): The target size after padding.

im_padding_value (list, optional): The padding value of raw image.

Default: [127.5, 127.5, 127.5].

label_padding_value (int, optional): The padding value of annotation image. Default: 255.

Raises:

TypeError: When target_size is neither list nor tuple.

ValueError: When the length of target_size is not 2.

"""

def __init__(self,

target_size,

im_padding_value=(127.5, 127.5, 127.5),

label_padding_value=255):

if isinstance(target_size, list) or isinstance(target_size, tuple):

if len(target_size) != 2:

raise ValueError(

'`target_size` should include 2 elements, but it is {}'.

format(target_size))

else:

raise TypeError(

"Type of target_size is invalid. It should be list or tuple, now is {}"

.format(type(target_size)))

self.target_size = target_size

self.im_padding_value = im_padding_value

self.label_padding_value = label_padding_value

def __call__(self, data):

data['trans_info'].append(('padding', data['img'].shape[0:2]))

im_height, im_width = data['img'].shape[0], data['img'].shape[1]

if isinstance(self.target_size, int):

target_height = self.target_size

target_width = self.target_size

else:

target_height = self.target_size[1]

target_width = self.target_size[0]

pad_height = target_height - im_height

pad_width = target_width - im_width

if pad_height < 0 or pad_width < 0:

raise ValueError(

'The size of image should be less than `target_size`, but the size of image ({}, {}) is larger than `target_size` ({}, {})'

.format(im_width, im_height, target_width, target_height))

else:

data['img'] = cv2.copyMakeBorder(

data['img'],

0,

pad_height,

0,

pad_width,

cv2.BORDER_CONSTANT,

value=self.im_padding_value)

for key in data.get('gt_fields', []):

data[key] = cv2.copyMakeBorder(

data[key],

0,

pad_height,

0,

pad_width,

cv2.BORDER_CONSTANT,

value=self.label_padding_value)

return data

@manager.TRANSFORMS.add_component

class PaddingByAspectRatio:

"""

Args:

aspect_ratio (int|float, optional): The aspect ratio = width / height. Default: 1.

"""

def __init__(self,

aspect_ratio=1,

im_padding_value=(127.5, 127.5, 127.5),

label_padding_value=255):

self.aspect_ratio = aspect_ratio

self.im_padding_value = im_padding_value

self.label_padding_value = label_padding_value

def __call__(self, data):

img_height = data['img'].shape[0]

img_width = data['img'].shape[1]

ratio = img_width / img_height

if ratio == self.aspect_ratio:

return data

elif ratio > self.aspect_ratio:

img_height = int(img_width / self.aspect_ratio)

else:

img_width = int(img_height * self.aspect_ratio)

padding = Padding(

(img_width, img_height),

im_padding_value=self.im_padding_value,

label_padding_value=self.label_padding_value)

return padding(data)

@manager.TRANSFORMS.add_component

class RandomPaddingCrop:

"""

Crop a sub-image from a raw image and annotation image randomly. If the target cropping size

is larger than original image, then the bottom-right padding will be added.

Args:

crop_size (tuple, optional): The target cropping size. Default: (512, 512).

im_padding_value (list, optional): The padding value of raw image.

Default: [127.5, 127.5, 127.5].

label_padding_value (int, optional): The padding value of annotation image. Default: 255.

Raises:

TypeError: When crop_size is neither list nor tuple.

ValueError: When the length of crop_size is not 2.

"""

def __init__(self,

crop_size=(512, 512),

im_padding_value=(127.5, 127.5, 127.5),

label_padding_value=255):

if isinstance(crop_size, list) or isinstance(crop_size, tuple):

if len(crop_size) != 2:

raise ValueError(

'Type of `crop_size` is list or tuple. It should include 2 elements, but it is {}'

.format(crop_size))

else:

raise TypeError(

"The type of `crop_size` is invalid. It should be list or tuple, but it is {}"

.format(type(crop_size)))

self.crop_size = crop_size

self.im_padding_value = im_padding_value

self.label_padding_value = label_padding_value

def __call__(self, data):

if isinstance(self.crop_size, int):

crop_width = self.crop_size

crop_height = self.crop_size

else:

crop_width = self.crop_size[0]

crop_height = self.crop_size[1]

img_height = data['img'].shape[0]

img_width = data['img'].shape[1]

if img_height == crop_height and img_width == crop_width:

return data

else:

pad_height = max(crop_height - img_height, 0)

pad_width = max(crop_width - img_width, 0)

if (pad_height > 0 or pad_width > 0):

data['img'] = cv2.copyMakeBorder(

data['img'],

0,

pad_height,

0,

pad_width,

cv2.BORDER_CONSTANT,

value=self.im_padding_value)

for key in data.get('gt_fields', []):

data[key] = cv2.copyMakeBorder(

data[key],

0,

pad_height,

0,

pad_width,

cv2.BORDER_CONSTANT,

value=self.label_padding_value)

img_height = data['img'].shape[0]

img_width = data['img'].shape[1]

if crop_height > 0 and crop_width > 0:

h_off = np.random.randint(img_height - crop_height + 1)

w_off = np.random.randint(img_width - crop_width + 1)

data['img'] = data['img'][h_off:(crop_height + h_off), w_off:(

w_off + crop_width), :]

for key in data.get('gt_fields', []):

data[key] = data[key][h_off:(crop_height + h_off), w_off:(

w_off + crop_width)]

return data

@manager.TRANSFORMS.add_component

class RandomCenterCrop:

"""

Crops the given the input data at the center.

Args:

retain_ratio (tuple or list, optional): The length of the input list or tuple must be 2. Default: (0.5, 0.5).

the first value is used for width and the second is for height.

In addition, the minimum size of the cropped image is [width * retain_ratio[0], height * retain_ratio[1]].

Raises:

TypeError: When retain_ratio is neither list nor tuple. Default: None.

ValueError: When the value of retain_ratio is not in [0-1].

"""

def __init__(self, retain_ratio=(0.5, 0.5)):

if isinstance(retain_ratio, list) or isinstance(retain_ratio, tuple):

if len(retain_ratio) != 2:

raise ValueError(

'When type of `retain_ratio` is list or tuple, it shoule include 2 elements, but it is {}'

.format(retain_ratio))

if retain_ratio[0] > 1 or retain_ratio[1] > 1 or retain_ratio[

0] < 0 or retain_ratio[1] < 0:

raise ValueError(

'Value of `retain_ratio` should be in [0, 1], but it is {}'.

format(retain_ratio))

else:

raise TypeError(

"The type of `retain_ratio` is invalid. It should be list or tuple, but it is {}"

.format(type(retain_ratio)))

self.retain_ratio = retain_ratio

def __call__(self, data):

retain_width = self.retain_ratio[0]

retain_height = self.retain_ratio[1]

img_height = data['img'].shape[0]

img_width = data['img'].shape[1]

if retain_width == 1. and retain_height == 1.:

return data

else:

randw = np.random.randint(img_width * (1 - retain_width))

randh = np.random.randint(img_height * (1 - retain_height))

offsetw = 0 if randw == 0 else np.random.randint(randw)

offseth = 0 if randh == 0 else np.random.randint(randh)

p0, p1, p2, p3 = offseth, img_height + offseth - randh, offsetw, img_width + offsetw - randw

data['img'] = data['img'][p0:p1, p2:p3, :]

for key in data.get('gt_fields', []):

data[key] = data[key][p0:p1, p2:p3]

return data

@manager.TRANSFORMS.add_component

class ScalePadding:

"""

Add center padding to a raw image or annotation image,then scale the

image to target size.

Args:

target_size (list|tuple, optional): The target size of image. Default: (512, 512).

im_padding_value (list, optional): The padding value of raw image.

Default: [127.5, 127.5, 127.5].

label_padding_value (int, optional): The padding value of annotation image. Default: 255.

Raises:

TypeError: When target_size is neither list nor tuple.

ValueError: When the length of target_size is not 2.

"""

def __init__(self,

target_size=(512, 512),

im_padding_value=(127.5, 127.5, 127.5),

label_padding_value=255):

if isinstance(target_size, list) or isinstance(target_size, tuple):

if len(target_size) != 2:

raise ValueError(

'`target_size` should include 2 elements, but it is {}'.

format(target_size))

else:

raise TypeError(

"Type of `target_size` is invalid. It should be list or tuple, but it is {}"

.format(type(target_size)))

self.target_size = target_size

self.im_padding_value = im_padding_value

self.label_padding_value = label_padding_value

def __call__(self, data):

height = data['img'].shape[0]

width = data['img'].shape[1]

new_im = np.zeros(

(max(height, width), max(height, width), 3)) + self.im_padding_value

if 'label' in data['gt_fields']:

new_label = np.zeros((max(height, width), max(height, width)

)) + self.label_padding_value

if height > width:

padding = int((height - width) / 2)

new_im[:, padding:padding + width, :] = data['img']

if 'label' in data['gt_fields']:

new_label[:, padding:padding + width] = data['label']

else:

padding = int((width - height) / 2)

new_im[padding:padding + height, :, :] = data['img']

if 'label' in data['gt_fields']:

new_label[padding:padding + height, :] = data['label']

data['img'] = np.uint8(new_im)

data['img'] = functional.resize(

data['img'], self.target_size, interp=cv2.INTER_CUBIC)

if 'label' in data['gt_fields']:

data['label'] = np.uint8(new_label)

data['label'] = functional.resize(

data['label'], self.target_size, interp=cv2.INTER_CUBIC)

return data

@manager.TRANSFORMS.add_component

class RandomNoise:

"""

Superimposing noise on an image with a certain probability.

Args:

prob (float, optional): A probability of blurring an image. Default: 0.5.

max_sigma(float, optional): The maximum value of standard deviation of the distribution.

Default: 10.0.

"""

def __init__(self, prob=0.5, max_sigma=10.0):

self.prob = prob

self.max_sigma = max_sigma

def __call__(self, data):

if random.random() < self.prob:

mu = 0

sigma = random.random() * self.max_sigma

data['img'] = np.array(data['img'], dtype=np.float32)

data['img'] += np.random.normal(mu, sigma, data['img'].shape)

data['img'][data['img'] > 255] = 255

data['img'][data['img'] < 0] = 0

return data

@manager.TRANSFORMS.add_component

class RandomBlur:

"""

Blurring an image by a Gaussian function with a certain probability.

Args:

prob (float, optional): A probability of blurring an image. Default: 0.1.

blur_type(str, optional): A type of blurring an image,

gaussian stands for cv2.GaussianBlur,

median stands for cv2.medianBlur,

blur stands for cv2.blur,

random represents randomly selected from above.

Default: gaussian.

"""

def __init__(self, prob=0.1, blur_type="gaussian"):

self.prob = prob

self.blur_type = blur_type

def __call__(self, data):

if self.prob <= 0:

n = 0

elif self.prob >= 1:

n = 1

else:

n = int(1.0 / self.prob)

if n > 0:

if np.random.randint(0, n) == 0:

radius = np.random.randint(3, 10)

if radius % 2 != 1:

radius = radius + 1

if radius > 9:

radius = 9

data['img'] = np.array(data['img'], dtype='uint8')

if self.blur_type == "gaussian":

data['img'] = cv2.GaussianBlur(data['img'],

(radius, radius), 0, 0)

elif self.blur_type == "median":

data['img'] = cv2.medianBlur(data['img'], radius)

elif self.blur_type == "blur":

data['img'] = cv2.blur(data['img'], (radius, radius))

elif self.blur_type == "random":

select = random.random()

if select < 0.3:

data['img'] = cv2.GaussianBlur(data['img'],

(radius, radius), 0)

elif select < 0.6:

data['img'] = cv2.medianBlur(data['img'], radius)

else:

data['img'] = cv2.blur(data['img'], (radius, radius))

else:

data['img'] = cv2.GaussianBlur(data['img'],

(radius, radius), 0, 0)

data['img'] = np.array(data['img'], dtype='float32')

return data

@manager.TRANSFORMS.add_component

class RandomRotation:

"""

Rotate an image randomly with padding.

Args:

max_rotation (float, optional): The maximum rotation degree. Default: 15.

im_padding_value (list, optional): The padding value of raw image.

Default: [127.5, 127.5, 127.5].

label_padding_value (int, optional): The padding value of annotation image. Default: 255.

"""

def __init__(self,

max_rotation=15,

im_padding_value=(127.5, 127.5, 127.5),

label_padding_value=255):

self.max_rotation = max_rotation

self.im_padding_value = im_padding_value

self.label_padding_value = label_padding_value

def __call__(self, data):

if self.max_rotation > 0:

(h, w) = data['img'].shape[:2]

do_rotation = np.random.uniform(-self.max_rotation,

self.max_rotation)

pc = (w // 2, h // 2)

r = cv2.getRotationMatrix2D(pc, do_rotation, 1.0)

cos = np.abs(r[0, 0])

sin = np.abs(r[0, 1])

nw = int((h * sin) + (w * cos))

nh = int((h * cos) + (w * sin))

(cx, cy) = pc

r[0, 2] += (nw / 2) - cx

r[1, 2] += (nh / 2) - cy

dsize = (nw, nh)

data['img'] = cv2.warpAffine(

data['img'],

r,

dsize=dsize,

flags=cv2.INTER_LINEAR,

borderMode=cv2.BORDER_CONSTANT,

borderValue=self.im_padding_value)

for key in data.get('gt_fields', []):

data[key] = cv2.warpAffine(

data[key],

r,

dsize=dsize,

flags=cv2.INTER_NEAREST,

borderMode=cv2.BORDER_CONSTANT,

borderValue=self.label_padding_value)

return data

@manager.TRANSFORMS.add_component

class RandomScaleAspect:

"""

Crop a sub-image from an original image with a range of area ratio and aspect and

then scale the sub-image back to the size of the original image.

Args:

min_scale (float, optional): The minimum area ratio of cropped image to the original image. Default: 0.5.

aspect_ratio (float, optional): The minimum aspect ratio. Default: 0.33.

"""

def __init__(self, min_scale=0.5, aspect_ratio=0.33):

self.min_scale = min_scale

self.aspect_ratio = aspect_ratio

def __call__(self, data):

if self.min_scale != 0 and self.aspect_ratio != 0:

img_height = data['img'].shape[0]

img_width = data['img'].shape[1]

for i in range(0, 10):

area = img_height * img_width

target_area = area * np.random.uniform(self.min_scale, 1.0)

aspectRatio = np.random.uniform(self.aspect_ratio,

1.0 / self.aspect_ratio)

dw = int(np.sqrt(target_area * 1.0 * aspectRatio))

dh = int(np.sqrt(target_area * 1.0 / aspectRatio))

if (np.random.randint(10) < 5):

tmp = dw

dw = dh

dh = tmp

if (dh < img_height and dw < img_width):

h1 = np.random.randint(0, img_height - dh)

w1 = np.random.randint(0, img_width - dw)

data['img'] = data['img'][h1:(h1 + dh), w1:(w1 + dw), :]

data['img'] = cv2.resize(

data['img'], (img_width, img_height),

interpolation=cv2.INTER_LINEAR)

for key in data.get('gt_fields', []):

data[key] = data[key][h1:(h1 + dh), w1:(w1 + dw)]

data[key] = cv2.resize(

data[key], (img_width, img_height),

interpolation=cv2.INTER_NEAREST)

break

return data

@manager.TRANSFORMS.add_component

class RandomDistort:

"""

Distort an image with random configurations.

Args:

brightness_range (float, optional): A range of brightness. Default: 0.5.

brightness_prob (float, optional): A probability of adjusting brightness. Default: 0.5.

contrast_range (float, optional): A range of contrast. Default: 0.5.

contrast_prob (float, optional): A probability of adjusting contrast. Default: 0.5.

saturation_range (float, optional): A range of saturation. Default: 0.5.

saturation_prob (float, optional): A probability of adjusting saturation. Default: 0.5.

hue_range (int, optional): A range of hue. Default: 18.

hue_prob (float, optional): A probability of adjusting hue. Default: 0.5.

sharpness_range (float, optional): A range of sharpness. Default: 0.5.

sharpness_prob (float, optional): A probability of adjusting saturation. Default: 0.

"""

def __init__(self,

brightness_range=0.5,

brightness_prob=0.5,

contrast_range=0.5,

contrast_prob=0.5,

saturation_range=0.5,

saturation_prob=0.5,

hue_range=18,

hue_prob=0.5,

sharpness_range=0.5,

sharpness_prob=0):

self.brightness_range = brightness_range

self.brightness_prob = brightness_prob

self.contrast_range = contrast_range

self.contrast_prob = contrast_prob

self.saturation_range = saturation_range

self.saturation_prob = saturation_prob

self.hue_range = hue_range

self.hue_prob = hue_prob

self.sharpness_range = sharpness_range

self.sharpness_prob = sharpness_prob

def __call__(self, data):

brightness_lower = 1 - self.brightness_range

brightness_upper = 1 + self.brightness_range

contrast_lower = 1 - self.contrast_range

contrast_upper = 1 + self.contrast_range

saturation_lower = 1 - self.saturation_range

saturation_upper = 1 + self.saturation_range

hue_lower = -self.hue_range

hue_upper = self.hue_range

sharpness_lower = 1 - self.sharpness_range

sharpness_upper = 1 + self.sharpness_range

ops = [

functional.brightness, functional.contrast, functional.saturation,

functional.hue, functional.sharpness

]

random.shuffle(ops)

params_dict = {

'brightness': {

'brightness_lower': brightness_lower,

'brightness_upper': brightness_upper

},

'contrast': {

'contrast_lower': contrast_lower,

'contrast_upper': contrast_upper

},

'saturation': {

'saturation_lower': saturation_lower,

'saturation_upper': saturation_upper

},

'hue': {

'hue_lower': hue_lower,

'hue_upper': hue_upper

},

'sharpness': {

'sharpness_lower': sharpness_lower,

'sharpness_upper': sharpness_upper,

}

}

prob_dict = {

'brightness': self.brightness_prob,

'contrast': self.contrast_prob,

'saturation': self.saturation_prob,

'hue': self.hue_prob,

'sharpness': self.sharpness_prob

}

data['img'] = data['img'].astype('uint8')

data['img'] = Image.fromarray(data['img'])

for id in range(len(ops)):

params = params_dict[ops[id].__name__]

prob = prob_dict[ops[id].__name__]

params['im'] = data['img']

if np.random.uniform(0, 1) < prob:

data['img'] = ops[id](**params)

data['img'] = np.asarray(data['img']).astype('float32')

return data

@manager.TRANSFORMS.add_component

class RandomAffine:

"""

Affine transform an image with random configurations.

Args:

size (tuple, optional): The target size after affine transformation. Default: (224, 224).

translation_offset (float, optional): The maximum translation offset. Default: 0.

max_rotation (float, optional): The maximum rotation degree. Default: 15.

min_scale_factor (float, optional): The minimum scale. Default: 0.75.

max_scale_factor (float, optional): The maximum scale. Default: 1.25.

im_padding_value (float, optional): The padding value of raw image. Default: (128, 128, 128).

label_padding_value (int, optional): The padding value of annotation image. Default: (255, 255, 255).

"""

def __init__(self,

size=(224, 224),

translation_offset=0,

max_rotation=15,

min_scale_factor=0.75,

max_scale_factor=1.25,

im_padding_value=(128, 128, 128),

label_padding_value=255):

self.size = size

self.translation_offset = translation_offset

self.max_rotation = max_rotation

self.min_scale_factor = min_scale_factor

self.max_scale_factor = max_scale_factor

self.im_padding_value = im_padding_value

self.label_padding_value = label_padding_value

def __call__(self, data):

w, h = self.size

bbox = [0, 0, data['img'].shape[1] - 1, data['img'].shape[0] - 1]

x_offset = (random.random() - 0.5) * 2 * self.translation_offset

y_offset = (random.random() - 0.5) * 2 * self.translation_offset

dx = (w - (bbox[2] + bbox[0])) / 2.0

dy = (h - (bbox[3] + bbox[1])) / 2.0

matrix_trans = np.array([[1.0, 0, dx], [0, 1.0, dy], [0, 0, 1.0]])

angle = random.random() * 2 * self.max_rotation - self.max_rotation

scale = random.random() * (self.max_scale_factor - self.min_scale_factor

) + self.min_scale_factor

scale *= np.mean(

[float(w) / (bbox[2] - bbox[0]), float(h) / (bbox[3] - bbox[1])])

alpha = scale * math.cos(angle / 180.0 * math.pi)

beta = scale * math.sin(angle / 180.0 * math.pi)

centerx = w / 2.0 + x_offset

centery = h / 2.0 + y_offset

matrix = np.array(

[[alpha, beta, (1 - alpha) * centerx - beta * centery],

[-beta, alpha, beta * centerx + (1 - alpha) * centery],

[0, 0, 1.0]])

matrix = matrix.dot(matrix_trans)[0:2, :]

data['img'] = cv2.warpAffine(

np.uint8(data['img']),

matrix,

tuple(self.size),

flags=cv2.INTER_LINEAR,

borderMode=cv2.BORDER_CONSTANT,

borderValue=self.im_padding_value)

for key in data.get('gt_fields', []):

data[key] = cv2.warpAffine(

np.uint8(data[key]),

matrix,

tuple(self.size),

flags=cv2.INTER_NEAREST,

borderMode=cv2.BORDER_CONSTANT,

borderValue=self.label_padding_value)

return data

import cv2

import numpy as np

from PIL import Image, ImageEnhance

from scipy.ndimage import distance_transform_edt

def normalize(im, mean, std):

im = im.astype(np.float32, copy=False) / 255.0

im -= mean

im /= std

return im

def resize(im, target_size=608, interp=cv2.INTER_LINEAR):

if isinstance(target_size, list) or isinstance(target_size, tuple):

w = target_size[0]

h = target_size[1]

else:

w = target_size

h = target_size

im = cv2.resize(im, (w, h), interpolation=interp)

return im

def resize_long(im, long_size=224, interpolation=cv2.INTER_LINEAR):

value = max(im.shape[0], im.shape[1])

scale = float(long_size) / float(value)

resized_width = int(round(im.shape[1] * scale))

resized_height = int(round(im.shape[0] * scale))

im = cv2.resize(

im, (resized_width, resized_height), interpolation=interpolation)

return im

def resize_short(im, short_size=224, interpolation=cv2.INTER_LINEAR):

value = min(im.shape[0], im.shape[1])

scale = float(short_size) / float(value)

resized_width = int(round(im.shape[1] * scale))

resized_height = int(round(im.shape[0] * scale))

im = cv2.resize(

im, (resized_width, resized_height), interpolation=interpolation)

return im

def horizontal_flip(im):

if len(im.shape) == 3:

im = im[:, ::-1, :]

elif len(im.shape) == 2:

im = im[:, ::-1]

return im

def vertical_flip(im):

if len(im.shape) == 3:

im = im[::-1, :, :]

elif len(im.shape) == 2:

im = im[::-1, :]

return im

def brightness(im, brightness_lower, brightness_upper):

brightness_delta = np.random.uniform(brightness_lower, brightness_upper)

im = ImageEnhance.Brightness(im).enhance(brightness_delta)

return im

def contrast(im, contrast_lower, contrast_upper):

contrast_delta = np.random.uniform(contrast_lower, contrast_upper)

im = ImageEnhance.Contrast(im).enhance(contrast_delta)

return im

def saturation(im, saturation_lower, saturation_upper):

saturation_delta = np.random.uniform(saturation_lower, saturation_upper)

im = ImageEnhance.Color(im).enhance(saturation_delta)

return im

def hue(im, hue_lower, hue_upper):

hue_delta = np.random.uniform(hue_lower, hue_upper)

im = np.array(im.convert('HSV'))

im[:, :, 0] = im[:, :, 0] + hue_delta

im = Image.fromarray(im, mode='HSV').convert('RGB')

return im

def sharpness(im, sharpness_lower, sharpness_upper):

sharpness_delta = np.random.uniform(sharpness_lower, sharpness_upper)

im = ImageEnhance.Sharpness(im).enhance(sharpness_delta)

return im

def rotate(im, rotate_lower, rotate_upper):

rotate_delta = np.random.uniform(rotate_lower, rotate_upper)

im = im.rotate(int(rotate_delta))

return im

def mask_to_onehot(mask, num_classes):

"""

Convert a mask (H, W) to onehot (K, H, W).

Args:

mask (np.ndarray): Label mask with shape (H, W)

num_classes (int): Number of classes.

Returns:

np.ndarray: Onehot mask with shape(K, H, W).

"""

_mask = [mask == i for i in range(num_classes)]

_mask = np.array(_mask).astype(np.uint8)

return _mask

def onehot_to_binary_edge(mask, radius):

"""

Convert a onehot mask (K, H, W) to a edge mask.

Args:

mask (np.ndarray): Onehot mask with shape (K, H, W)

radius (int|float): Radius of edge.

Returns:

np.ndarray: Edge mask with shape(H, W).

"""

if radius < 1:

raise ValueError('`radius` should be greater than or equal to 1')

num_classes = mask.shape[0]

edge = np.zeros(mask.shape[1:])

\# pad borders

mask = np.pad(mask, ((0, 0), (1, 1), (1, 1)),

mode='constant',

constant_values=0)

for i in range(num_classes):

dist = distance_transform_edt(mask[i, :]) + distance_transform_edt(

1.0 - mask[i, :])

dist = dist[1:-1, 1:-1]

dist[dist > radius] = 0

edge += dist

edge = np.expand_dims(edge, axis=0)

edge = (edge > 0).astype(np.uint8)

return edge

def mask_to_binary_edge(mask, radius, num_classes):

"""

Convert a segmentic segmentation mask (H, W) to a binary edge mask(H, W).

Args:

mask (np.ndarray): Label mask with shape (H, W)

radius (int|float): Radius of edge.

num_classes (int): Number of classes.

Returns:

np.ndarray: Edge mask with shape(H, W).

"""

mask = mask.squeeze()

onehot = mask_to_onehot(mask, num_classes)

edge = onehot_to_binary_edge(onehot, radius)

return edge

@manager.MODELS.add_component

class GINet(nn.Layer):

"""

The GINet implementation based on PaddlePaddle.

The original article refers to

Wu, Tianyi, Yu Lu, Yu Zhu, Chuang Zhang, Ming Wu, Zhanyu Ma, and Guodong Guo. "GINet: Graph interaction network for scene parsing." In European Conference on Computer Vision, pp. 34-51. Springer, Cham, 2020.

(https://arxiv.org/pdf/2009.06160).

Args:

num_classes (int): The unique number of target classes.

backbone (Paddle.nn.Layer): Backbone network.

backbone_indices (tuple, optional): Values in the tuple indicate the indices of output of backbone.

enable_auxiliary_loss (bool, optional): A bool value indicates whether adding auxiliary loss.

If true, auxiliary loss will be added after LearningToDownsample module. Default: False.

align_corners (bool): An argument of F.interpolate. It should be set to False when the output size of feature

is even, e.g. 1024x512, otherwise it is True, e.g. 769x769.. Default: False.

jpu (bool, optional)): whether to use jpu unit in the base forward. Default:True.

pretrained (str, optional): The path or url of pretrained model. Default: None.

"""

def __init__(self,

num_classes,

backbone,

backbone_indices=[0, 1, 2, 3],

enable_auxiliary_loss=True,

align_corners=True,

jpu=True,

pretrained=None):

super().__init__()

self.nclass = num_classes

self.aux = enable_auxiliary_loss

self.jpu = jpu

self.backbone = backbone

self.backbone_indices = backbone_indices

self.align_corners = align_corners

self.jpu = layers.JPU([512, 1024, 2048], width=512) if jpu else None

self.head = GIHead(in_channels=2048, nclass=num_classes)

if self.aux:

self.auxlayer = layers.AuxLayer(

1024, 1024 // 4, num_classes, bias_attr=False)

self.pretrained = pretrained

self.init_weight()

def base_forward(self, x):

feat_list = self.backbone(x)

c1, c2, c3, c4 = [feat_list[i] for i in self.backbone_indices]

if self.jpu:

return self.jpu(c1, c2, c3, c4)

else:

return c1, c2, c3, c4

def forward(self, x):

_, _, h, w = paddle.shape(x)

_, _, c3, c4 = self.base_forward(x)

logit_list = []

x, _ = self.head(c4)

logit_list.append(x)

if self.aux:

auxout = self.auxlayer(c3)

logit_list.append(auxout)

return [

F.interpolate(

logit, [h, w],

mode='bilinear',

align_corners=self.align_corners) for logit in logit_list

]

def init_weight(self):

if self.pretrained is not None:

utils.load_entire_model(self, self.pretrained)

class GIHead(nn.Layer):

"""The Graph Interaction Network head."""

def __init__(self, in_channels, nclass):

super().__init__()

self.nclass = nclass

inter_channels = in_channels // 4

self.inp = paddle.zeros(shape=(nclass, 300), dtype='float32')

self.inp = paddle.create_parameter(

shape=self.inp.shape,

dtype=str(self.inp.numpy().dtype),

default_initializer=paddle.nn.initializer.Assign(self.inp))

self.inp.stop_gradient = True

self.fc1 = nn.Sequential(

nn.Linear(300, 128), nn.BatchNorm1D(128), nn.ReLU())

self.fc2 = nn.Sequential(

nn.Linear(128, 256), nn.BatchNorm1D(256), nn.ReLU())

self.conv5 = layers.ConvBNReLU(

in_channels,

inter_channels,

3,

padding=1,

bias_attr=False,

stride=1)

self.gloru = GlobalReasonUnit(

in_channels=inter_channels,

num_state=256,

num_node=84,

nclass=nclass)

self.conv6 = nn.Sequential(

nn.Dropout(0.1), nn.Conv2D(inter_channels, nclass, 1))

def forward(self, x):

B, C, H, W = paddle.shape(x)

inp = self.inp

inp = self.fc1(inp)

inp = self.fc2(inp).unsqueeze(axis=0).transpose((0, 2, 1))\

.expand((B, 256, self.nclass))

out = self.conv5(x)

out, se_out = self.gloru(out, inp)

out = self.conv6(out)

return out, se_out

class GlobalReasonUnit(nn.Layer):

"""

The original paper refers to:

Chen, Yunpeng, et al. "Graph-Based Global Reasoning Networks" (https://arxiv.org/abs/1811.12814)

"""

def __init__(self, in_channels, num_state=256, num_node=84, nclass=59):

super().__init__()

self.num_state = num_state

self.conv_theta = nn.Conv2D(

in_channels, num_node, kernel_size=1, stride=1, padding=0)

self.conv_phi = nn.Conv2D(

in_channels, num_state, kernel_size=1, stride=1, padding=0)

self.graph = GraphLayer(num_state, num_node, nclass)

self.extend_dim = nn.Conv2D(

num_state, in_channels, kernel_size=1, bias_attr=False)

self.bn = layers.SyncBatchNorm(in_channels)

def forward(self, x, inp):

B = self.conv_theta(x)

sizeB = paddle.shape(B)

B = paddle.flatten(B, 2, 3)

sizex = paddle.shape(x)

x_reduce = self.conv_phi(x)

x_reduce = paddle.flatten(x_reduce, 2, 3).transpose((0, 2, 1))

V = paddle.bmm(B, x_reduce).transpose((0, 2, 1))

V = paddle.divide(V, (sizex[2] * sizex[3]).astype('float32'))

class_node, new_V = self.graph(inp, V)

D = B.transpose((0, 2, 1))

Y = paddle.bmm(D, new_V.transpose((0, 2, 1)))

Y = Y.transpose((0, 2, 1)).reshape((sizex[0], self.num_state, \

sizex[2], -1))

Y = self.extend_dim(Y)

Y = self.bn(Y)

out = Y + x

return out, class_node

class GraphLayer(nn.Layer):

def __init__(self, num_state, num_node, num_class):

super().__init__()

self.vis_gcn = GCN(num_state, num_node)

self.word_gcn = GCN(num_state, num_class)

self.transfer = GraphTransfer(num_state)

self.gamma_vis = paddle.zeros([num_node])

self.gamma_word = paddle.zeros([num_class])

self.gamma_vis = paddle.create_parameter(

shape=paddle.shape(self.gamma_vis),

dtype=str(self.gamma_vis.numpy().dtype),

default_initializer=paddle.nn.initializer.Assign(self.gamma_vis))

self.gamma_word = paddle.create_parameter(

shape=paddle.shape(self.gamma_word),

dtype=str(self.gamma_word.numpy().dtype),

default_initializer=paddle.nn.initializer.Assign(self.gamma_word))

def forward(self, inp, vis_node):

inp = self.word_gcn(inp)

new_V = self.vis_gcn(vis_node)

class_node, vis_node = self.transfer(inp, new_V)

class_node = self.gamma_word * inp + class_node

new_V = self.gamma_vis * vis_node + new_V

return class_node, new_V

class GCN(nn.Layer):

def __init__(self, num_state=128, num_node=64, bias=False):

super().__init__()

self.conv1 = nn.Conv1D(

num_node,

num_node,

kernel_size=1,

padding=0,

stride=1,

groups=1, )

self.relu = nn.ReLU()

self.conv2 = nn.Conv1D(

num_state,

num_state,

kernel_size=1,

padding=0,

stride=1,

groups=1,

bias_attr=bias)

def forward(self, x):

h = self.conv1(x.transpose((0, 2, 1))).transpose((0, 2, 1))

h = h + x

h = self.relu(h)

h = self.conv2(h)

return h

class GraphTransfer(nn.Layer):

"""Transfer vis graph to class node, transfer class node to vis feature"""

def __init__(self, in_dim):

super().__init__()

self.channle_in = in_dim

self.query_conv = nn.Conv1D(

in_channels=in_dim, out_channels=in_dim // 2, kernel_size=1)

self.key_conv = nn.Conv1D(

in_channels=in_dim, out_channels=in_dim // 2, kernel_size=1)

self.value_conv_vis = nn.Conv1D(

in_channels=in_dim, out_channels=in_dim, kernel_size=1)

self.value_conv_word = nn.Conv1D(

in_channels=in_dim, out_channels=in_dim, kernel_size=1)

self.softmax_vis = nn.Softmax(axis=-1)

self.softmax_word = nn.Softmax(axis=-2)

def forward(self, word, vis_node):

m_batchsize, C, Nc = paddle.shape(word)

m_batchsize, C, Nn = paddle.shape(vis_node)

proj_query = self.query_conv(word).reshape((m_batchsize, -1, Nc))\

.transpose((0, 2, 1))

proj_key = self.key_conv(vis_node).reshape((m_batchsize, -1, Nn))

energy = paddle.bmm(proj_query, proj_key)

attention_vis = self.softmax_vis(energy).transpose((0, 2, 1))

attention_word = self.softmax_word(energy)

proj_value_vis = self.value_conv_vis(vis_node).reshape((m_batchsize, -1,

Nn))

proj_value_word = self.value_conv_word(word).reshape((m_batchsize, -1,

Nc))

class_out = paddle.bmm(proj_value_vis, attention_vis)

node_out = paddle.bmm(proj_value_word, attention_word)

return class_out, node_out

import paddle

import paddle.nn as nn

import paddle.nn.functional as F

from paddleseg.cvlibs import manager

from paddleseg.models import layers

from paddleseg.utils import utils

@manager.MODELS.add_component

class DMNet(nn.Layer):

"""

The DMNet implementation based on PaddlePaddle.

The original article refers to

Junjun He, Zhongying Deng, Yu Qiao. "Dynamic Multi-scale Filters for Semantic Segmentation"

Args:

num_classes (int): The unique number of target classes.

backbone (paddle.nn.Layer): Backbone network, currently support Resnet50_vd/Resnet101_vd.

mid_channels (int): The middle channels of convolution layer. Default: 512.

filter_sizes (list, tuple): The filter size of generated convolution kernel used in Dynamic Convolutional Module. Default: [1, 3, 5, 7].

fusion (bool): Add one conv to fuse DCM output feature. Default: False.

pretrained (str, optional): The path or url of pretrained model. Default: None.

"""

def __init__(self,

num_classes,

backbone,

mid_channels=512,

filter_sizes=[1, 3, 5, 7],

fusion=False,

pretrained=None):

super().__init__()

self.backbone = backbone

self.dcm_modules = nn.LayerList()

for filter_size in filter_sizes:

self.dcm_modules.append(

DCM(filter_size, fusion, self.backbone.feat_channels[-1],

mid_channels), )

self.bottleneck = layers.ConvBNReLU(

self.backbone.feat_channels[-1] + len(filter_sizes) * mid_channels,

mid_channels,

3,

padding=1, )

self.cls = nn.Conv2D(mid_channels, num_classes, 1)

self.fcn_head = nn.Sequential(

layers.ConvBNReLU(

self.backbone.feat_channels[2], mid_channels, 3, padding=1),

nn.Conv2D(mid_channels, num_classes, 1), )

self.pretrained = pretrained

self.init_weight()

def init_weight(self):

if self.pretrained is not None:

utils.load_entire_model(self, self.pretrained)

def forward(self, x):

feats = self.backbone(x)

x = feats[-1]

dcm_outs = [x]

for dcm_module in self.dcm_modules:

dcm_outs.append(dcm_module(x))

dcm_outs = paddle.concat(dcm_outs, axis=1)

x = self.bottleneck(dcm_outs)

x = self.cls(x)

x = F.interpolate(

x, scale_factor=8, mode='bilinear', align_corners=True)

output = [x]

if self.training:

fcn_out = self.fcn_head(feats[2])

fcn_out = F.interpolate(

fcn_out, scale_factor=8, mode='bilinear', align_corners=True)

output.append(fcn_out)

return output

return output

class DCM(nn.Layer):

"""

Dynamic Convolutional Module used in DMNet.

Args:

filter_size (int): The filter size of generated convolution kernel used in Dynamic Convolutional Module.

fusion (bool): Add one conv to fuse DCM output feature.

in_channels (int): Input channels.

channels (int): Channels after modules, before conv_seg.

"""

def __init__(self, filter_size, fusion, in_channels, channels):

super().__init__()

self.filter_size = filter_size

self.fusion = fusion

self.channels = channels

pad = (self.filter_size - 1) // 2

if (self.filter_size - 1) % 2 == 0:

self.pad = (pad, pad, pad, pad)

else:

self.pad = (pad + 1, pad, pad + 1, pad)

self.avg_pool = nn.AdaptiveAvgPool2D(filter_size)

self.filter_gen_conv = nn.Conv2D(in_channels, channels, 1)

self.input_redu_conv = layers.ConvBNReLU(in_channels, channels, 1)

self.norm = layers.SyncBatchNorm(channels)

self.act = nn.ReLU()

if self.fusion:

self.fusion_conv = layers.ConvBNReLU(channels, channels, 1)

def forward(self, x):

generated_filter = self.filter_gen_conv(self.avg_pool(x))

x = self.input_redu_conv(x)

b, c, h, w = x.shape

x = x.reshape([1, b * c, h, w])

generated_filter = generated_filter.reshape(

[b * c, 1, self.filter_size, self.filter_size])

x = F.pad(x, self.pad, mode='constant', value=0)

output = F.conv2d(x, weight=generated_filter, groups=b * c)

output = output.reshape([b, self.channels, h, w])

output = self.norm(output)

output = self.act(output)

if self.fusion:

output = self.fusion_conv(output)

return output

@manager.MODELS.add_component

class DeepLabV3P(nn.Layer):

"""

The DeepLabV3Plus implementation based on PaddlePaddle.

The original article refers to

Liang-Chieh Chen, et, al. "Encoder-Decoder with Atrous Separable Convolution for Semantic Image Segmentation"

(https://arxiv.org/abs/1802.02611)

Args:

num_classes (int): The unique number of target classes.

backbone (paddle.nn.Layer): Backbone network, currently support Resnet50_vd/Resnet101_vd/Xception65.

backbone_indices (tuple, optional): Two values in the tuple indicate the indices of output of backbone.

Default: (0, 3).

aspp_ratios (tuple, optional): The dilation rate using in ASSP module.

If output_stride=16, aspp_ratios should be set as (1, 6, 12, 18).

If output_stride=8, aspp_ratios is (1, 12, 24, 36).

Default: (1, 6, 12, 18).

aspp_out_channels (int, optional): The output channels of ASPP module. Default: 256.

align_corners (bool, optional): An argument of F.interpolate. It should be set to False when the feature size is even,

e.g. 1024x512, otherwise it is True, e.g. 769x769. Default: False.

pretrained (str, optional): The path or url of pretrained model. Default: None.

data_format(str, optional): Data format that specifies the layout of input. It can be "NCHW" or "NHWC". Default: "NCHW".

"""

def __init__(self,

num_classes,

backbone,

backbone_indices=(0, 3),

aspp_ratios=(1, 6, 12, 18),

aspp_out_channels=256,

align_corners=False,

pretrained=None,

data_format="NCHW"):

super().__init__()

self.backbone = backbone

backbone_channels = [

backbone.feat_channels[i] for i in backbone_indices

]

self.head = DeepLabV3PHead(

num_classes,

backbone_indices,

backbone_channels,

aspp_ratios,

aspp_out_channels,

align_corners,

data_format=data_format)

self.align_corners = align_corners

self.pretrained = pretrained

self.data_format = data_format

self.init_weight()

def forward(self, x):

feat_list = self.backbone(x)

logit_list = self.head(feat_list)

if self.data_format == 'NCHW':

ori_shape = paddle.shape(x)[2:]

else:

ori_shape = paddle.shape(x)[1:3]

return [

F.interpolate(

logit,

ori_shape,

mode='bilinear',

align_corners=self.align_corners,

data_format=self.data_format) for logit in logit_list

]

def init_weight(self):

if self.pretrained is not None:

utils.load_entire_model(self, self.pretrained)

class DeepLabV3PHead(nn.Layer):

"""

The DeepLabV3PHead implementation based on PaddlePaddle.

Args:

num_classes (int): The unique number of target classes.

backbone_indices (tuple): Two values in the tuple indicate the indices of output of backbone.

the first index will be taken as a low-level feature in Decoder component;

the second one will be taken as input of ASPP component.

Usually backbone consists of four downsampling stage, and return an output of

each stage. If we set it as (0, 3), it means taking feature map of the first

stage in backbone as low-level feature used in Decoder, and feature map of the fourth

stage as input of ASPP.

backbone_channels (tuple): The same length with "backbone_indices". It indicates the channels of corresponding index.

aspp_ratios (tuple): The dilation rates using in ASSP module.

aspp_out_channels (int): The output channels of ASPP module.

align_corners (bool): An argument of F.interpolate. It should be set to False when the output size of feature

is even, e.g. 1024x512, otherwise it is True, e.g. 769x769.

data_format(str, optional): Data format that specifies the layout of input. It can be "NCHW" or "NHWC". Default: "NCHW".

"""

def __init__(self,

num_classes,

backbone_indices,

backbone_channels,

aspp_ratios,

aspp_out_channels,

align_corners,

data_format='NCHW'):

super().__init__()

self.aspp = layers.ASPPModule(

aspp_ratios,

backbone_channels[1],

aspp_out_channels,

align_corners,

use_sep_conv=True,

image_pooling=True,

data_format=data_format)

self.decoder = Decoder(

num_classes,

backbone_channels[0],

align_corners,

data_format=data_format)

self.backbone_indices = backbone_indices

def forward(self, feat_list):

logit_list = []

low_level_feat = feat_list[self.backbone_indices[0]]

x = feat_list[self.backbone_indices[1]]

x = self.aspp(x)

logit = self.decoder(x, low_level_feat)

logit_list.append(logit)

return logit_list

@manager.MODELS.add_component

class DeepLabV3(nn.Layer):

"""

The DeepLabV3 implementation based on PaddlePaddle.

The original article refers to

Liang-Chieh Chen, et, al. "Rethinking Atrous Convolution for Semantic Image Segmentation"

(https://arxiv.org/pdf/1706.05587.pdf).

Args:

Please Refer to DeepLabV3P above.

"""

def __init__(self,

num_classes,

backbone,

backbone_indices=(3, ),

aspp_ratios=(1, 6, 12, 18),

aspp_out_channels=256,

align_corners=False,

pretrained=None):

super().__init__()

self.backbone = backbone

backbone_channels = [

backbone.feat_channels[i] for i in backbone_indices

]

self.head = DeepLabV3Head(num_classes, backbone_indices,

backbone_channels, aspp_ratios,

aspp_out_channels, align_corners)

self.align_corners = align_corners

self.pretrained = pretrained

self.init_weight()

def forward(self, x):

feat_list = self.backbone(x)

logit_list = self.head(feat_list)

return [

F.interpolate(

logit,

paddle.shape(x)[2:],

mode='bilinear',

align_corners=self.align_corners) for logit in logit_list

]

def init_weight(self):

if self.pretrained is not None:

utils.load_entire_model(self, self.pretrained)

class DeepLabV3Head(nn.Layer):

"""

The DeepLabV3Head implementation based on PaddlePaddle.

Args:

Please Refer to DeepLabV3PHead above.

"""

def __init__(self, num_classes, backbone_indices, backbone_channels,

aspp_ratios, aspp_out_channels, align_corners):

super().__init__()

self.aspp = layers.ASPPModule(

aspp_ratios,

backbone_channels[0],

aspp_out_channels,

align_corners,

use_sep_conv=False,

image_pooling=True)

self.cls = nn.Conv2D(

in_channels=aspp_out_channels,

out_channels=num_classes,

kernel_size=1)

self.backbone_indices = backbone_indices

def forward(self, feat_list):

logit_list = []

x = feat_list[self.backbone_indices[0]]

x = self.aspp(x)

logit = self.cls(x)

logit_list.append(logit)

return logit_list

class Decoder(nn.Layer):

"""

Decoder module of DeepLabV3P model

Args:

num_classes (int): The number of classes.

in_channels (int): The number of input channels in decoder module.

"""

def __init__(self,

num_classes,

in_channels,

align_corners,

data_format='NCHW'):

super(Decoder, self).__init__()

self.data_format = data_format

self.conv_bn_relu1 = layers.ConvBNReLU(

in_channels=in_channels,

out_channels=48,

kernel_size=1,

data_format=data_format)

self.conv_bn_relu2 = layers.SeparableConvBNReLU(

in_channels=304,

out_channels=256,

kernel_size=3,

padding=1,

data_format=data_format)

self.conv_bn_relu3 = layers.SeparableConvBNReLU(

in_channels=256,

out_channels=256,

kernel_size=3,

padding=1,

data_format=data_format)

self.conv = nn.Conv2D(

in_channels=256,

out_channels=num_classes,

kernel_size=1,

data_format=data_format)

self.align_corners = align_corners

def forward(self, x, low_level_feat):

low_level_feat = self.conv_bn_relu1(low_level_feat)

if self.data_format == 'NCHW':

low_level_shape = paddle.shape(low_level_feat)[-2:]

axis = 1

else:

low_level_shape = paddle.shape(low_level_feat)[1:3]

axis = -1

x = F.interpolate(

x,

low_level_shape,

mode='bilinear',

align_corners=self.align_corners,

data_format=self.data_format)

x = paddle.concat([x, low_level_feat], axis=axis)

x = self.conv_bn_relu2(x)

x = self.conv_bn_relu3(x)

x = self.conv(x)

return x

import cv2

import numpy as np

import paddle

import paddle.nn as nn

import paddle.nn.functional as F

from paddleseg.cvlibs import manager

from paddleseg.models import layers

from paddleseg.models.backbones import resnet_vd

from paddleseg.models import deeplab

from paddleseg.utils import utils

@manager.MODELS.add_component

class DecoupledSegNet(nn.Layer):

"""

The DecoupledSegNet implementation based on PaddlePaddle.

The original article refers to

Xiangtai Li, et, al. "Improving Semantic Segmentation via Decoupled Body and Edge Supervision"

(https://arxiv.org/pdf/2007.10035.pdf)

Args:

num_classes (int): The unique number of target classes.

backbone (paddle.nn.Layer): Backbone network, currently support Resnet50_vd/Resnet101_vd.

backbone_indices (tuple, optional): Two values in the tuple indicate the indices of output of backbone.

Default: (0, 3).

aspp_ratios (tuple, optional): The dilation rate using in ASSP module.

If output_stride=16, aspp_ratios should be set as (1, 6, 12, 18).

If output_stride=8, aspp_ratios is (1, 12, 24, 36).

Default: (1, 6, 12, 18).

aspp_out_channels (int, optional): The output channels of ASPP module. Default: 256.

align_corners (bool, optional): An argument of F.interpolate. It should be set to False when the feature size is even,

e.g. 1024x512, otherwise it is True, e.g. 769x769. Default: False.

pretrained (str, optional): The path or url of pretrained model. Default: None.

"""

def __init__(self,

num_classes,

backbone,

backbone_indices=(0, 3),

aspp_ratios=(1, 6, 12, 18),

aspp_out_channels=256,

align_corners=False,

pretrained=None):

super().__init__()

self.backbone = backbone

backbone_channels = self.backbone.feat_channels

self.head = DecoupledSegNetHead(num_classes, backbone_indices,

backbone_channels, aspp_ratios,

aspp_out_channels, align_corners)

self.align_corners = align_corners

self.pretrained = pretrained

self.init_weight()

def forward(self, x):

feat_list = self.backbone(x)

logit_list = self.head(feat_list)

seg_logit, body_logit, edge_logit = [

F.interpolate(

logit,

paddle.shape(x)[2:],

mode='bilinear',

align_corners=self.align_corners) for logit in logit_list

]

if self.training:

return [seg_logit, body_logit, edge_logit, (seg_logit, edge_logit)]

return [seg_logit]

def init_weight(self):

if self.pretrained is not None:

utils.load_entire_model(self, self.pretrained)

class DecoupledSegNetHead(nn.Layer):

"""

The DecoupledSegNetHead implementation based on PaddlePaddle.

Args:

num_classes (int): The unique number of target classes.

backbone_indices (tuple): Two values in the tuple indicate the indices of output of backbone.

the first index will be taken as a low-level feature in Edge presevation component;

the second one will be taken as input of ASPP component.

backbone_channels (tuple): The channels of output of backbone.

aspp_ratios (tuple): The dilation rates using in ASSP module.

aspp_out_channels (int): The output channels of ASPP module.

align_corners (bool): An argument of F.interpolate. It should be set to False when the output size of feature

is even, e.g. 1024x512, otherwise it is True, e.g. 769x769.

"""

def __init__(self, num_classes, backbone_indices, backbone_channels,

aspp_ratios, aspp_out_channels, align_corners):

super().__init__()

self.backbone_indices = backbone_indices

self.align_corners = align_corners

self.aspp = layers.ASPPModule(

aspp_ratios=aspp_ratios,

in_channels=backbone_channels[backbone_indices[1]],

out_channels=aspp_out_channels,

align_corners=align_corners,

image_pooling=True)

self.bot_fine = nn.Conv2D(

backbone_channels[backbone_indices[0]], 48, 1, bias_attr=False)

\# decoupled

self.squeeze_body_edge = SqueezeBodyEdge(

256, align_corners=self.align_corners)

self.edge_fusion = nn.Conv2D(256 + 48, 256, 1, bias_attr=False)

self.sigmoid_edge = nn.Sigmoid()

self.edge_out = nn.Sequential(

layers.ConvBNReLU(

in_channels=256,

out_channels=48,

kernel_size=3,

bias_attr=False),

nn.Conv2D(

48, 1, 1, bias_attr=False))

self.dsn_seg_body = nn.Sequential(

layers.ConvBNReLU(

in_channels=256,

out_channels=256,

kernel_size=3,

bias_attr=False),

nn.Conv2D(

256, num_classes, 1, bias_attr=False))

self.final_seg = nn.Sequential(

layers.ConvBNReLU(

in_channels=512,

out_channels=256,

kernel_size=3,

bias_attr=False),

layers.ConvBNReLU(

in_channels=256,

out_channels=256,

kernel_size=3,

bias_attr=False),

nn.Conv2D(

256, num_classes, kernel_size=1, bias_attr=False))

def forward(self, feat_list):

fine_fea = feat_list[self.backbone_indices[0]]

fine_size = paddle.shape(fine_fea)

x = feat_list[self.backbone_indices[1]]

aspp = self.aspp(x)

\# decoupled

seg_body, seg_edge = self.squeeze_body_edge(aspp)

\# Edge presevation and edge out

fine_fea = self.bot_fine(fine_fea)

seg_edge = F.interpolate(

seg_edge,

fine_size[2:],

mode='bilinear',

align_corners=self.align_corners)

seg_edge = self.edge_fusion(paddle.concat([seg_edge, fine_fea], axis=1))

seg_edge_out = self.edge_out(seg_edge)

seg_edge_out = self.sigmoid_edge(seg_edge_out) # seg_edge output

seg_body_out = self.dsn_seg_body(seg_body) # body out

\# seg_final out

seg_out = seg_edge + F.interpolate(

seg_body,

fine_size[2:],

mode='bilinear',

align_corners=self.align_corners)

aspp = F.interpolate(

aspp,

fine_size[2:],

mode='bilinear',

align_corners=self.align_corners)

seg_out = paddle.concat([aspp, seg_out], axis=1)

seg_final_out = self.final_seg(seg_out)

return [seg_final_out, seg_body_out, seg_edge_out]

class SqueezeBodyEdge(nn.Layer):

def __init__(self, inplane, align_corners=False):

super().__init__()

self.align_corners = align_corners

self.down = nn.Sequential(

layers.ConvBNReLU(

inplane, inplane, kernel_size=3, groups=inplane, stride=2),

layers.ConvBNReLU(

inplane, inplane, kernel_size=3, groups=inplane, stride=2))

self.flow_make = nn.Conv2D(

inplane * 2, 2, kernel_size=3, padding='same', bias_attr=False)

def forward(self, x):

size = paddle.shape(x)[2:]

seg_down = self.down(x)

seg_down = F.interpolate(

seg_down,

size=size,

mode='bilinear',

align_corners=self.align_corners)

flow = self.flow_make(paddle.concat([x, seg_down], axis=1))

seg_flow_warp = self.flow_warp(x, flow, size)

seg_edge = x - seg_flow_warp

return seg_flow_warp, seg_edge

def flow_warp(self, input, flow, size):

input_shape = paddle.shape(input)

norm = size[::-1].reshape([1, 1, 1, -1])

norm.stop_gradient = True

h_grid = paddle.linspace(-1.0, 1.0, size[0]).reshape([-1, 1])

h_grid = h_grid.tile([size[1]])

w_grid = paddle.linspace(-1.0, 1.0, size[1]).reshape([-1, 1])

w_grid = w_grid.tile([size[0]]).transpose([1, 0])

grid = paddle.concat([w_grid.unsqueeze(2), h_grid.unsqueeze(2)], axis=2)

grid.unsqueeze(0).tile([input_shape[0], 1, 1, 1])

grid = grid + paddle.transpose(flow, (0, 2, 3, 1)) / norm

output = F.grid_sample(input, grid)

return output

import paddle

import paddle.nn as nn

import paddle.nn.functional as F

from paddleseg.cvlibs import manager, param_init

from paddleseg.models import layers

from paddleseg.utils import utils

class DualResNet(nn.Layer):

"""

The DDRNet implementation based on PaddlePaddle.

The original article refers to

Yuanduo Hong, Huihui Pan, Weichao Sun, et al. "Deep Dual-resolution Networks for Real-time and Accurate Semantic Segmentation of Road Scenes"

(https://arxiv.org/abs/2101.06085)

Args:

num_classes (int): The unique number of target classes.

in_channels (int, optional): Number of input channels. Default: 3.

block_layers (list, tuple): The numbers of layers in different blocks. Default: [2, 2, 2, 2].

planes (int): Base channels in network. Default: 64.

spp_planes (int): Branch channels for DAPPM. Default: 128.

head_planes (int): Mid channels of segmentation head. Default: 128.

enable_auxiliary_loss (bool): Whether use auxiliary head for stage3. Default: False.

pretrained (str, optional): The path or url of pretrained model. Default: None.

"""

def __init__(self,

num_classes,

in_channels=3,

block_layers=[2, 2, 2, 2],

planes=64,

spp_planes=128,

head_planes=128,

enable_auxiliary_loss=False,

pretrained=None):

super().__init__()

highres_planes = planes * 2

self.enable_auxiliary_loss = enable_auxiliary_loss

self.conv1 = nn.Sequential(

layers.ConvBNReLU(

in_channels, planes, kernel_size=3, stride=2, padding=1),

layers.ConvBNReLU(

planes, planes, kernel_size=3, stride=2, padding=1), )

self.relu = nn.ReLU()

self.layer1 = self._make_layers(BasicBlock, planes, planes,

block_layers[0])

self.layer2 = self._make_layers(

BasicBlock, planes, planes * 2, block_layers[1], stride=2)

self.layer3 = self._make_layers(

BasicBlock, planes * 2, planes * 4, block_layers[2], stride=2)

self.layer4 = self._make_layers(

BasicBlock, planes * 4, planes * 8, block_layers[3], stride=2)

self.compression3 = layers.ConvBN(

planes * 4, highres_planes, kernel_size=1, bias_attr=False)

self.compression4 = layers.ConvBN(

planes * 8, highres_planes, kernel_size=1, bias_attr=False)

self.down3 = layers.ConvBN(

highres_planes,

planes * 4,

kernel_size=3,

stride=2,

bias_attr=False)

self.down4 = nn.Sequential(

layers.ConvBNReLU(

highres_planes,

planes * 4,

kernel_size=3,

stride=2,

padding=1,

bias_attr=False),

layers.ConvBN(

planes * 4,

planes * 8,

kernel_size=3,

stride=2,

padding=1,

bias_attr=False))

self.layer3_ = self._make_layers(BasicBlock, planes * 2, highres_planes,

2)

self.layer4_ = self._make_layers(BasicBlock, highres_planes,

highres_planes, 2)

self.layer5_ = self._make_layers(Bottleneck, highres_planes,

highres_planes, 1)

self.layer5 = self._make_layers(

Bottleneck, planes * 8, planes * 8, 1, stride=2)

self.spp = DAPPM(planes * 16, spp_planes, planes * 4)

if self.enable_auxiliary_loss:

self.aux_head = DDRNetHead(highres_planes, head_planes, num_classes)

self.head = DDRNetHead(planes * 4, head_planes, num_classes)

self.pretrained = pretrained

self.init_weight()

def init_weight(self):

if self.pretrained is not None:

utils.load_entire_model(self, self.pretrained)

else:

for m in self.sublayers():

if isinstance(m, nn.Conv2D):

param_init.kaiming_normal_init(m.weight)

elif isinstance(m, nn.BatchNorm2D):

param_init.constant_init(m.weight, value=1)

param_init.constant_init(m.bias, value=0)

def _make_layers(self, block, inplanes, planes, blocks, stride=1):

downsample = None

if stride != 1 or inplanes != planes * block.expansion:

downsample = nn.Sequential(

nn.Conv2D(

inplanes,

planes * block.expansion,

kernel_size=1,

stride=stride,

bias_attr=False),

nn.BatchNorm2D(planes * block.expansion), )

layers = []

layers.append(block(inplanes, planes, stride, downsample))

inplanes = planes * block.expansion

for i in range(1, blocks):

if i == (blocks - 1):

layers.append(block(inplanes, planes, stride=1, no_relu=True))

else:

layers.append(block(inplanes, planes, stride=1, no_relu=False))

return nn.Sequential(*layers)

def forward(self, x):

n, c, h, w = paddle.shape(x)

width_output = w // 8

height_output = h // 8

x = self.conv1(x)

stage1_out = self.layer1(x)

stage2_out = self.layer2(self.relu(stage1_out))

stage3_out = self.layer3(self.relu(stage2_out))

stage3_out_dual = self.layer3_(self.relu(stage2_out))

x = stage3_out + self.down3(self.relu(stage3_out_dual))

stage3_merge = stage3_out_dual + F.interpolate(

self.compression3(self.relu(stage3_out)),

size=[height_output, width_output],

mode='bilinear')

stage4_out = self.layer4(self.relu(x))

stage4_out_dual = self.layer4_(self.relu(stage3_merge))

x = stage4_out + self.down4(self.relu(stage4_out_dual))

stage4_merge = stage4_out_dual + F.interpolate(

self.compression4(self.relu(stage4_out)),

size=[height_output, width_output],

mode='bilinear')

stage5_out_dual = self.layer5_(self.relu(stage4_merge))

x = F.interpolate(

self.spp(self.layer5(self.relu(x))),

size=[height_output, width_output],

mode='bilinear')

output = self.head(x + stage5_out_dual)

logit_list = []

logit_list.append(output)

if self.enable_auxiliary_loss:

aux_out = self.aux_head(stage3_merge)

logit_list.append(aux_out)

return [

F.interpolate(

logit, [h, w], mode='bilinear') for logit in logit_list

]

class BasicBlock(nn.Layer):

expansion = 1

def __init__(self,

inplanes,

planes,

stride=1,

downsample=None,

no_relu=False):

super().__init__()

self.conv_bn_relu = layers.ConvBNReLU(

inplanes,

planes,

kernel_size=3,

stride=stride,

padding=1,

bias_attr=False)

self.relu = nn.ReLU()

self.conv_bn = layers.ConvBN(

planes, planes, kernel_size=3, stride=1, padding=1, bias_attr=False)

self.downsample = downsample

self.stride = stride

self.no_relu = no_relu

def forward(self, x):

residual = x

out = self.conv_bn_relu(x)

out = self.conv_bn(out)

if self.downsample is not None:

residual = self.downsample(x)

out += residual

if self.no_relu:

return out

else:

return self.relu(out)

class Bottleneck(nn.Layer):

expansion = 2

def __init__(self,

inplanes,

planes,

stride=1,

downsample=None,

no_relu=True):

super().__init__()

self.conv_bn_relu1 = layers.ConvBNReLU(

inplanes, planes, kernel_size=1, bias_attr=False)

self.conv_bn_relu2 = layers.ConvBNReLU(

planes,

planes,

kernel_size=3,

stride=stride,

padding=1,

bias_attr=False)

self.conv_bn = layers.ConvBN(

planes, planes * self.expansion, kernel_size=1, bias_attr=False)

self.relu = nn.ReLU()

self.downsample = downsample

self.stride = stride

self.no_relu = no_relu

def forward(self, x):

residual = x

out = self.conv_bn_relu1(x)

out = self.conv_bn_relu2(out)

out = self.conv_bn(out)

if self.downsample is not None:

residual = self.downsample(x)

out += residual

if self.no_relu:

return out

else:

return self.relu(out)

class DAPPM(nn.Layer):

def __init__(self, inplanes, branch_planes, outplanes):

super().__init__()

self.scale1 = nn.Sequential(

nn.AvgPool2D(

kernel_size=5, stride=2, padding=2),

layers.SyncBatchNorm(inplanes),

nn.ReLU(),

nn.Conv2D(

inplanes, branch_planes, kernel_size=1, bias_attr=False), )

self.scale2 = nn.Sequential(

nn.AvgPool2D(

kernel_size=9, stride=4, padding=4),

layers.SyncBatchNorm(inplanes),

nn.ReLU(),

nn.Conv2D(

inplanes, branch_planes, kernel_size=1, bias_attr=False), )

self.scale3 = nn.Sequential(

nn.AvgPool2D(

kernel_size=17, stride=8, padding=8),

layers.SyncBatchNorm(inplanes),

nn.ReLU(),

nn.Conv2D(

inplanes, branch_planes, kernel_size=1, bias_attr=False), )

self.scale4 = nn.Sequential(

nn.AdaptiveAvgPool2D((1, 1)),

layers.SyncBatchNorm(inplanes),

nn.ReLU(),

nn.Conv2D(

inplanes, branch_planes, kernel_size=1, bias_attr=False), )

self.scale0 = nn.Sequential(

layers.SyncBatchNorm(inplanes),

nn.ReLU(),

nn.Conv2D(

inplanes, branch_planes, kernel_size=1, bias_attr=False), )

self.process1 = nn.Sequential(

layers.SyncBatchNorm(branch_planes),

nn.ReLU(),

nn.Conv2D(

branch_planes,

branch_planes,

kernel_size=3,

padding=1,

bias_attr=False), )

self.process2 = nn.Sequential(

layers.SyncBatchNorm(branch_planes),

nn.ReLU(),

nn.Conv2D(

branch_planes,

branch_planes,

kernel_size=3,

padding=1,

bias_attr=False), )

self.process3 = nn.Sequential(

layers.SyncBatchNorm(branch_planes),

nn.ReLU(),

nn.Conv2D(

branch_planes,

branch_planes,

kernel_size=3,

padding=1,

bias_attr=False), )

self.process4 = nn.Sequential(

layers.SyncBatchNorm(branch_planes),

nn.ReLU(),

nn.Conv2D(

branch_planes,

branch_planes,

kernel_size=3,

padding=1,

bias_attr=False), )

self.compression = nn.Sequential(

layers.SyncBatchNorm(branch_planes * 5),

nn.ReLU(),

nn.Conv2D(

branch_planes * 5, outplanes, kernel_size=1, bias_attr=False))

self.shortcut = nn.Sequential(

layers.SyncBatchNorm(inplanes),

nn.ReLU(),

nn.Conv2D(

inplanes, outplanes, kernel_size=1, bias_attr=False))

def forward(self, x):

n, c, h, w = paddle.shape(x)

x0 = self.scale0(x)

x1 = self.process1(

F.interpolate(

self.scale1(x), size=[h, w], mode='bilinear') + x0)

x2 = self.process2(

F.interpolate(

self.scale2(x), size=[h, w], mode='bilinear') + x1)

x3 = self.process3(

F.interpolate(

self.scale3(x), size=[h, w], mode='bilinear') + x2)

x4 = self.process4(

F.interpolate(

self.scale4(x), size=[h, w], mode='bilinear') + x3)

out = self.compression(paddle.concat([x0, x1, x2, x3, x4],

1)) + self.shortcut(x)

return out

class DDRNetHead(nn.Layer):

def __init__(self, inplanes, interplanes, outplanes, scale_factor=None):

super().__init__()

self.bn1 = nn.BatchNorm2D(inplanes)

self.relu = nn.ReLU()

self.conv_bn_relu = layers.ConvBNReLU(

inplanes, interplanes, kernel_size=3, padding=1, bias_attr=False)

self.conv = nn.Conv2D(

interplanes, outplanes, kernel_size=1, padding=0, bias_attr=True)

self.scale_factor = scale_factor

def forward(self, x):

x = self.bn1(x)

x = self.relu(x)

x = self.conv_bn_relu(x)

out = self.conv(x)

if self.scale_factor is not None:

out = F.interpolate(

out, scale_factor=self.scale_factor, mode='bilinear')

return out

@manager.MODELS.add_component

def DDRNet_23(**kwargs):

return DualResNet(

block_layers=[2, 2, 2, 2],

planes=64,

spp_planes=128,

head_planes=128,

**kwargs)

@manager.MODELS.add_component

class BiseNetV1(nn.Layer):

"""

The BiSeNetV1 implementation based on PaddlePaddle.

The original article refers to

Yu, Changqian, et al. "BiSeNet V2: Bilateral Network with Guided Aggregation for Real-time Semantic Segmentation"

(https://paperswithcode.com/paper/bisenet-bilateral-segmentation-network-for)

Args:

num_classes (int): The unique number of target classes.

backbone (paddle.nn.Layer): Backbone network, currently support Resnet18_vd/Resnet34_vd/Resnet50_vd/Resnet101_vd.

pretrained (str, optional): The path or url of pretrained model. Default: None.

"""

def __init__(self, num_classes, backbone, conv_channel=128,

pretrained=None):

super().__init__()

self.backbone = backbone

self.spatial_path = SpatialPath(3, 128)

self.global_context = nn.Sequential(

nn.AdaptiveAvgPool2D(1),

layers.ConvBNReLU(

512, conv_channel, 1, bias_attr=False), )

self.arms = nn.LayerList([

AttentionRefinement(512, conv_channel),

AttentionRefinement(256, conv_channel),

])

self.refines = nn.LayerList([

layers.ConvBNReLU(

conv_channel,

conv_channel,

3,

stride=1,

padding=1,

bias_attr=False),

layers.ConvBNReLU(

conv_channel,

conv_channel,

3,

stride=1,

padding=1,

bias_attr=False),

])

self.heads = nn.LayerList([

BiSeNetHead(conv_channel, num_classes, 8, True),

BiSeNetHead(conv_channel, num_classes, 8, True),

BiSeNetHead(conv_channel * 2, num_classes, 8, False),

])

self.ffm = FeatureFusion(conv_channel * 2, conv_channel * 2, 1)

self.pretrained = pretrained

def init_weight(self):

if self.pretrained is not None:

utils.load_entire_model(self, self.pretrained)

def forward(self, x):

spatial_out = self.spatial_path(x)

context_blocks = self.backbone(x)

context_blocks.reverse()

global_context = self.global_context(context_blocks[0])

global_context = F.interpolate(

global_context,

size=paddle.shape(context_blocks[0])[2:],

mode='bilinear',

align_corners=True)

last_fm = global_context

pred_out = []

for i, (

fm, arm, refine

) in enumerate(zip(context_blocks[:2], self.arms, self.refines)):

fm = arm(fm)

fm += last_fm

last_fm = F.interpolate(

fm,

size=paddle.shape(context_blocks[i + 1])[2:],

mode='bilinear',

align_corners=True)

last_fm = refine(last_fm)

pred_out.append(last_fm)

context_out = last_fm

concate_fm = self.ffm(spatial_out, context_out)

pred_out.append(concate_fm)

output = []

if self.training:

for i, head in enumerate(self.heads):

out = head(pred_out[i])

output.append(out)

else:

out = self.heads[-1](pred_out[-1])

output.append(out)

return output

class SpatialPath(nn.Layer):

"""

SpatialPath module of BiseNetV1 model

Args:

in_channels (int): The number of input channels in spatial path module.

out_channels (int): The number of output channels in spatial path module.

"""

def __init__(self, in_channels, out_channels, inner_channel=64):

super().__init__()

self.conv_7x7 = layers.ConvBNReLU(

in_channels, inner_channel, 7, stride=2, padding=3, bias_attr=False)

self.conv_3x3_1 = layers.ConvBNReLU(

inner_channel,

inner_channel,

3,

stride=2,

padding=1,

bias_attr=False)

self.conv_3x3_2 = layers.ConvBNReLU(

inner_channel,

inner_channel,

3,

stride=2,

padding=1,

bias_attr=False)

self.conv_1x1 = layers.ConvBNReLU(

inner_channel, out_channels, 1, bias_attr=False)

def forward(self, x):

x = self.conv_7x7(x)

x = self.conv_3x3_1(x)

x = self.conv_3x3_2(x)

x = self.conv_1x1(x)

return x

class BiSeNetHead(nn.Layer):

"""

BiSeNet head of BiseNetV1 model

Args:

in_channels (int): The number of input channels in spatial path module.

out_channels (int): The number of output channels in spatial path module.

scale (int, float): The scale factor of interpolation.

"""

def __init__(self, in_channels, out_channels, scale, is_aux=False):

super().__init__()

inner_channel = 128 if is_aux else 64

self.conv_3x3 = layers.ConvBNReLU(

in_channels, inner_channel, 3, stride=1, padding=1, bias_attr=False)

self.conv_1x1 = nn.Conv2D(inner_channel, out_channels, 1)

self.scale = scale

def forward(self, x):

x = self.conv_3x3(x)

x = self.conv_1x1(x)

if self.scale > 1:

x = F.interpolate(

x, scale_factor=self.scale, mode='bilinear', align_corners=True)

return x

class AttentionRefinement(nn.Layer):

"""

AttentionRefinement module of BiseNetV1 model

Args:

in_channels (int): The number of input channels in spatial path module.

out_channels (int): The number of output channels in spatial path module.

"""

def __init__(self, in_channels, out_channels):

super().__init__()

self.conv_3x3 = layers.ConvBNReLU(

in_channels, out_channels, 3, stride=1, padding=1, bias_attr=False)

self.channel_attention = nn.Sequential(

nn.AdaptiveAvgPool2D(1),

layers.ConvBNReLU(

out_channels, out_channels, 1, bias_attr=False),

nn.Sigmoid(), )

def forward(self, x):

x = self.conv_3x3(x)

se = self.channel_attention(x)

x = x * se

return x

class FeatureFusion(nn.Layer):

"""

AttentionRefinement module of BiseNetV1 model

Args:

in_channels (int): The number of input channels in spatial path module.

out_channels (int): The number of output channels in spatial path module.

reduction (int): A factor shrinks convolutional channels. Default: 1.

"""

def __init__(self, in_channels, out_channels, reduction=1):

super().__init__()

self.conv_1x1 = layers.ConvBNReLU(

in_channels, out_channels, 1, bias_attr=False)

self.channel_attention = nn.Sequential(

nn.AdaptiveAvgPool2D(1),

layers.ConvBNReLU(

out_channels, out_channels // reduction, 1, bias_attr=False),

layers.ConvBNReLU(

out_channels // reduction, out_channels, 1, bias_attr=False),

nn.Sigmoid(), )

def forward(self, x1, x2):

fm = paddle.concat([x1, x2], axis=1)

fm = self.conv_1x1(fm)

fm_se = self.channel_attention(fm)

output = fm + fm * fm_se

return output

@manager.MODELS.add_component

class BiseNetV1(nn.Layer):

"""

The BiSeNetV1 implementation based on PaddlePaddle.

The original article refers to

Yu, Changqian, et al. "BiSeNet V2: Bilateral Network with Guided Aggregation for Real-time Semantic Segmentation"

(https://paperswithcode.com/paper/bisenet-bilateral-segmentation-network-for)

Args:

num_classes (int): The unique number of target classes.

backbone (paddle.nn.Layer): Backbone network, currently support Resnet18_vd/Resnet34_vd/Resnet50_vd/Resnet101_vd.

pretrained (str, optional): The path or url of pretrained model. Default: None.

"""

def __init__(self, num_classes, backbone, conv_channel=128,

pretrained=None):

super().__init__()

self.backbone = backbone

self.spatial_path = SpatialPath(3, 128)

self.global_context = nn.Sequential(

nn.AdaptiveAvgPool2D(1),

layers.ConvBNReLU(

512, conv_channel, 1, bias_attr=False), )

self.arms = nn.LayerList([

AttentionRefinement(512, conv_channel),

AttentionRefinement(256, conv_channel),

])

self.refines = nn.LayerList([

layers.ConvBNReLU(

conv_channel,

conv_channel,

3,

stride=1,

padding=1,

bias_attr=False),

layers.ConvBNReLU(

conv_channel,

conv_channel,

3,

stride=1,

padding=1,

bias_attr=False),

])

self.heads = nn.LayerList([

BiSeNetHead(conv_channel, num_classes, 8, True),

BiSeNetHead(conv_channel, num_classes, 8, True),

BiSeNetHead(conv_channel * 2, num_classes, 8, False),

])

self.ffm = FeatureFusion(conv_channel * 2, conv_channel * 2, 1)

self.pretrained = pretrained

def init_weight(self):

if self.pretrained is not None:

utils.load_entire_model(self, self.pretrained)

def forward(self, x):

spatial_out = self.spatial_path(x)

context_blocks = self.backbone(x)

context_blocks.reverse()

global_context = self.global_context(context_blocks[0])

global_context = F.interpolate(

global_context,

size=paddle.shape(context_blocks[0])[2:],

mode='bilinear',

align_corners=True)

last_fm = global_context

pred_out = []

for i, (

fm, arm, refine

) in enumerate(zip(context_blocks[:2], self.arms, self.refines)):

fm = arm(fm)

fm += last_fm

last_fm = F.interpolate(

fm,

size=paddle.shape(context_blocks[i + 1])[2:],

mode='bilinear',

align_corners=True)

last_fm = refine(last_fm)

pred_out.append(last_fm)

context_out = last_fm

concate_fm = self.ffm(spatial_out, context_out)

pred_out.append(concate_fm)

output = []

if self.training:

for i, head in enumerate(self.heads):

out = head(pred_out[i])

output.append(out)

else:

out = self.heads[-1](pred_out[-1])

output.append(out)

return output

class SpatialPath(nn.Layer):

"""

SpatialPath module of BiseNetV1 model

Args:

in_channels (int): The number of input channels in spatial path module.

out_channels (int): The number of output channels in spatial path module.

"""

def __init__(self, in_channels, out_channels, inner_channel=64):

super().__init__()

self.conv_7x7 = layers.ConvBNReLU(

in_channels, inner_channel, 7, stride=2, padding=3, bias_attr=False)

self.conv_3x3_1 = layers.ConvBNReLU(

inner_channel,

inner_channel,

3,

stride=2,

padding=1,

bias_attr=False)

self.conv_3x3_2 = layers.ConvBNReLU(

inner_channel,

inner_channel,

3,

stride=2,

padding=1,

bias_attr=False)

self.conv_1x1 = layers.ConvBNReLU(

inner_channel, out_channels, 1, bias_attr=False)

def forward(self, x):

x = self.conv_7x7(x)

x = self.conv_3x3_1(x)

x = self.conv_3x3_2(x)

x = self.conv_1x1(x)

return x

class BiSeNetHead(nn.Layer):

"""

BiSeNet head of BiseNetV1 model

Args:

in_channels (int): The number of input channels in spatial path module.

out_channels (int): The number of output channels in spatial path module.

scale (int, float): The scale factor of interpolation.

"""

def __init__(self, in_channels, out_channels, scale, is_aux=False):

super().__init__()

inner_channel = 128 if is_aux else 64

self.conv_3x3 = layers.ConvBNReLU(

in_channels, inner_channel, 3, stride=1, padding=1, bias_attr=False)

self.conv_1x1 = nn.Conv2D(inner_channel, out_channels, 1)

self.scale = scale

def forward(self, x):

x = self.conv_3x3(x)

x = self.conv_1x1(x)

if self.scale > 1:

x = F.interpolate(

x, scale_factor=self.scale, mode='bilinear', align_corners=True)

return x

class AttentionRefinement(nn.Layer):

"""

AttentionRefinement module of BiseNetV1 model

Args:

in_channels (int): The number of input channels in spatial path module.

out_channels (int): The number of output channels in spatial path module.

"""

def __init__(self, in_channels, out_channels):

super().__init__()

self.conv_3x3 = layers.ConvBNReLU(

in_channels, out_channels, 3, stride=1, padding=1, bias_attr=False)

self.channel_attention = nn.Sequential(

nn.AdaptiveAvgPool2D(1),

layers.ConvBNReLU(

out_channels, out_channels, 1, bias_attr=False),

nn.Sigmoid(), )

def forward(self, x):

x = self.conv_3x3(x)

se = self.channel_attention(x)

x = x * se

return x

class FeatureFusion(nn.Layer):

"""

AttentionRefinement module of BiseNetV1 model

Args:

in_channels (int): The number of input channels in spatial path module.

out_channels (int): The number of output channels in spatial path module.

reduction (int): A factor shrinks convolutional channels. Default: 1.

"""

def __init__(self, in_channels, out_channels, reduction=1):

super().__init__()

self.conv_1x1 = layers.ConvBNReLU(

in_channels, out_channels, 1, bias_attr=False)

self.channel_attention = nn.Sequential(

nn.AdaptiveAvgPool2D(1),

layers.ConvBNReLU(

out_channels, out_channels // reduction, 1, bias_attr=False),

layers.ConvBNReLU(

out_channels // reduction, out_channels, 1, bias_attr=False),

nn.Sigmoid(), )

def forward(self, x1, x2):

fm = paddle.concat([x1, x2], axis=1)

fm = self.conv_1x1(fm)

fm_se = self.channel_attention(fm)

output = fm + fm * fm_se

return output

import os

import paddle

import paddle.nn as nn

import paddle.nn.functional as F

from paddleseg import utils

from paddleseg.cvlibs import manager, param_init

from paddleseg.models import layers

@manager.MODELS.add_component

class BiSeNetV2(nn.Layer):

"""

The BiSeNet V2 implementation based on PaddlePaddle.

The original article refers to

Yu, Changqian, et al. "BiSeNet V2: Bilateral Network with Guided Aggregation for Real-time Semantic Segmentation"

(https://arxiv.org/abs/2004.02147)

Args:

num_classes (int): The unique number of target classes.

lambd (float, optional): A factor for controlling the size of semantic branch channels. Default: 0.25.

pretrained (str, optional): The path or url of pretrained model. Default: None.

"""

def __init__(self,

num_classes,

lambd=0.25,

align_corners=False,

pretrained=None):

super().__init__()

C1, C2, C3 = 64, 64, 128

db_channels = (C1, C2, C3)

C1, C3, C4, C5 = int(C1 * lambd), int(C3 * lambd), 64, 128

sb_channels = (C1, C3, C4, C5)

mid_channels = 128

self.db = DetailBranch(db_channels)

self.sb = SemanticBranch(sb_channels)

self.bga = BGA(mid_channels, align_corners)

self.aux_head1 = SegHead(C1, C1, num_classes)

self.aux_head2 = SegHead(C3, C3, num_classes)

self.aux_head3 = SegHead(C4, C4, num_classes)

self.aux_head4 = SegHead(C5, C5, num_classes)

self.head = SegHead(mid_channels, mid_channels, num_classes)

self.align_corners = align_corners

self.pretrained = pretrained

self.init_weight()

def forward(self, x):

dfm = self.db(x)

feat1, feat2, feat3, feat4, sfm = self.sb(x)

logit = self.head(self.bga(dfm, sfm))

if not self.training:

logit_list = [logit]

else:

logit1 = self.aux_head1(feat1)

logit2 = self.aux_head2(feat2)

logit3 = self.aux_head3(feat3)

logit4 = self.aux_head4(feat4)

logit_list = [logit, logit1, logit2, logit3, logit4]

logit_list = [

F.interpolate(

logit,

paddle.shape(x)[2:],

mode='bilinear',

align_corners=self.align_corners) for logit in logit_list

]

return logit_list

def init_weight(self):

if self.pretrained is not None:

utils.load_entire_model(self, self.pretrained)

else:

for sublayer in self.sublayers():

if isinstance(sublayer, nn.Conv2D):

param_init.kaiming_normal_init(sublayer.weight)

elif isinstance(sublayer, (nn.BatchNorm, nn.SyncBatchNorm)):

param_init.constant_init(sublayer.weight, value=1.0)

param_init.constant_init(sublayer.bias, value=0.0)

class StemBlock(nn.Layer):

def __init__(self, in_dim, out_dim):

super(StemBlock, self).__init__()

self.conv = layers.ConvBNReLU(in_dim, out_dim, 3, stride=2)

self.left = nn.Sequential(

layers.ConvBNReLU(out_dim, out_dim // 2, 1),

layers.ConvBNReLU(

out_dim // 2, out_dim, 3, stride=2))

self.right = nn.MaxPool2D(kernel_size=3, stride=2, padding=1)

self.fuse = layers.ConvBNReLU(out_dim * 2, out_dim, 3)

def forward(self, x):

x = self.conv(x)

left = self.left(x)

right = self.right(x)

concat = paddle.concat([left, right], axis=1)

return self.fuse(concat)

class ContextEmbeddingBlock(nn.Layer):

def __init__(self, in_dim, out_dim):

super(ContextEmbeddingBlock, self).__init__()

self.gap = nn.AdaptiveAvgPool2D(1)

self.bn = layers.SyncBatchNorm(in_dim)

self.conv_1x1 = layers.ConvBNReLU(in_dim, out_dim, 1)

self.add = layers.Add()

self.conv_3x3 = nn.Conv2D(out_dim, out_dim, 3, 1, 1)

def forward(self, x):

gap = self.gap(x)

bn = self.bn(gap)

conv1 = self.add(self.conv_1x1(bn), x)

return self.conv_3x3(conv1)

class GatherAndExpansionLayer1(nn.Layer):

"""Gather And Expansion Layer with stride 1"""

def __init__(self, in_dim, out_dim, expand):

super().__init__()

expand_dim = expand * in_dim

self.conv = nn.Sequential(

layers.ConvBNReLU(in_dim, in_dim, 3),

layers.DepthwiseConvBN(in_dim, expand_dim, 3),

layers.ConvBN(expand_dim, out_dim, 1))

self.relu = layers.Activation("relu")

def forward(self, x):

return self.relu(self.conv(x) + x)

class GatherAndExpansionLayer2(nn.Layer):

"""Gather And Expansion Layer with stride 2"""

def __init__(self, in_dim, out_dim, expand):

super().__init__()

expand_dim = expand * in_dim

self.branch_1 = nn.Sequential(

layers.ConvBNReLU(in_dim, in_dim, 3),

layers.DepthwiseConvBN(

in_dim, expand_dim, 3, stride=2),

layers.DepthwiseConvBN(expand_dim, expand_dim, 3),

layers.ConvBN(expand_dim, out_dim, 1))

self.branch_2 = nn.Sequential(

layers.DepthwiseConvBN(

in_dim, in_dim, 3, stride=2),

layers.ConvBN(in_dim, out_dim, 1))

self.relu = layers.Activation("relu")

def forward(self, x):

return self.relu(self.branch_1(x) + self.branch_2(x))

class DetailBranch(nn.Layer):

"""The detail branch of BiSeNet, which has wide channels but shallow layers."""

def __init__(self, in_channels):

super().__init__()

C1, C2, C3 = in_channels

self.convs = nn.Sequential(

\# stage 1

layers.ConvBNReLU(

3, C1, 3, stride=2),

layers.ConvBNReLU(C1, C1, 3),

\# stage 2

layers.ConvBNReLU(

C1, C2, 3, stride=2),

layers.ConvBNReLU(C2, C2, 3),

layers.ConvBNReLU(C2, C2, 3),

\# stage 3

layers.ConvBNReLU(

C2, C3, 3, stride=2),

layers.ConvBNReLU(C3, C3, 3),

layers.ConvBNReLU(C3, C3, 3), )

def forward(self, x):

return self.convs(x)

class SemanticBranch(nn.Layer):

"""The semantic branch of BiSeNet, which has narrow channels but deep layers."""

def __init__(self, in_channels):

super().__init__()

C1, C3, C4, C5 = in_channels

self.stem = StemBlock(3, C1)

self.stage3 = nn.Sequential(

GatherAndExpansionLayer2(C1, C3, 6),

GatherAndExpansionLayer1(C3, C3, 6))

self.stage4 = nn.Sequential(

GatherAndExpansionLayer2(C3, C4, 6),

GatherAndExpansionLayer1(C4, C4, 6))

self.stage5_4 = nn.Sequential(

GatherAndExpansionLayer2(C4, C5, 6),

GatherAndExpansionLayer1(C5, C5, 6),

GatherAndExpansionLayer1(C5, C5, 6),

GatherAndExpansionLayer1(C5, C5, 6))

self.ce = ContextEmbeddingBlock(C5, C5)

def forward(self, x):

stage2 = self.stem(x)

stage3 = self.stage3(stage2)

stage4 = self.stage4(stage3)

stage5_4 = self.stage5_4(stage4)

fm = self.ce(stage5_4)

return stage2, stage3, stage4, stage5_4, fm

class BGA(nn.Layer):

"""The Bilateral Guided Aggregation Layer, used to fuse the semantic features and spatial features."""

def __init__(self, out_dim, align_corners):

super().__init__()

self.align_corners = align_corners

self.db_branch_keep = nn.Sequential(

layers.DepthwiseConvBN(out_dim, out_dim, 3),

nn.Conv2D(out_dim, out_dim, 1))

self.db_branch_down = nn.Sequential(

layers.ConvBN(

out_dim, out_dim, 3, stride=2),

nn.AvgPool2D(

kernel_size=3, stride=2, padding=1))

self.sb_branch_keep = nn.Sequential(

layers.DepthwiseConvBN(out_dim, out_dim, 3),

nn.Conv2D(out_dim, out_dim, 1),

layers.Activation(act='sigmoid'))

self.sb_branch_up = layers.ConvBN(out_dim, out_dim, 3)

self.conv = layers.ConvBN(out_dim, out_dim, 3)

def forward(self, dfm, sfm):

db_feat_keep = self.db_branch_keep(dfm)

db_feat_down = self.db_branch_down(dfm)

sb_feat_keep = self.sb_branch_keep(sfm)

sb_feat_up = self.sb_branch_up(sfm)

sb_feat_up = F.interpolate(

sb_feat_up,

paddle.shape(db_feat_keep)[2:],

mode='bilinear',

align_corners=self.align_corners)

sb_feat_up = F.sigmoid(sb_feat_up)

db_feat = db_feat_keep * sb_feat_up

sb_feat = db_feat_down * sb_feat_keep

sb_feat = F.interpolate(

sb_feat,

paddle.shape(db_feat)[2:],

mode='bilinear',

align_corners=self.align_corners)

return self.conv(db_feat + sb_feat)

class SegHead(nn.Layer):

def __init__(self, in_dim, mid_dim, num_classes):

super().__init__()

self.conv_3x3 = nn.Sequential(

layers.ConvBNReLU(in_dim, mid_dim, 3), nn.Dropout(0.1))

self.conv_1x1 = nn.Conv2D(mid_dim, num_classes, 1, 1)

def forward(self, x):

conv1 = self.conv_3x3(x)

conv2 = self.conv_1x1(conv1)

return conv2

import paddle

import paddle.nn as nn

from paddleseg.cvlibs import manager

from paddleseg.models import layers

from paddleseg import utils

import numpy as np

@manager.MODELS.add_component

class AttentionUNet(nn.Layer):

"""

The Attention-UNet implementation based on PaddlePaddle.

As mentioned in the original paper, author proposes a novel attention gate (AG)

that automatically learns to focus on target structures of varying shapes and sizes.

Models trained with AGs implicitly learn to suppress irrelevant regions in an input image while

highlighting salient features useful for a specific task.

The original article refers to

Oktay, O, et, al. "Attention u-net: Learning where to look for the pancreas."

(https://arxiv.org/pdf/1804.03999.pdf).

Args:

num_classes (int): The unique number of target classes.

pretrained (str, optional): The path or url of pretrained model. Default: None.

"""

def __init__(self, num_classes, pretrained=None):

super().__init__()

n_channels = 3

self.encoder = Encoder(n_channels, [64, 128, 256, 512])

filters = np.array([64, 128, 256, 512, 1024])

self.up5 = UpConv(ch_in=filters[4], ch_out=filters[3])

self.att5 = AttentionBlock(

F_g=filters[3], F_l=filters[3], F_out=filters[2])

self.up_conv5 = ConvBlock(ch_in=filters[4], ch_out=filters[3])

self.up4 = UpConv(ch_in=filters[3], ch_out=filters[2])

self.att4 = AttentionBlock(

F_g=filters[2], F_l=filters[2], F_out=filters[1])

self.up_conv4 = ConvBlock(ch_in=filters[3], ch_out=filters[2])

self.up3 = UpConv(ch_in=filters[2], ch_out=filters[1])

self.att3 = AttentionBlock(

F_g=filters[1], F_l=filters[1], F_out=filters[0])

self.up_conv3 = ConvBlock(ch_in=filters[2], ch_out=filters[1])

self.up2 = UpConv(ch_in=filters[1], ch_out=filters[0])

self.att2 = AttentionBlock(

F_g=filters[0], F_l=filters[0], F_out=filters[0] // 2)

self.up_conv2 = ConvBlock(ch_in=filters[1], ch_out=filters[0])

self.conv_1x1 = nn.Conv2D(

filters[0], num_classes, kernel_size=1, stride=1, padding=0)

self.pretrained = pretrained

self.init_weight()

def forward(self, x):

x5, (x1, x2, x3, x4) = self.encoder(x)

d5 = self.up5(x5)

x4 = self.att5(g=d5, x=x4)

d5 = paddle.concat([x4, d5], axis=1)

d5 = self.up_conv5(d5)

d4 = self.up4(d5)

x3 = self.att4(g=d4, x=x3)

d4 = paddle.concat((x3, d4), axis=1)

d4 = self.up_conv4(d4)

d3 = self.up3(d4)

x2 = self.att3(g=d3, x=x2)

d3 = paddle.concat((x2, d3), axis=1)

d3 = self.up_conv3(d3)

d2 = self.up2(d3)

x1 = self.att2(g=d2, x=x1)

d2 = paddle.concat((x1, d2), axis=1)

d2 = self.up_conv2(d2)

logit = self.conv_1x1(d2)

logit_list = [logit]

return logit_list

def init_weight(self):

if self.pretrained is not None:

utils.load_entire_model(self, self.pretrained)

class AttentionBlock(nn.Layer):

def __init__(self, F_g, F_l, F_out):

super().__init__()

self.W_g = nn.Sequential(

nn.Conv2D(

F_g, F_out, kernel_size=1, stride=1, padding=0),

nn.BatchNorm2D(F_out))

self.W_x = nn.Sequential(

nn.Conv2D(

F_l, F_out, kernel_size=1, stride=1, padding=0),

nn.BatchNorm2D(F_out))

self.psi = nn.Sequential(

nn.Conv2D(

F_out, 1, kernel_size=1, stride=1, padding=0),

nn.BatchNorm2D(1),

nn.Sigmoid())

self.relu = nn.ReLU()

def forward(self, g, x):

g1 = self.W_g(g)

x1 = self.W_x(x)

psi = self.relu(g1 + x1)

psi = self.psi(psi)

res = x * psi

return res

class UpConv(nn.Layer):

def __init__(self, ch_in, ch_out):

super().__init__()

self.up = nn.Sequential(

nn.Upsample(

scale_factor=2, mode="bilinear"),

nn.Conv2D(

ch_in, ch_out, kernel_size=3, stride=1, padding=1),

nn.BatchNorm2D(ch_out),

nn.ReLU())

def forward(self, x):

return self.up(x)

class Encoder(nn.Layer):

def __init__(self, input_channels, filters):

super().__init__()

self.double_conv = nn.Sequential(

layers.ConvBNReLU(input_channels, 64, 3),

layers.ConvBNReLU(64, 64, 3))

down_channels = filters

self.down_sample_list = nn.LayerList([

self.down_sampling(channel, channel * 2)

for channel in down_channels

])

def down_sampling(self, in_channels, out_channels):

modules = []

modules.append(nn.MaxPool2D(kernel_size=2, stride=2))

modules.append(layers.ConvBNReLU(in_channels, out_channels, 3))

modules.append(layers.ConvBNReLU(out_channels, out_channels, 3))

return nn.Sequential(*modules)

def forward(self, x):

short_cuts = []

x = self.double_conv(x)

for down_sample in self.down_sample_list:

short_cuts.append(x)

x = down_sample(x)

return x, short_cuts

class ConvBlock(nn.Layer):

def __init__(self, ch_in, ch_out):

super(ConvBlock, self).__init__()

self.conv = nn.Sequential(

nn.Conv2D(

ch_in, ch_out, kernel_size=3, stride=1, padding=1),

nn.BatchNorm2D(ch_out),

nn.ReLU(),

nn.Conv2D(

ch_out, ch_out, kernel_size=3, stride=1, padding=1),

nn.BatchNorm2D(ch_out),

nn.ReLU())

def forward(self, x):

return self.conv(x)

import os

import numpy as np

from PIL import Image

from paddleseg.datasets import Dataset

from paddleseg.utils.download import download_file_and_uncompress

from paddleseg.utils import seg_env

from paddleseg.cvlibs import manager

from paddleseg.transforms import Compose

import paddleseg.transforms.functional as F

URL = "http://data.csail.mit.edu/places/ADEchallenge/ADEChallengeData2016.zip"

@manager.DATASETS.add_component

class ADE20K(Dataset):

"""

ADE20K dataset `http://sceneparsing.csail.mit.edu/`.

Args:

transforms (list): A list of image transformations.

dataset_root (str, optional): The ADK20K dataset directory. Default: None.

mode (str, optional): A subset of the entire dataset. It should be one of ('train', 'val'). Default: 'train'.

edge (bool, optional): Whether to compute edge while training. Default: False

"""

NUM_CLASSES = 150

def __init__(self, transforms, dataset_root=None, mode='train', edge=False):

self.dataset_root = dataset_root

self.transforms = Compose(transforms)

mode = mode.lower()

self.mode = mode

self.file_list = list()

self.num_classes = self.NUM_CLASSES

self.ignore_index = 255

self.edge = edge

if mode not in ['train', 'val']:

raise ValueError(

"`mode` should be one of ('train', 'val') in ADE20K dataset, but got {}."

.format(mode))

if self.transforms is None:

raise ValueError("`transforms` is necessary, but it is None.")

if self.dataset_root is None:

self.dataset_root = download_file_and_uncompress(

url=URL,

savepath=seg_env.DATA_HOME,

extrapath=seg_env.DATA_HOME,

extraname='ADEChallengeData2016')

elif not os.path.exists(self.dataset_root):

self.dataset_root = os.path.normpath(self.dataset_root)

savepath, extraname = self.dataset_root.rsplit(

sep=os.path.sep, maxsplit=1)

self.dataset_root = download_file_and_uncompress(

url=URL,

savepath=savepath,

extrapath=savepath,

extraname=extraname)

if mode == 'train':

img_dir = os.path.join(self.dataset_root, 'images/training')

label_dir = os.path.join(self.dataset_root, 'annotations/training')

elif mode == 'val':

img_dir = os.path.join(self.dataset_root, 'images/validation')

label_dir = os.path.join(self.dataset_root,

'annotations/validation')

img_files = os.listdir(img_dir)

label_files = [i.replace('.jpg', '.png') for i in img_files]

for i in range(len(img_files)):

img_path = os.path.join(img_dir, img_files[i])

label_path = os.path.join(label_dir, label_files[i])

self.file_list.append([img_path, label_path])

def __getitem__(self, idx):

data = {}

data['trans_info'] = []

image_path, label_path = self.file_list[idx]

data['img'] = image_path

data['gt_fields'] = [

] # If key in gt_fields, the data[key] have transforms synchronous.

if self.mode == 'val':

data = self.transforms(data)

label = np.asarray(Image.open(label_path))

\# The class 0 is ignored. And it will equal to 255 after

\# subtracted 1, because the dtype of label is uint8.

label = label - 1

label = label[np.newaxis, :, :]

data['label'] = label

return data

else:

data['label'] = label_path

data['gt_fields'].append('label')

data = self.transforms(data)

data['label'] = data['label'] - 1

\# Recover the ignore pixels adding by transform

data['label'][data['label'] == 254] = 255

if self.edge:

edge_mask = F.mask_to_binary_edge(

label, radius=2, num_classes=self.num_classes)

data['edge'] = edge_mask

return data

import paddle.nn as nn

def constant_init(param, **kwargs):

"""

Initialize the `param` with constants.

Args:

param (Tensor): Tensor that needs to be initialized.

Examples:

from paddleseg.cvlibs import param_init

import paddle.nn as nn

linear = nn.Linear(2, 4)

param_init.constant_init(linear.weight, value=2.0)

print(linear.weight.numpy())

\# result is [[2. 2. 2. 2.], [2. 2. 2. 2.]]

"""

initializer = nn.initializer.Constant(**kwargs)

initializer(param, param.block)

def normal_init(param, **kwargs):

"""

Initialize the `param` with a Normal distribution.

Args:

param (Tensor): Tensor that needs to be initialized.

Examples:

from paddleseg.cvlibs import param_init

import paddle.nn as nn

linear = nn.Linear(2, 4)

param_init.normal_init(linear.weight, loc=0.0, scale=1.0)

"""

initializer = nn.initializer.Normal(**kwargs)

initializer(param, param.block)

def kaiming_normal_init(param, **kwargs):

r"""

Initialize the input tensor with Kaiming Normal initialization.

This function implements the `param` initialization from the paper

`Delving Deep into Rectifiers: Surpassing Human-Level Performance on

ImageNet Classification <https://arxiv.org/abs/1502.01852>`

by Kaiming He, Xiangyu Zhang, Shaoqing Ren and Jian Sun. This is a

robust initialization method that particularly considers the rectifier

nonlinearities. In case of Uniform distribution, the range is [-x, x], where

.. math::

x = \sqrt{\\frac{6.0}{fan\_in}}

In case of Normal distribution, the mean is 0 and the standard deviation

is

.. math::

\sqrt{\\frac{2.0}{fan\_in}}

Args:

param (Tensor): Tensor that needs to be initialized.

Examples:

from paddleseg.cvlibs import param_init

import paddle.nn as nn

linear = nn.Linear(2, 4)

\# uniform is used to decide whether to use uniform or normal distribution

param_init.kaiming_normal_init(linear.weight)

"""

initializer = nn.initializer.KaimingNormal(**kwargs)

initializer(param, param.block)

def kaiming_uniform(param, **kwargs):

r"""Implements the Kaiming Uniform initializer

This class implements the weight initialization from the paper

`Delving Deep into Rectifiers: Surpassing Human-Level Performance on

ImageNet Classification <https://arxiv.org/abs/1502.01852>`_

by Kaiming He, Xiangyu Zhang, Shaoqing Ren and Jian Sun. This is a

robust initialization method that particularly considers the rectifier

nonlinearities.

In case of Uniform distribution, the range is [-x, x], where

.. math::

x = \sqrt{\\frac{6.0}{fan\_in}}

Args:

param (Tensor): Tensor that needs to be initialized.

Examples:

from paddleseg.cvlibs import param_init

import paddle.nn as nn

linear = nn.Linear(2, 4)

param_init.kaiming_uniform(linear.weight)

"""

initializer = nn.initializer.KaimingUniform(**kwargs)

initializer(param, param.block)

def xavier_uniform(param, **kwargs):

r"""

This implements the Xavier weight initializer from the paper

`Understanding the difficulty of training deep feedforward neural

networks <http://proceedings.mlr.press/v9/glorot10a/glorot10a.pdf>`_

by Xavier Glorot and Yoshua Bengio.

This initializer is designed to keep the scale of the gradients

approximately same in all the layers. In case of Uniform distribution,

the range is [-x, x], where

.. math::

x = \sqrt{\frac{6.0}{fan\_in + fan\_out}}

Args:

param (Tensor): Tensor that needs to be initialized.

Examples:

from paddleseg.cvlibs import param_init

import paddle.nn as nn

linear = nn.Linear(2, 4)

param_init.xavier_uniform(linear.weight)

"""

initializer = nn.initializer.XavierUniform(**kwargs)

initializer(param, param.block)

import inspect

from collections.abc import Sequence

import warnings

class ComponentManager:

"""

Implement a manager class to add the new component properly.

The component can be added as either class or function type.

Args:

name (str): The name of component.

Returns:

A callable object of ComponentManager.

Examples 1:

from paddleseg.cvlibs.manager import ComponentManager

model_manager = ComponentManager()

class AlexNet: ...

class ResNet: ...

model_manager.add_component(AlexNet)

model_manager.add_component(ResNet)

\# Or pass a sequence alliteratively:

model_manager.add_component([AlexNet, ResNet])

print(model_manager.components_dict)

\# {'AlexNet': <class '__main__.AlexNet'>, 'ResNet': <class '__main__.ResNet'>}

Examples 2:

\# Or an easier way, using it as a Python decorator, while just add it above the class declaration.

from paddleseg.cvlibs.manager import ComponentManager

model_manager = ComponentManager()

@model_manager.add_component

class AlexNet: ...

@model_manager.add_component

class ResNet: ...

print(model_manager.components_dict)

\# {'AlexNet': <class '__main__.AlexNet'>, 'ResNet': <class '__main__.ResNet'>}

"""

def __init__(self, name=None):

self._components_dict = dict()

self._name = name

def __len__(self):

return len(self._components_dict)

def __repr__(self):

name_str = self._name if self._name else self.__class__.__name__

return "{}:{}".format(name_str, list(self._components_dict.keys()))

def __getitem__(self, item):

if item not in self._components_dict.keys():

raise KeyError("{} does not exist in availabel {}".format(item,

self))

return self._components_dict[item]

@property

def components_dict(self):

return self._components_dict

@property

def name(self):

return self._name

def _add_single_component(self, component):

"""

Add a single component into the corresponding manager.

Args:

component (function|class): A new component.

Raises:

TypeError: When `component` is neither class nor function.

KeyError: When `component` was added already.

"""

\# Currently only support class or function type

if not (inspect.isclass(component) or inspect.isfunction(component)):

raise TypeError("Expect class/function type, but received {}".

format(type(component)))

\# Obtain the internal name of the component

component_name = component.__name__

\# Check whether the component was added already

if component_name in self._components_dict.keys():

warnings.warn("{} exists already! It is now updated to {} !!!".

format(component_name, component))

self._components_dict[component_name] = component

else:

\# Take the internal name of the component as its key

self._components_dict[component_name] = component

def add_component(self, components):

"""

Add component(s) into the corresponding manager.

Args:

components (function|class|list|tuple): Support four types of components.

Returns:

components (function|class|list|tuple): Same with input components.

"""

\# Check whether the type is a sequence

if isinstance(components, Sequence):

for component in components:

self._add_single_component(component)

else:

component = components

self._add_single_component(component)

return components

MODELS = ComponentManager("models")

BACKBONES = ComponentManager("backbones")

DATASETS = ComponentManager("datasets")

TRANSFORMS = ComponentManager("transforms")

LOSSES = ComponentManager("losses")

import codecs

import os

from typing import Any, Dict, Generic

import warnings

import paddle

import yaml

from paddleseg.cvlibs import manager

from paddleseg.utils import logger

class Config(object):

'''

Training configuration parsing. The only yaml/yml file is supported.

The following hyper-parameters are available in the config file:

batch_size: The number of samples per gpu.

iters: The total training steps.

train_dataset: A training data config including type/data_root/transforms/mode.

For data type, please refer to paddleseg.datasets.

For specific transforms, please refer to paddleseg.transforms.transforms.

val_dataset: A validation data config including type/data_root/transforms/mode.

optimizer: A optimizer config, but currently PaddleSeg only supports sgd with momentum in config file.

In addition, weight_decay could be set as a regularization.

learning_rate: A learning rate config. If decay is configured, learning _rate value is the starting learning rate,

where only poly decay is supported using the config file. In addition, decay power and end_lr are tuned experimentally.

loss: A loss config. Multi-loss config is available. The loss type order is consistent with the seg model outputs,

where the coef term indicates the weight of corresponding loss. Note that the number of coef must be the same as the number of

model outputs, and there could be only one loss type if using the same loss type among the outputs, otherwise the number of

loss type must be consistent with coef.

model: A model config including type/backbone and model-dependent arguments.

For model type, please refer to paddleseg.models.

For backbone, please refer to paddleseg.models.backbones.

Args:

path (str) : The path of config file, supports yaml format only.

Examples:

from paddleseg.cvlibs.config import Config

\# Create a cfg object with yaml file path.

cfg = Config(yaml_cfg_path)

\# Parsing the argument when its property is used.

train_dataset = cfg.train_dataset

\# the argument of model should be parsed after dataset,

\# since the model builder uses some properties in dataset.

model = cfg.model

...

'''

def __init__(self,

path: str,

learning_rate: float=None,

batch_size: int=None,

iters: int=None):

if not path:

raise ValueError('Please specify the configuration file path.')

if not os.path.exists(path):

raise FileNotFoundError('File {} does not exist'.format(path))

self._model = None

self._losses = None

if path.endswith('yml') or path.endswith('yaml'):

self.dic = self._parse_from_yaml(path)

else:

raise RuntimeError('Config file should in yaml format!')

self.update(

learning_rate=learning_rate, batch_size=batch_size, iters=iters)

def _update_dic(self, dic, base_dic):

"""

Update config from dic based base_dic

"""

base_dic = base_dic.copy()

dic = dic.copy()

if dic.get('_inherited_', True) == False:

dic.pop('_inherited_')

return dic

for key, val in dic.items():

if isinstance(val, dict) and key in base_dic:

base_dic[key] = self._update_dic(val, base_dic[key])

else:

base_dic[key] = val

dic = base_dic

return dic

def _parse_from_yaml(self, path: str):

'''Parse a yaml file and build config'''

with codecs.open(path, 'r', 'utf-8') as file:

dic = yaml.load(file, Loader=yaml.FullLoader)

if '_base_' in dic:

cfg_dir = os.path.dirname(path)

base_path = dic.pop('_base_')

base_path = os.path.join(cfg_dir, base_path)

base_dic = self._parse_from_yaml(base_path)

dic = self._update_dic(dic, base_dic)

return dic

def update(self,

learning_rate: float=None,

batch_size: int=None,

iters: int=None):

'''Update config'''

if learning_rate:

if 'lr_scheduler' in self.dic:

self.dic['lr_scheduler']['learning_rate'] = learning_rate

else:

self.dic['learning_rate']['value'] = learning_rate

if batch_size:

self.dic['batch_size'] = batch_size

if iters:

self.dic['iters'] = iters

@property

def batch_size(self) -> int:

return self.dic.get('batch_size', 1)

@property

def iters(self) -> int:

iters = self.dic.get('iters')

if not iters:

raise RuntimeError('No iters specified in the configuration file.')

return iters

@property

def lr_scheduler(self) -> paddle.optimizer.lr.LRScheduler:

if 'lr_scheduler' not in self.dic:

raise RuntimeError(

'No `lr_scheduler` specified in the configuration file.')

params = self.dic.get('lr_scheduler')

use_warmup = False

if 'warmup_iters' in params:

use_warmup = True

warmup_iters = params.pop('warmup_iters')

assert 'warmup_start_lr' in params, \

"When use warmup, please set warmup_start_lr and warmup_iters in lr_scheduler"

warmup_start_lr = params.pop('warmup_start_lr')

end_lr = params['learning_rate']

lr_type = params.pop('type')

if lr_type == 'PolynomialDecay':

iters = self.iters - warmup_iters if use_warmup else self.iters

iters = max(iters, 1)

params.setdefault('decay_steps', iters)

params.setdefault('end_lr', 0)

params.setdefault('power', 0.9)

lr_sche = getattr(paddle.optimizer.lr, lr_type)(**params)

if use_warmup:

lr_sche = paddle.optimizer.lr.LinearWarmup(

learning_rate=lr_sche,

warmup_steps=warmup_iters,

start_lr=warmup_start_lr,

end_lr=end_lr)

return lr_sche

@property

def learning_rate(self) -> paddle.optimizer.lr.LRScheduler:

logger.warning(

'''`learning_rate` in configuration file will be deprecated, please use `lr_scheduler` instead. E.g

lr_scheduler:

type: PolynomialDecay

learning_rate: 0.01''')

_learning_rate = self.dic.get('learning_rate', {})

if isinstance(_learning_rate, float):

return _learning_rate

_learning_rate = self.dic.get('learning_rate', {}).get('value')

if not _learning_rate:

raise RuntimeError(

'No learning rate specified in the configuration file.')

args = self.decay_args

decay_type = args.pop('type')

if decay_type == 'poly':

lr = _learning_rate

return paddle.optimizer.lr.PolynomialDecay(lr, **args)

elif decay_type == 'piecewise':

values = _learning_rate

return paddle.optimizer.lr.PiecewiseDecay(values=values, **args)

elif decay_type == 'stepdecay':

lr = _learning_rate

return paddle.optimizer.lr.StepDecay(lr, **args)

else:

raise RuntimeError('Only poly and piecewise decay support.')

@property

def optimizer(self) -> paddle.optimizer.Optimizer:

if 'lr_scheduler' in self.dic:

lr = self.lr_scheduler

else:

lr = self.learning_rate

args = self.optimizer_args

optimizer_type = args.pop('type')

if optimizer_type == 'sgd':

return paddle.optimizer.Momentum(

lr, parameters=self.model.parameters(), **args)

elif optimizer_type == 'adam':

return paddle.optimizer.Adam(

lr, parameters=self.model.parameters(), **args)

elif optimizer_type in paddle.optimizer.__all__:

return getattr(paddle.optimizer,

optimizer_type)(lr,

parameters=self.model.parameters(),

**args)

raise RuntimeError('Unknown optimizer type {}.'.format(optimizer_type))

@property

def optimizer_args(self) -> dict:

args = self.dic.get('optimizer', {}).copy()

if args['type'] == 'sgd':

args.setdefault('momentum', 0.9)

return args

@property

def decay_args(self) -> dict:

args = self.dic.get('learning_rate', {}).get(

'decay', {'type': 'poly',

'power': 0.9}).copy()

if args['type'] == 'poly':

args.setdefault('decay_steps', self.iters)

args.setdefault('end_lr', 0)

return args

@property

def loss(self) -> dict:

if self._losses is None:

self._losses = self._prepare_loss('loss')

return self._losses

@property

def distill_loss(self) -> dict:

if not hasattr(self, '_distill_losses'):

self._distill_losses = self._prepare_loss('distill_loss')

return self._distill_losses

def _prepare_loss(self, loss_name):

"""

Parse the loss parameters and load the loss layers.

Args:

loss_name (str): The root name of loss in the yaml file.

Returns:

dict: A dict including the loss parameters and layers.

"""

args = self.dic.get(loss_name, {}).copy()

if 'types' in args and 'coef' in args:

len_types = len(args['types'])

len_coef = len(args['coef'])

if len_types != len_coef:

if len_types == 1:

args['types'] = args['types'] * len_coef

else:

raise ValueError(

'The length of types should equal to coef or equal to 1 in loss config, but they are {} and {}.'

.format(len_types, len_coef))

else:

raise ValueError(

'Loss config should contain keys of "types" and "coef"')

losses = dict()

for key, val in args.items():

if key == 'types':

losses['types'] = []

for item in args['types']:

if item['type'] != 'MixedLoss':

if 'ignore_index' in item:

assert item['ignore_index'] == self.train_dataset.ignore_index, 'If ignore_index of loss is set, '\

'the ignore_index of loss and train_dataset must be the same. \nCurrently, loss ignore_index = {}, '\

'train_dataset ignore_index = {}. \nIt is recommended not to set loss ignore_index, so it is consistent with '\

'train_dataset by default.'.format(item['ignore_index'], self.train_dataset.ignore_index)

item['ignore_index'] = \

self.train_dataset.ignore_index

losses['types'].append(self._load_object(item))

else:

losses[key] = val

if len(losses['coef']) != len(losses['types']):

raise RuntimeError(

'The length of coef should equal to types in loss config: {} != {}.'

.format(len(losses['coef']), len(losses['types'])))

return losses

@property

def model(self) -> paddle.nn.Layer:

model_cfg = self.dic.get('model').copy()

if not model_cfg:

raise RuntimeError('No model specified in the configuration file.')

if not 'num_classes' in model_cfg:

num_classes = None

try:

if self.train_dataset_config:

if hasattr(self.train_dataset_class, 'NUM_CLASSES'):

num_classes = self.train_dataset_class.NUM_CLASSES

elif 'num_classes' in self.train_dataset_config:

num_classes = self.train_dataset_config['num_classes']

elif hasattr(self.train_dataset, 'num_classes'):

num_classes = self.train_dataset.num_classes

elif self.val_dataset_config:

if hasattr(self.val_dataset_class, 'NUM_CLASSES'):

num_classes = self.val_dataset_class.NUM_CLASSES

elif 'num_classes' in self.val_dataset_config:

num_classes = self.val_dataset_config['num_classes']

elif hasattr(self.val_dataset, 'num_classes'):

num_classes = self.val_dataset.num_classes

except FileNotFoundError:

warnings.warn("`dataset_root` is not found. Is it correct?")

if num_classes is not None:

model_cfg['num_classes'] = num_classes

if not self._model:

self._model = self._load_object(model_cfg)

return self._model

@property

def train_dataset_config(self) -> Dict:

return self.dic.get('train_dataset', {}).copy()

@property

def val_dataset_config(self) -> Dict:

return self.dic.get('val_dataset', {}).copy()

@property

def train_dataset_class(self) -> Generic:

dataset_type = self.train_dataset_config['type']

return self._load_component(dataset_type)

@property

def val_dataset_class(self) -> Generic:

dataset_type = self.val_dataset_config['type']

return self._load_component(dataset_type)

@property

def train_dataset(self) -> paddle.io.Dataset:

_train_dataset = self.train_dataset_config

if not _train_dataset:

return None

return self._load_object(_train_dataset)

@property

def val_dataset(self) -> paddle.io.Dataset:

_val_dataset = self.val_dataset_config

if not _val_dataset:

return None

return self._load_object(_val_dataset)

def _load_component(self, com_name: str) -> Any:

com_list = [

manager.MODELS, manager.BACKBONES, manager.DATASETS,

manager.TRANSFORMS, manager.LOSSES

]

for com in com_list:

if com_name in com.components_dict:

return com[com_name]

else:

raise RuntimeError(

'The specified component was not found {}.'.format(com_name))

def _load_object(self, cfg: dict) -> Any:

cfg = cfg.copy()

if 'type' not in cfg:

raise RuntimeError('No object information in {}.'.format(cfg))

component = self._load_component(cfg.pop('type'))

params = {}

for key, val in cfg.items():

if self._is_meta_type(val):

params[key] = self._load_object(val)

elif isinstance(val, list):

params[key] = [

self._load_object(item)

if self._is_meta_type(item) else item for item in val

]

else:

params[key] = val

return component(**params)

@property

def test_config(self) -> Dict:

return self.dic.get('test_config', {})

@property

def export_config(self) -> Dict:

return self.dic.get('export', {})

@property

def to_static_training(self) -> bool:

'''Whether to use @to_static for training'''

return self.dic.get('to_static_training', False)

def _is_meta_type(self, item: Any) -> bool:

return isinstance(item, dict) and 'type' in item

def __str__(self) -> str:

return yaml.dump(self.dic)

@property

def val_transforms(self) -> list:

"""Get val_transform from val_dataset"""

_val_dataset = self.val_dataset_config

if not _val_dataset:

return []

_transforms = _val_dataset.get('transforms', [])

transforms = []

for i in _transforms:

transforms.append(self._load_object(i))

return transforms

import os

import time

import numpy as np

import paddle

from paddle.distributed.parallel import ParallelEnv

from visualdl import LogWriter

from paddleseg.utils.progbar import Progbar

import paddleseg.utils.logger as logger

class CallbackList(object):

"""

Container abstracting a list of callbacks.

Args:

callbacks (list[Callback]): List of `Callback` instances.

"""

def __init__(self, callbacks=None):

callbacks = callbacks or []

self.callbacks = [c for c in callbacks]

def append(self, callback):

self.callbacks.append(callback)

def set_params(self, params):

for callback in self.callbacks:

callback.set_params(params)

def set_model(self, model):

for callback in self.callbacks:

callback.set_model(model)

def set_optimizer(self, optimizer):

for callback in self.callbacks:

callback.set_optimizer(optimizer)

def on_iter_begin(self, iter, logs=None):

"""Called right before processing a batch.

"""

logs = logs or {}

for callback in self.callbacks:

callback.on_iter_begin(iter, logs)

self._t_enter_iter = time.time()

def on_iter_end(self, iter, logs=None):

"""Called at the end of a batch.

"""

logs = logs or {}

for callback in self.callbacks:

callback.on_iter_end(iter, logs)

self._t_exit_iter = time.time()

def on_train_begin(self, logs=None):

"""Called at the beginning of training.

"""

logs = logs or {}

for callback in self.callbacks:

callback.on_train_begin(logs)

def on_train_end(self, logs=None):

"""Called at the end of training.

"""

logs = logs or {}

for callback in self.callbacks:

callback.on_train_end(logs)

def __iter__(self):

return iter(self.callbacks)

class Callback(object):

"""Abstract base class used to build new callbacks.

"""

def __init__(self):

self.validation_data = None

def set_params(self, params):

self.params = params

def set_model(self, model):

self.model = model

def set_optimizer(self, optimizer):

self.optimizer = optimizer

def on_iter_begin(self, iter, logs=None):

pass

def on_iter_end(self, iter, logs=None):

pass

def on_train_begin(self, logs=None):

pass

def on_train_end(self, logs=None):

pass

class BaseLogger(Callback):

def __init__(self, period=10):

super(BaseLogger, self).__init__()

self.period = period

def _reset(self):

self.totals = {}

def on_train_begin(self, logs=None):

self.totals = {}

def on_iter_end(self, iter, logs=None):

logs = logs or {}

#(iter - 1) // iters_per_epoch + 1

for k, v in logs.items():

if k in self.totals.keys():

self.totals[k] += v

else:

self.totals[k] = v

if iter % self.period == 0 and ParallelEnv().local_rank == 0:

for k in self.totals:

logs[k] = self.totals[k] / self.period

self._reset()

class TrainLogger(Callback):

def __init__(self, log_freq=10):

self.log_freq = log_freq

def _calculate_eta(self, remaining_iters, speed):

if remaining_iters < 0:

remaining_iters = 0

remaining_time = int(remaining_iters * speed)

result = "{:0>2}:{:0>2}:{:0>2}"

arr = []

for i in range(2, -1, -1):

arr.append(int(remaining_time / 60**i))

remaining_time %= 60**i

return result.format(*arr)

def on_iter_end(self, iter, logs=None):

if iter % self.log_freq == 0 and ParallelEnv().local_rank == 0:

total_iters = self.params["total_iters"]

iters_per_epoch = self.params["iters_per_epoch"]

remaining_iters = total_iters - iter

eta = self._calculate_eta(remaining_iters, logs["batch_cost"])

current_epoch = (iter - 1) // self.params["iters_per_epoch"] + 1

loss = logs["loss"]

lr = self.optimizer.get_lr()

batch_cost = logs["batch_cost"]

reader_cost = logs["reader_cost"]

logger.info(

"[TRAIN] epoch={}, iter={}/{}, loss={:.4f}, lr={:.6f}, batch_cost={:.4f}, reader_cost={:.4f} | ETA {}"

.format(current_epoch, iter, total_iters, loss, lr, batch_cost,

reader_cost, eta))

class ProgbarLogger(Callback):

def __init__(self):

super(ProgbarLogger, self).__init__()

def on_train_begin(self, logs=None):

self.verbose = self.params["verbose"]

self.total_iters = self.params["total_iters"]

self.target = self.params["total_iters"]

self.progbar = Progbar(target=self.target, verbose=self.verbose)

self.seen = 0

self.log_values = []

def on_iter_begin(self, iter, logs=None):

#self.seen = 0

if self.seen < self.target:

self.log_values = []

def on_iter_end(self, iter, logs=None):

logs = logs or {}

self.seen += 1

for k in self.params['metrics']:

if k in logs:

self.log_values.append((k, logs[k]))

#if self.verbose and self.seen < self.target and ParallelEnv.local_rank == 0:

#print(self.log_values)

if self.seen < self.target:

self.progbar.update(self.seen, self.log_values)

class ModelCheckpoint(Callback):

def __init__(self,

save_dir,

monitor="miou",

save_best_only=False,

save_params_only=True,

mode="max",

period=1):

super(ModelCheckpoint, self).__init__()

self.monitor = monitor

self.save_dir = save_dir

self.save_best_only = save_best_only

self.save_params_only = save_params_only

self.period = period

self.iters_since_last_save = 0

if mode == "min":

self.monitor_op = np.less

self.best = np.Inf

elif mode == "max":

self.monitor_op = np.greater

self.best = -np.Inf

else:

raise RuntimeError("`mode` is neither \"min\" nor \"max\"!")

def on_train_begin(self, logs=None):

self.verbose = self.params["verbose"]

save_dir = self.save_dir

if not os.path.isdir(save_dir):

if os.path.exists(save_dir):

os.remove(save_dir)

os.makedirs(save_dir)

def on_iter_end(self, iter, logs=None):

logs = logs or {}

self.iters_since_last_save += 1

current_save_dir = os.path.join(self.save_dir, "iter_{}".format(iter))

current_save_dir = os.path.abspath(current_save_dir)

#if self.iters_since_last_save % self.period and ParallelEnv().local_rank == 0:

#self.iters_since_last_save = 0

if iter % self.period == 0 and ParallelEnv().local_rank == 0:

if self.verbose > 0:

print("iter {iter_num}: saving model to {path}".format(

iter_num=iter, path=current_save_dir))

paddle.save(self.model.state_dict(),

os.path.join(current_save_dir, 'model.pdparams'))

if not self.save_params_only:

paddle.save(self.optimizer.state_dict(),

os.path.join(current_save_dir, 'model.pdopt'))

class VisualDL(Callback):

def __init__(self, log_dir="./log", freq=1):

super(VisualDL, self).__init__()

self.log_dir = log_dir

self.freq = freq

def on_train_begin(self, logs=None):

self.writer = LogWriter(self.log_dir)

def on_iter_end(self, iter, logs=None):

logs = logs or {}

if iter % self.freq == 0 and ParallelEnv().local_rank == 0:

for k, v in logs.items():

self.writer.add_scalar("Train/{}".format(k), v, iter)

self.writer.flush()

def on_train_end(self, logs=None):

self.writer.close()

import os

import numpy as np

import time

import paddle

import paddle.nn.functional as F

from paddleseg.utils import metrics, TimeAverager, calculate_eta, logger, progbar

from paddleseg.core import infer

np.set_printoptions(suppress=True)

def evaluate(model,

eval_dataset,

aug_eval=False,

scales=1.0,

flip_horizontal=False,

flip_vertical=False,

is_slide=False,

stride=None,

crop_size=None,

precision='fp32',

amp_level='O1',

num_workers=0,

print_detail=True,

auc_roc=False):

"""

Launch evalution.

Args:

model（nn.Layer): A semantic segmentation model.

eval_dataset (paddle.io.Dataset): Used to read and process validation datasets.

aug_eval (bool, optional): Whether to use mulit-scales and flip augment for evaluation. Default: False.

scales (list|float, optional): Scales for augment. It is valid when `aug_eval` is True. Default: 1.0.

flip_horizontal (bool, optional): Whether to use flip horizontally augment. It is valid when `aug_eval` is True. Default: True.

flip_vertical (bool, optional): Whether to use flip vertically augment. It is valid when `aug_eval` is True. Default: False.

is_slide (bool, optional): Whether to evaluate by sliding window. Default: False.

stride (tuple|list, optional): The stride of sliding window, the first is width and the second is height.

It should be provided when `is_slide` is True.

crop_size (tuple|list, optional): The crop size of sliding window, the first is width and the second is height.

It should be provided when `is_slide` is True.

precision (str, optional): Use AMP if precision='fp16'. If precision='fp32', the evaluation is normal.

amp_level (str, optional): Auto mixed precision level. Accepted values are “O1” and “O2”: O1 represent mixed precision, the input data type of each operator will be casted by white_list and black_list; O2 represent Pure fp16, all operators parameters and input data will be casted to fp16, except operators in black_list, don’t support fp16 kernel and batchnorm. Default is O1(amp)

num_workers (int, optional): Num workers for data loader. Default: 0.

print_detail (bool, optional): Whether to print detailed information about the evaluation process. Default: True.

auc_roc(bool, optional): whether add auc_roc metric

Returns:

float: The mIoU of validation datasets.

float: The accuracy of validation datasets.

"""

model.eval()

nranks = paddle.distributed.ParallelEnv().nranks

local_rank = paddle.distributed.ParallelEnv().local_rank

if nranks > 1:

\# Initialize parallel environment if not done.

if not paddle.distributed.parallel.parallel_helper._is_parallel_ctx_initialized(

):

paddle.distributed.init_parallel_env()

batch_sampler = paddle.io.DistributedBatchSampler(

eval_dataset, batch_size=1, shuffle=False, drop_last=False)

loader = paddle.io.DataLoader(

eval_dataset,

batch_sampler=batch_sampler,

num_workers=num_workers,

return_list=True, )

total_iters = len(loader)

intersect_area_all = paddle.zeros([1], dtype='int64')

pred_area_all = paddle.zeros([1], dtype='int64')

label_area_all = paddle.zeros([1], dtype='int64')

logits_all = None

label_all = None

if print_detail:

logger.info("Start evaluating (total_samples: {}, total_iters: {})...".

format(len(eval_dataset), total_iters))

#TODO(chenguowei): fix log print error with multi-gpus

progbar_val = progbar.Progbar(

target=total_iters, verbose=1 if nranks < 2 else 2)

reader_cost_averager = TimeAverager()

batch_cost_averager = TimeAverager()

batch_start = time.time()

with paddle.no_grad():

for iter, data in enumerate(loader):

reader_cost_averager.record(time.time() - batch_start)

label = data['label'].astype('int64')

if aug_eval:

if precision == 'fp16':

with paddle.amp.auto_cast(

level=amp_level,

enable=True,

custom_white_list={

"elementwise_add", "batch_norm",

"sync_batch_norm"

},

custom_black_list={'bilinear_interp_v2'}):

pred, logits = infer.aug_inference(

model,

data['img'],

trans_info=data['trans_info'],

scales=scales,

flip_horizontal=flip_horizontal,

flip_vertical=flip_vertical,

is_slide=is_slide,

stride=stride,

crop_size=crop_size)

else:

pred, logits = infer.aug_inference(

model,

data['img'],

trans_info=data['trans_info'],

scales=scales,

flip_horizontal=flip_horizontal,

flip_vertical=flip_vertical,

is_slide=is_slide,

stride=stride,

crop_size=crop_size)

else:

if precision == 'fp16':

with paddle.amp.auto_cast(

level=amp_level,

enable=True,

custom_white_list={

"elementwise_add", "batch_norm",

"sync_batch_norm"

},

custom_black_list={'bilinear_interp_v2'}):

pred, logits = infer.inference(

model,

data['img'],

trans_info=data['trans_info'],

is_slide=is_slide,

stride=stride,

crop_size=crop_size)

else:

pred, logits = infer.inference(

model,

data['img'],

trans_info=data['trans_info'],

is_slide=is_slide,

stride=stride,

crop_size=crop_size)

intersect_area, pred_area, label_area = metrics.calculate_area(

pred,

label,

eval_dataset.num_classes,

ignore_index=eval_dataset.ignore_index)

\# Gather from all ranks

if nranks > 1:

intersect_area_list = []

pred_area_list = []

label_area_list = []

paddle.distributed.all_gather(intersect_area_list,

intersect_area)

paddle.distributed.all_gather(pred_area_list, pred_area)

paddle.distributed.all_gather(label_area_list, label_area)

\# Some image has been evaluated and should be eliminated in last iter

if (iter + 1) * nranks > len(eval_dataset):

valid = len(eval_dataset) - iter * nranks

intersect_area_list = intersect_area_list[:valid]

pred_area_list = pred_area_list[:valid]

label_area_list = label_area_list[:valid]

for i in range(len(intersect_area_list)):

intersect_area_all = intersect_area_all + intersect_area_list[

i]

pred_area_all = pred_area_all + pred_area_list[i]

label_area_all = label_area_all + label_area_list[i]

else:

intersect_area_all = intersect_area_all + intersect_area

pred_area_all = pred_area_all + pred_area

label_area_all = label_area_all + label_area

if auc_roc:

logits = F.softmax(logits, axis=1)

if logits_all is None:

logits_all = logits.numpy()

label_all = label.numpy()

else:

logits_all = np.concatenate(

[logits_all, logits.numpy()]) # (KN, C, H, W)

label_all = np.concatenate([label_all, label.numpy()])

batch_cost_averager.record(

time.time() - batch_start, num_samples=len(label))

batch_cost = batch_cost_averager.get_average()

reader_cost = reader_cost_averager.get_average()

if local_rank == 0 and print_detail:

progbar_val.update(iter + 1, [('batch_cost', batch_cost),

('reader cost', reader_cost)])

reader_cost_averager.reset()

batch_cost_averager.reset()

batch_start = time.time()

metrics_input = (intersect_area_all, pred_area_all, label_area_all)

class_iou, miou = metrics.mean_iou(*metrics_input)

acc, class_precision, class_recall = metrics.class_measurement(

*metrics_input)

kappa = metrics.kappa(*metrics_input)

class_dice, mdice = metrics.dice(*metrics_input)

if auc_roc:

auc_roc = metrics.auc_roc(

logits_all, label_all, num_classes=eval_dataset.num_classes)

auc_infor = ' Auc_roc: {:.4f}'.format(auc_roc)

if print_detail:

infor = "[EVAL] #Images: {} mIoU: {:.4f} Acc: {:.4f} Kappa: {:.4f} Dice: {:.4f}".format(

len(eval_dataset), miou, acc, kappa, mdice)

infor = infor + auc_infor if auc_roc else infor

logger.info(infor)

logger.info("[EVAL] Class IoU: \n" + str(np.round(class_iou, 4)))

logger.info("[EVAL] Class Precision: \n" + str(

np.round(class_precision, 4)))

logger.info("[EVAL] Class Recall: \n" + str(np.round(class_recall, 4)))

return miou, acc, class_iou, class_precision, kappa

import os

import time

from collections import deque

import shutil

import paddle

import paddle.nn.functional as F

from paddleseg.utils import (TimeAverager, calculate_eta, resume, logger,

worker_init_fn, train_profiler, op_flops_funs)

from paddleseg.core.val import evaluate

def loss_computation(logits_list, labels, edges, losses):

check_logits_losses(logits_list, losses)

loss_list = []

for i in range(len(logits_list)):

logits = logits_list[i]

loss_i = losses['types'][i]

coef_i = losses['coef'][i]

if loss_i.__class__.__name__ in ('BCELoss', ) and loss_i.edge_label:

\# Use edges as labels According to loss type.

loss_list.append(coef_i * loss_i(logits, edges))

elif loss_i.__class__.__name__ == 'MixedLoss':

mixed_loss_list = loss_i(logits, labels)

for mixed_loss in mixed_loss_list:

loss_list.append(coef_i * mixed_loss)

elif loss_i.__class__.__name__ in ("KLLoss", ):

loss_list.append(coef_i *

loss_i(logits_list[0], logits_list[1].detach()))

else:

loss_list.append(coef_i * loss_i(logits, labels))

return loss_list

def train(model,

train_dataset,

val_dataset=None,

optimizer=None,

save_dir='output',

iters=10000,

batch_size=2,

resume_model=None,

save_interval=1000,

log_iters=10,

num_workers=0,

use_vdl=False,

losses=None,

keep_checkpoint_max=5,

test_config=None,

precision='fp32',

amp_level='O1',

profiler_options=None,

to_static_training=False):

"""

Launch training.

Args:

model（nn.Layer): A semantic segmentation model.

train_dataset (paddle.io.Dataset): Used to read and process training datasets.

val_dataset (paddle.io.Dataset, optional): Used to read and process validation datasets.

optimizer (paddle.optimizer.Optimizer): The optimizer.

save_dir (str, optional): The directory for saving the model snapshot. Default: 'output'.

iters (int, optional): How may iters to train the model. Defualt: 10000.

batch_size (int, optional): Mini batch size of one gpu or cpu. Default: 2.

resume_model (str, optional): The path of resume model.

save_interval (int, optional): How many iters to save a model snapshot once during training. Default: 1000.

log_iters (int, optional): Display logging information at every log_iters. Default: 10.

num_workers (int, optional): Num workers for data loader. Default: 0.

use_vdl (bool, optional): Whether to record the data to VisualDL during training. Default: False.

losses (dict, optional): A dict including 'types' and 'coef'. The length of coef should equal to 1 or len(losses['types']).

The 'types' item is a list of object of paddleseg.models.losses while the 'coef' item is a list of the relevant coefficient.

keep_checkpoint_max (int, optional): Maximum number of checkpoints to save. Default: 5.

test_config(dict, optional): Evaluation config.

precision (str, optional): Use AMP if precision='fp16'. If precision='fp32', the training is normal.

amp_level (str, optional): Auto mixed precision level. Accepted values are “O1” and “O2”: O1 represent mixed precision,

the input data type of each operator will be casted by white_list and black_list; O2 represent Pure fp16, all operators

parameters and input data will be casted to fp16, except operators in black_list, don’t support fp16 kernel and batchnorm. Default is O1(amp)

profiler_options (str, optional): The option of train profiler.

to_static_training (bool, optional): Whether to use @to_static for training.

"""

model.train()

nranks = paddle.distributed.ParallelEnv().nranks

local_rank = paddle.distributed.ParallelEnv().local_rank

start_iter = 0

if resume_model is not None:

start_iter = resume(model, optimizer, resume_model)

if not os.path.isdir(save_dir):

if os.path.exists(save_dir):

os.remove(save_dir)

os.makedirs(save_dir, exist_ok=True)

\# use amp

if precision == 'fp16':

logger.info('use AMP to train. AMP level = {}'.format(amp_level))

scaler = paddle.amp.GradScaler(init_loss_scaling=1024)

if amp_level == 'O2':

model, optimizer = paddle.amp.decorate(

models=model,

optimizers=optimizer,

level='O2',

save_dtype='float32')

if nranks > 1:

paddle.distributed.fleet.init(is_collective=True)

optimizer = paddle.distributed.fleet.distributed_optimizer(

optimizer) # The return is Fleet object

ddp_model = paddle.distributed.fleet.distributed_model(model)

batch_sampler = paddle.io.DistributedBatchSampler(

train_dataset, batch_size=batch_size, shuffle=True, drop_last=True)

loader = paddle.io.DataLoader(

train_dataset,

batch_sampler=batch_sampler,

num_workers=num_workers,

return_list=True,

worker_init_fn=worker_init_fn, )

if use_vdl:

from visualdl import LogWriter

log_writer = LogWriter(save_dir)

if to_static_training:

model = paddle.jit.to_static(model)

logger.info("Successfully applied @to_static")

avg_loss = 0.0

avg_loss_list = []

iters_per_epoch = len(batch_sampler)

best_mean_iou = -1.0

best_model_iter = -1

reader_cost_averager = TimeAverager()

batch_cost_averager = TimeAverager()

save_models = deque()

batch_start = time.time()

iter = start_iter

while iter < iters:

for data in loader:

iter += 1

if iter > iters:

version = paddle.__version__

if version == '2.1.2':

continue

else:

break

reader_cost_averager.record(time.time() - batch_start)

images = data['img']

labels = data['label'].astype('int64')

edges = None

if 'edge' in data.keys():

edges = data['edge'].astype('int64')

if hasattr(model, 'data_format') and model.data_format == 'NHWC':

images = images.transpose((0, 2, 3, 1))

if precision == 'fp16':

with paddle.amp.auto_cast(

level=amp_level,

enable=True,

custom_white_list={

"elementwise_add", "batch_norm", "sync_batch_norm"

},

custom_black_list={'bilinear_interp_v2'}):

logits_list = ddp_model(images) if nranks > 1 else model(

images)

loss_list = loss_computation(

logits_list=logits_list,

labels=labels,

edges=edges,

losses=losses)

loss = sum(loss_list)

scaled = scaler.scale(loss) # scale the loss

scaled.backward() # do backward

if isinstance(optimizer, paddle.distributed.fleet.Fleet):

scaler.minimize(optimizer.user_defined_optimizer, scaled)

else:

scaler.minimize(optimizer, scaled) # update parameters

else:

logits_list = ddp_model(images) if nranks > 1 else model(images)

loss_list = loss_computation(

logits_list=logits_list,

labels=labels,

edges=edges,

losses=losses)

loss = sum(loss_list)

loss.backward()

\# if the optimizer is ReduceOnPlateau, the loss is the one which has been pass into step.

if isinstance(optimizer, paddle.optimizer.lr.ReduceOnPlateau):

optimizer.step(loss)

else:

optimizer.step()

lr = optimizer.get_lr()

\# update lr

if isinstance(optimizer, paddle.distributed.fleet.Fleet):

lr_sche = optimizer.user_defined_optimizer._learning_rate

else:

lr_sche = optimizer._learning_rate

if isinstance(lr_sche, paddle.optimizer.lr.LRScheduler):

lr_sche.step()

train_profiler.add_profiler_step(profiler_options)

model.clear_gradients()

avg_loss += loss.numpy()[0]

if not avg_loss_list:

avg_loss_list = [l.numpy() for l in loss_list]

else:

for i in range(len(loss_list)):

avg_loss_list[i] += loss_list[i].numpy()

batch_cost_averager.record(

time.time() - batch_start, num_samples=batch_size)

if (iter) % log_iters == 0 and local_rank == 0:

avg_loss /= log_iters

avg_loss_list = [l[0] / log_iters for l in avg_loss_list]

remain_iters = iters - iter

avg_train_batch_cost = batch_cost_averager.get_average()

avg_train_reader_cost = reader_cost_averager.get_average()

eta = calculate_eta(remain_iters, avg_train_batch_cost)

logger.info(

"[TRAIN] epoch: {}, iter: {}/{}, loss: {:.4f}, lr: {:.6f}, batch_cost: {:.4f}, reader_cost: {:.5f}, ips: {:.4f} samples/sec | ETA {}"

.format((iter - 1

) // iters_per_epoch + 1, iter, iters, avg_loss,

lr, avg_train_batch_cost, avg_train_reader_cost,

batch_cost_averager.get_ips_average(), eta))

if use_vdl:

log_writer.add_scalar('Train/loss', avg_loss, iter)

\# Record all losses if there are more than 2 losses.

if len(avg_loss_list) > 1:

avg_loss_dict = {}

for i, value in enumerate(avg_loss_list):

avg_loss_dict['loss_' + str(i)] = value

for key, value in avg_loss_dict.items():

log_tag = 'Train/' + key

log_writer.add_scalar(log_tag, value, iter)

log_writer.add_scalar('Train/lr', lr, iter)

log_writer.add_scalar('Train/batch_cost',

avg_train_batch_cost, iter)

log_writer.add_scalar('Train/reader_cost',

avg_train_reader_cost, iter)

avg_loss = 0.0

avg_loss_list = []

reader_cost_averager.reset()

batch_cost_averager.reset()

if (iter % save_interval == 0 or

iter == iters) and (val_dataset is not None):

num_workers = 1 if num_workers > 0 else 0

if test_config is None:

test_config = {}

mean_iou, acc, _, _, _ = evaluate(

model,

val_dataset,

num_workers=num_workers,

precision=precision,

amp_level=amp_level,

**test_config)

model.train()

if (iter % save_interval == 0 or iter == iters) and local_rank == 0:

current_save_dir = os.path.join(save_dir,

"iter_{}".format(iter))

if not os.path.isdir(current_save_dir):

os.makedirs(current_save_dir)

paddle.save(model.state_dict(),

os.path.join(current_save_dir, 'model.pdparams'))

paddle.save(optimizer.state_dict(),

os.path.join(current_save_dir, 'model.pdopt'))

save_models.append(current_save_dir)

if len(save_models) > keep_checkpoint_max > 0:

model_to_remove = save_models.popleft()

shutil.rmtree(model_to_remove)

if val_dataset is not None:

if mean_iou > best_mean_iou:

best_mean_iou = mean_iou

best_model_iter = iter

best_model_dir = os.path.join(save_dir, "best_model")

paddle.save(

model.state_dict(),

os.path.join(best_model_dir, 'model.pdparams'))

logger.info(

'[EVAL] The model with the best validation mIoU ({:.4f}) was saved at iter {}.'

.format(best_mean_iou, best_model_iter))

if use_vdl:

log_writer.add_scalar('Evaluate/mIoU', mean_iou, iter)

log_writer.add_scalar('Evaluate/Acc', acc, iter)

batch_start = time.time()

\# Calculate flops.

if local_rank == 0 and not (precision == 'fp16' and amp_level == 'O2'):

_, c, h, w = images.shape

_ = paddle.flops(

model, [1, c, h, w],

custom_ops={paddle.nn.SyncBatchNorm: op_flops_funs.count_syncbn})

\# Sleep for half a second to let dataloader release resources.

time.sleep(0.5)

if use_vdl:

log_writer.close()

import os

import math

import cv2

import numpy as np

import paddle

from paddleseg import utils

from paddleseg.core import infer

from paddleseg.utils import logger, progbar, visualize

def mkdir(path):

sub_dir = os.path.dirname(path)

if not os.path.exists(sub_dir):

os.makedirs(sub_dir)

def partition_list(arr, m):

"""split the list 'arr' into m pieces"""

n = int(math.ceil(len(arr) / float(m)))

return [arr[i:i + n] for i in range(0, len(arr), n)]

def preprocess(im_path, transforms):

data = {}

data['img'] = im_path

data = transforms(data)

data['img'] = data['img'][np.newaxis, ...]

data['img'] = paddle.to_tensor(data['img'])

return data

def predict(model,

model_path,

transforms,

image_list,

image_dir=None,

save_dir='output',

aug_pred=False,

scales=1.0,

flip_horizontal=True,

flip_vertical=False,

is_slide=False,

stride=None,

crop_size=None,

custom_color=None):

"""

predict and visualize the image_list.

Args:

model (nn.Layer): Used to predict for input image.

model_path (str): The path of pretrained model.

transforms (transform.Compose): Preprocess for input image.

image_list (list): A list of image path to be predicted.

image_dir (str, optional): The root directory of the images predicted. Default: None.

save_dir (str, optional): The directory to save the visualized results. Default: 'output'.

aug_pred (bool, optional): Whether to use mulit-scales and flip augment for predition. Default: False.

scales (list|float, optional): Scales for augment. It is valid when `aug_pred` is True. Default: 1.0.

flip_horizontal (bool, optional): Whether to use flip horizontally augment. It is valid when `aug_pred` is True. Default: True.

flip_vertical (bool, optional): Whether to use flip vertically augment. It is valid when `aug_pred` is True. Default: False.

is_slide (bool, optional): Whether to predict by sliding window. Default: False.

stride (tuple|list, optional): The stride of sliding window, the first is width and the second is height.

It should be provided when `is_slide` is True.

crop_size (tuple|list, optional): The crop size of sliding window, the first is width and the second is height.

It should be provided when `is_slide` is True.

custom_color (list, optional): Save images with a custom color map. Default: None, use paddleseg's default color map.

"""

utils.utils.load_entire_model(model, model_path)

model.eval()

nranks = paddle.distributed.get_world_size()

local_rank = paddle.distributed.get_rank()

if nranks > 1:

img_lists = partition_list(image_list, nranks)

else:

img_lists = [image_list]

added_saved_dir = os.path.join(save_dir, 'added_prediction')

pred_saved_dir = os.path.join(save_dir, 'pseudo_color_prediction')

logger.info("Start to predict...")

progbar_pred = progbar.Progbar(target=len(img_lists[0]), verbose=1)

color_map = visualize.get_color_map_list(256, custom_color=custom_color)

with paddle.no_grad():

for i, im_path in enumerate(img_lists[local_rank]):

data = preprocess(im_path, transforms)

if aug_pred:

pred, _ = infer.aug_inference(

model,

data['img'],

trans_info=data['trans_info'],

scales=scales,

flip_horizontal=flip_horizontal,

flip_vertical=flip_vertical,

is_slide=is_slide,

stride=stride,

crop_size=crop_size)

else:

pred, _ = infer.inference(

model,

data['img'],

trans_info=data['trans_info'],

is_slide=is_slide,

stride=stride,

crop_size=crop_size)

pred = paddle.squeeze(pred)

pred = pred.numpy().astype('uint8')

\# get the saved name

if image_dir is not None:

im_file = im_path.replace(image_dir, '')

else:

im_file = os.path.basename(im_path)

if im_file[0] == '/' or im_file[0] == '\\':

im_file = im_file[1:]

\# save added image

added_image = utils.visualize.visualize(

im_path, pred, color_map, weight=0.6)

added_image_path = os.path.join(added_saved_dir, im_file)

mkdir(added_image_path)

cv2.imwrite(added_image_path, added_image)

\# save pseudo color prediction

pred_mask = utils.visualize.get_pseudo_color_map(pred, color_map)

pred_saved_path = os.path.join(

pred_saved_dir, os.path.splitext(im_file)[0] + ".png")

mkdir(pred_saved_path)

pred_mask.save(pred_saved_path)

progbar_pred.update(i + 1)

import os

import numpy as np

from PIL import Image

import cv2

import os

\# merge

def blend_two_images(label, image, resault):

img1 = Image.open(label)

img1 = img1.convert('RGBA')

img2 = Image.open(image)

img2 = img2.convert('RGBA')

img = Image.blend(img1, img2, 0.4)

img.save(resault)

return

def predictSinglePicture(filename):

print(filename)

pythonfile = "/home/jack/Downloads/thyroidProgram/interface/Semantic/PaddleSeg-release-2.6/deploy/python/infer.py"

configfile = "/home/jack/Downloads/thyroidProgram/interface/Semantic/model1111/deploy.yaml"

savedirfile = "/home/jack/Downloads/thyroidProgram/interface/Semantic/output/"

command = "python3 "+pythonfile+" --config "+configfile+" --image_path "+filename+" --save_dir "+savedirfile

os.system(command)

source_img_path = "/home/jack/Downloads/thyroidProgram/interface/Semantic/output/1.png"

target_img_path = "/home/jack/Downloads/thyroidProgram/interface/Semantic/output/2.png"

img = Image.open(source_img_path)

img = cv2.cvtColor(np.asarray(img), cv2.COLOR_RGB2BGR)

rowNum, colNum = img.shape[:2]

contentlist = []

contentlist.append({"name":"膜玻璃核","num":0})

contentlist.append({"name": "肿瘤细胞", "num": 0})

contentlist.append({"name": "核沟", "num": 0})

for x in range(rowNum):

for y in range(colNum):

a = img[x, y].tolist()

if img[x, y].tolist() == [0, 0, 0]: # background

img[x, y] = np.array([17, 17, 17]);

if img[x, y].tolist() == [1, 1, 1]: # background

img[x, y] = np.array([85, 119, 221]);

contentlist[0]["num"] = contentlist[0]["num"]+1

if img[x, y].tolist() == [2, 2, 2]: # background

img[x, y] = np.array([17, 0, 153]);

contentlist[1]["num"] = contentlist[1]["num"]+1

if img[x, y].tolist() == [3, 3, 3]: # background

img[x, y] = np.array([51, 170, 68]);

contentlist[2]["num"] = contentlist[2]["num"]+1

cv2.imwrite(target_img_path, img);

merge_img_path = "/home/jack/Downloads/thyroidProgram/interface/Semantic/output/merge.png"

blend_two_images(filename, target_img_path, merge_img_path)

list = []

for item in contentlist:

if item["num"]>100:

list.append(item["name"])

return list

import uvicorn

from fastapi import FastAPI, File, UploadFile, Form

import os

from Semantic import main as sematic

from fastapi.responses import StreamingResponse

from fastapi.middleware.cors import CORSMiddleware

from fastapi.responses import FileResponse

import base64

app = FastAPI()

app.add_middleware(

CORSMiddleware,

\# 允许跨域的源列表，例如 ["http://www.example.org"] 等等，["*"] 表示允许任何源

allow_origins=["*"],

\# 跨域请求是否支持 cookie，默认是 False，如果为 True，allow_origins 必须为具体的源，不可以是 ["*"]

allow_credentials=False,

\# 允许跨域请求的 HTTP 方法列表，默认是 ["GET"]

allow_methods=["*"],

\# 允许跨域请求的 HTTP 请求头列表，默认是 []，可以使用 ["*"] 表示允许所有的请求头

\# 当然 Accept、Accept-Language、Content-Language 以及 Content-Type 总之被允许的

allow_headers=["*"],

\# 可以被浏览器访问的响应头, 默认是 []，一般很少指定

\# expose_headers=["*"]

\# 设定浏览器缓存 CORS 响应的最长时间，单位是秒。默认为 600，一般也很少指定

\# max_age=1000

)

@app.post("/arithmetic/upload/file/", summary="上传图片")

async def upload_image(

file: UploadFile = File(...)

):

save_dir = f"/home/jack/Downloads/thyroidProgram/interface/Semantic/picture/"

if not os.path.exists(save_dir):

os.mkdir(save_dir)

contents = await file.read()

\# filename = save_dir + file.filename

filename = save_dir + "1.png"

with open(filename, "wb") as f:

\# 2.3 将获取的fileb文件内容，写入到新文件中

f.write(contents)

list = sematic.predictSinglePicture(filename)

newname = "/home/jack/Downloads/thyroidProgram/interface/Semantic/output/"+ str(list) +".png"

oldname = '/home/jack/Downloads/thyroidProgram/interface/Semantic/output/merge.png'

os.rename(oldname, newname)

\# file_like = open(newname, mode="rb")

\# return StreamingResponse(file_like, media_type="image/jpg" )

\# return FileResponse(newname, filename=str(list))

with open(newname, 'rb') as img:

\# 使用base64进行编码

b64encode = base64.b64encode(img.read())

s = b64encode.decode()

b64_encode = 'data:image/png;base64,%s' % s

resault = { "img": b64_encode,"list":list}

return resault

if __name__ == '__main__':

uvicorn.run(app,host="192.168.100.32", port=8001)
